# Apoptosis-induced nuclear expulsion in tumor cells drives S100a4-mediated metastatic outgrowth through the RAGE pathway

**DOI:** 10.1038/s43018-023-00524-z

**Published:** 2023-03-27

**Authors:** Woo-Yong Park, Justin M. Gray, Ronald J. Holewinski, Thorkell Andresson, Jae Young So, Carmelo Carmona-Rivera, M. Christine Hollander, Howard H. Yang, Maxwell Lee, Mariana J. Kaplan, Steven D. Cappell, Li Yang

**Affiliations:** 1 Laboratory of Cancer Biology and Genetics, Center for Cancer Research, National Cancer Institute, National Institutes of Health, Bethesda, MD USA; 2 Department of Biology, Johns Hopkins University, Baltimore, MD USA; 3 Protein Mass Spectrometry Group, Center for Cancer Research, National Cancer Institute, National Institutes of Health, Frederick, MD USA; 4 Systemic Autoimmunity Branch, National Institute of Arthritis and Musculoskeletal and Skin Diseases, National Institutes of Health, Bethesda, MD USA

**Keywords:** Cancer, Metastasis, Apoptosis, Breast cancer

## Abstract

Most tumor cells undergo apoptosis in circulation and at the metastatic organ sites due to host immune surveillance and a hostile microenvironment. It remains to be elucidated whether dying tumor cells have a direct effect on live tumor cells during the metastatic process and what the underlying mechanisms are. Here we report that apoptotic cancer cells enhance the metastatic outgrowth of surviving cells through Padi4-mediated nuclear expulsion. Tumor cell nuclear expulsion results in an extracellular DNA–protein complex that is enriched with receptor for advanced glycation endproducts (RAGE) ligands. The chromatin-bound RAGE ligand S100a4 activates RAGE receptors in neighboring surviving tumor cells, leading to Erk activation. In addition, we identified nuclear expulsion products in human patients with breast, bladder and lung cancer and a nuclear expulsion signature correlated with poor prognosis. Collectively, our study demonstrates how apoptotic cell death can enhance the metastatic outgrowth of neighboring live tumor cells.

## Abstract (web summary)

Park et al. show that in the process of metastasis, dying tumor cells enhance the outgrowth of their surviving counterparts via nuclear expulsion, resulting in an extracellular DNA–protein complex that activates RAGE receptors on tumor cells.

## Main

Metastasis is a process marked by massive amounts of cell death, with only the fittest tumor cells surviving to colonize distant organs^1^. There are different forms of cell death with distinct morphological, molecular and genetic features^2^. Apoptotic cell death, a seemingly beneficial therapeutic outcome, can, however, be harnessed by tumor cells to enhance metastatic functions^3^. In fact, high apoptotic indices have been correlated with poor prognosis in non-small cell lung cancer, lymphomas and glioblastoma^4–6^. The underlying mechanisms have been perplexing and it is now increasingly apparent that apoptotic cells influence nearby tumor cells through the release of mitogen signals^7^, extracellular vesicles^8,9^, inflammatory mediators^10^ and metabolites^11^. Yet, it remains unclear what happens to chromatin that is released from dying tumor cells and how it might affect nearby live tumor cells.

Chromatin containing citrullinated histone H3 (CitH3) can be released to the extracellular space by neutrophils through the formation of neutrophil extracellular traps (NETs), a process mediated by the activation of peptidylarginine deiminase 4 (Padi4)^12^. NET formation results in a complex of DNA, histones and other proteins such as proteases being released into the extracellular space where they trap and kill microbes^13^. Several studies implicate NETs in metastatic progression^14–17^, although it is not clear whether Padi4 and CitH3 are produced in cancer cells and what its function is in metastatic progression.

RAGE, a multi-ligand receptor, plays a pivotal role in cancer through engaging signaling cascades such as MAPK, AKT and nuclear factor (NF)-κB, which affect proliferation, apoptosis, autophagy and migration^18,19^. Several proteins bind RAGE, such as S100a4 and high-mobility group-binding proteins (HMGB)^18,20–22^, which play a pivotal role in tumor microenvironment-mediated metastatic progression^18,20,21^; however, whether tumor cells produce extracellular S100a4 or other RAGE ligands and the mechanisms of function remains to be investigated.

In this study we discover that metastatic breast cancer cells can undergo a previously unreported process that is characterized by nuclear expulsion, which releases chromatin and its associated proteins, which we name nuclear expulsion products (NEPs). We demonstrate that chromatin-bound S100a4 in NEPs activates the RAGE receptor on neighboring tumor cells and enhances their metastatic outgrowth in the lung. We further identify nuclear expulsion in human cancer cell lines and biopsies from patients with breast, bladder and lung cancer. Our studies demonstrate nuclear expulsion as a generalized phenotype in cancer, highlight its importance in metastatic spread and point to potential treatment options.

## Results

### Nuclear expulsion occurs in cancer cells in a Padi4-dependent manner

**Fig. 1:**
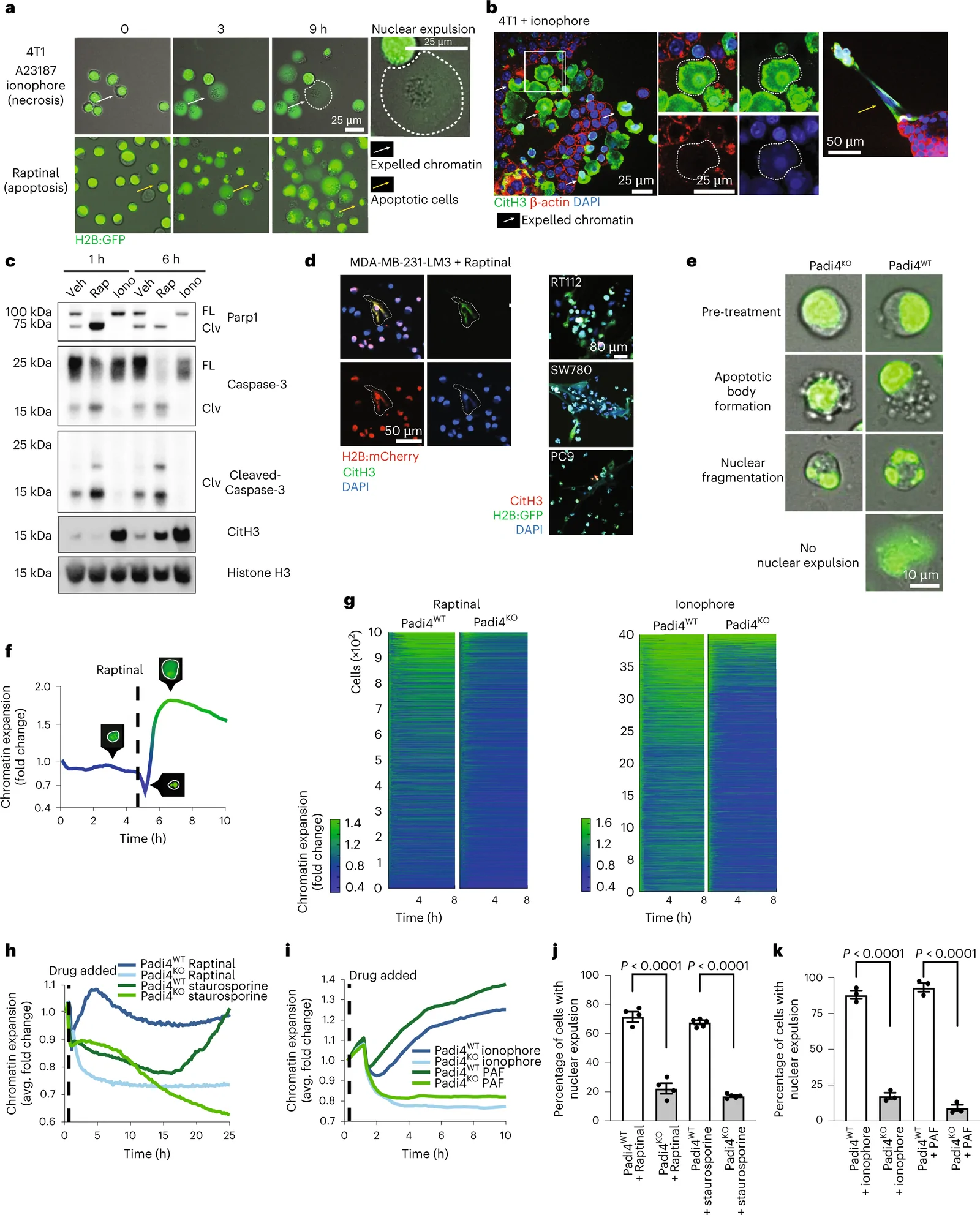
Apoptosis triggers nuclear expulsion in cancer cells in a Padi4-dependent manner. **a**, Time-lapse microscopy of nuclear expulsion in 4T1 cells expressing H2B-GFP upon A23187 ionophore or Raptinal treatment. White arrow, nuclear expulsion; yellow arrow, apoptosis; dotted line, expelled nuclear area. **b**, Immunofluorescence (IF) of nuclear expulsion (left and middle) and extracellular fiber-like DNA/chromatin structures of NEPs (right) in 4T1 treated with A23187 ionophore for 30 min. Cells were stained with CitH3, β-actin and 4,6-diamidino-2-phenylindole (DAPI). White arrows, nuclear expulsion; yellow arrow, fiber-like structure; white box, higher magnified images (middle); dotted line, expelled nuclear area. **c**, Western blot of PARP1, caspase-3 and CitH3 of 4T1 cells treated with Raptinal (Rap) or A23187 ionophore (Iono). FL, full length; Clv, cleaved; Veh, vehicle. **d**, IF of MDA-MB-231-LM3, PC9, RT112 and SW780 cells treated with Raptinal. Dotted line, expelled chromatin. Green indicates CitH3; red indicates H2B:mCherry for MDA-MB-231-LM3 (left). Green indicates H2B:GFP; red indicates CitH3 for PC9, RT112 and SW780 (right). **e**, Representative images of apoptotic bodies and nuclear fragmentation in H2B-GFP Paidi4^WT^ and Padi4^KO^ 4T1 cells and nuclear expulsion in Padi4^WT^ cells. **f**, Time-lapse of EO771-LMB cells going through apoptotic nuclear fragmentation followed by nuclear expulsion. The microscopic images representing baseline, condensation and decondensation are indicated in the figure. **g**, Histogram of chromatin expansion in EO771-LMB Padi4^WT^ and Padi4^KO^ cells tracked during treatment with Raptinal (1,000 cells) or A23187 ionophore (4,000 cells). Colors indicate chromatin expansion over the time course. **h**,**i**, Tracking the median chromatin expansion for thousands of EO771-LMB Padi4^WT^ and Padi4^KO^ cells treated with Raptinal and staurosporine (**h**) or with A23187 ionophore and PAF (**i**). **j**,**k**, Percentage of EO771-LMB cells undergoing nuclear expulsion with treatment of Raptinal (*n* = 8,148 of Padi4^WT^cells and *n* = 9,789 of Padi4^KO^ cells examined over four independent experiments) and staurosporine (*n* = 10,731 of Padi4^WT^cells and *n* = 17,638 of Padi4^KO^ examined over five independent experiments) (**j**) or A23187 ionophore (*n* = 11,656 of Padi4^WT^cells and *n* = 6,001 of Padi4^KO^ cells examined over three independent experiments) and PAF (*n* = 13,690 of Padi4^WT^cells and *n* = 9,772 of Padi4^KO^ cells examined over three independent experiments) (**k**). All data are represented as mean ± s.e.m. and *P* values are based on two-tailed Student’s *t*-test. Western blotting and IF were repeated at least twice and representative data are shown. Source data

**Extended Data Fig. 1:**
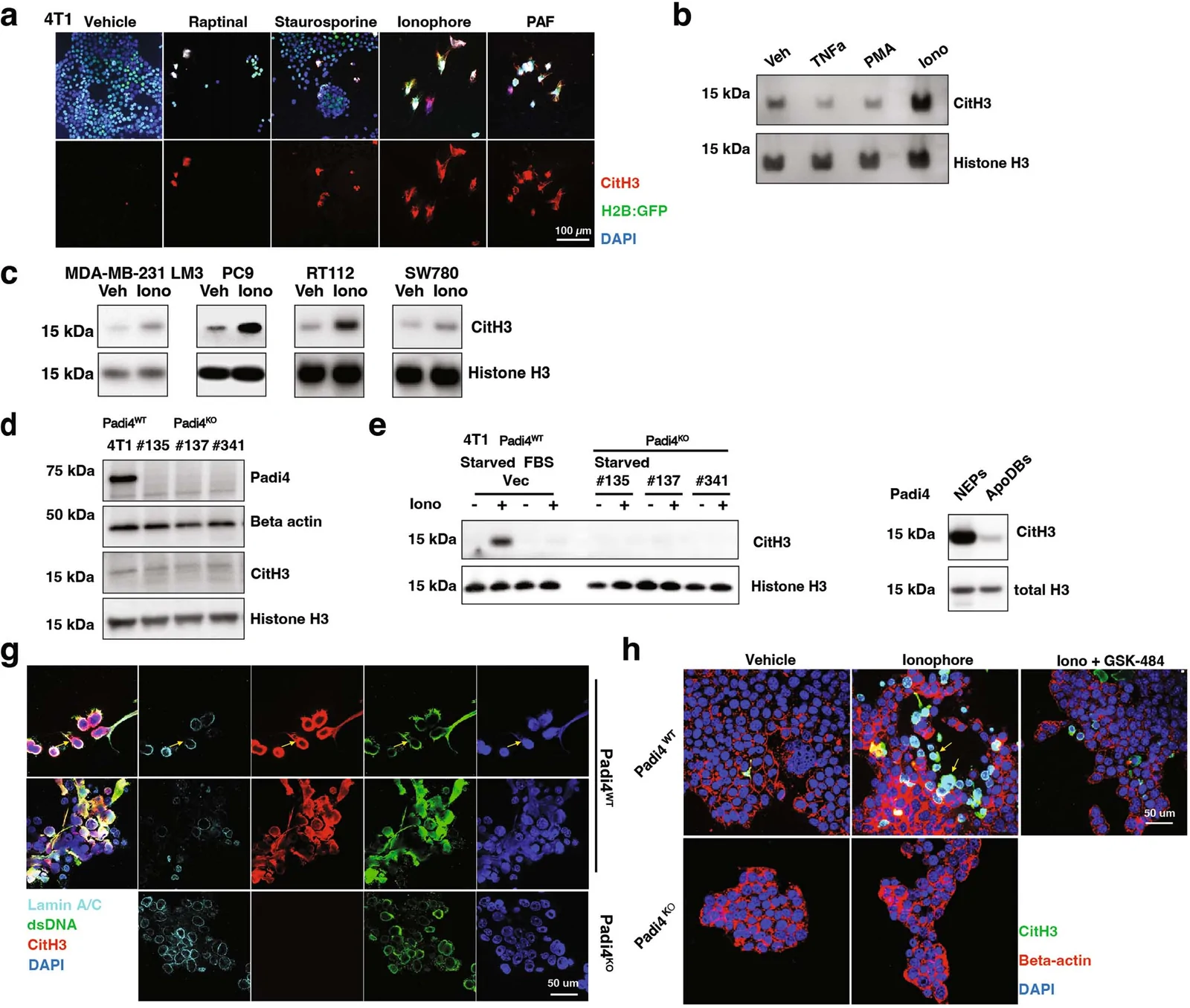
Apoptosis and A23187 ionophore trigger CitH3 in tumor cells in a Padi4 dependent manner. **a**. IF of NEPs from 4T1 cells with indicated stimuli. Green: H2B-GFP, Red: CitH3, Blue: DAPI. **b**. CitH3 Western blot of 4T1 cells treated with TNF-a, PMA and A23187 ionophore (Iono). **c**. CitH3 Western blots of various human cancer cell lines treated with A23187 ionphore. **d**. Padi4 Western blot for validating Padi4 deletion and CitH3 in 4T1 cells in untreated cells. **e**. CitH3 Western in Padi4^WT^ and Padi4^KO^ 4T1 cells treated with A23187 ionophore under starved condition. 10 % FBS treatment (FBS) in 4T1 cells used as normal condition. **f**. CitH3 Western in isolated NEPs or apoptotic debris (ApoDBs). **g**. IF of nuclear membrane disruption and extracellular double-strand DNA in Padi4^WT^ and Padi4^KO^ 4T1 cells. Cyan: Lamin A/C, Green: Double-strand DNA, Red: CitH3, Blue: DAPI. **h**. IF of Padi4^WT^ and Padi4^KO^ 4T1 upon A23187 ionophore treatment in combination with GSK-484. Green: CitH3, Red: Beta-actin, Blue: DAPI. Western blotting and IF were repeated at least twice and representative data are shown. Source data

Dying tumor cells generate extracellular vesicles, mitogen signals and metabolites influencing nearby tumor cells^7–11^. It is unclear what effect the chromatin released from dying tumor cells might have. To study this, we treated 4T1 cells with a variety of cell death-inducing reagents (Supplementary Table 1) and performed time-lapse imaging using H2B:GFP to mark the chromatin. We observed a phenotype characterized by nuclear expulsion with treatment of calcium ionophore A23187 (herein ionophore) (Fig. 1a and Supplementary Video 1) and Raptinal (Fig. 1a and Supplementary Video 2), as well as platelet-activating factor (PAF) (Extended Data Fig. 1a). The 4T1 cells produced web-like extracellular CitH3 and citrullinated histones detected by western blot (Extended Data Fig. 1a,b and Fig. 1b). Notably, 4T1 cells treated with Raptinal showed CitH3 along with common apoptosis hallmarks such as cleaved caspase-3 and PARP1 (Fig. 1c). To understand the human applications of nuclear expulsion, we also treated a variety of human cancer cell lines with ionophore and observed increased CitH3 in MDA-MB-231-LM3 cells, a lung metastatic derivative of MDA-MB-231, a lung cancer cell line PC9, as well as two bladder cancer cell lines, RT112 and SW780 (Extended Data Fig. 1c). Web-like citrullinated chromatin was also found when these cells were treated with Raptinal (Fig. 1d). MDA-MB-231-LM3 also displayed nuclear expulsion upon Raptinal treatment (Supplementary Video 3). Together, these findings suggest that apoptosis triggers nuclear expulsion in both mouse and human cancer cells and is likely a generalized phenotype.

The citrullinated chromatin suggests a role of Padi4. Indeed, Padi4 knockout (Padi4^KO^) diminished CitH3, with no nuclear expulsion or disruption of the nuclear envelope (Extended Data Fig. 1d–g and Supplementary Video 4), suggesting that nuclear expulsion is Padi4 dependent. Time-lapse imaging showed visual apoptosis hallmarks upon Raptinal treatment such as apoptotic body formation and nuclear fragmentation, which were followed by nuclear expulsion in Padi4 wild-type (Padi4^WT^) cells, whereas the Padi4^KO^ cells only underwent apoptosis but not nuclear expulsion (Fig. 1e and Supplementary Video 5). Additionally, GSK-484, which inhibits Padi4 enzymatic activity, blocked citrullination by ionophore (Extended Data Fig. 1h), suggesting that Padi4 is essential for H3 citrullination followed by nuclear expulsion.

**Extended Data Fig. 2:**
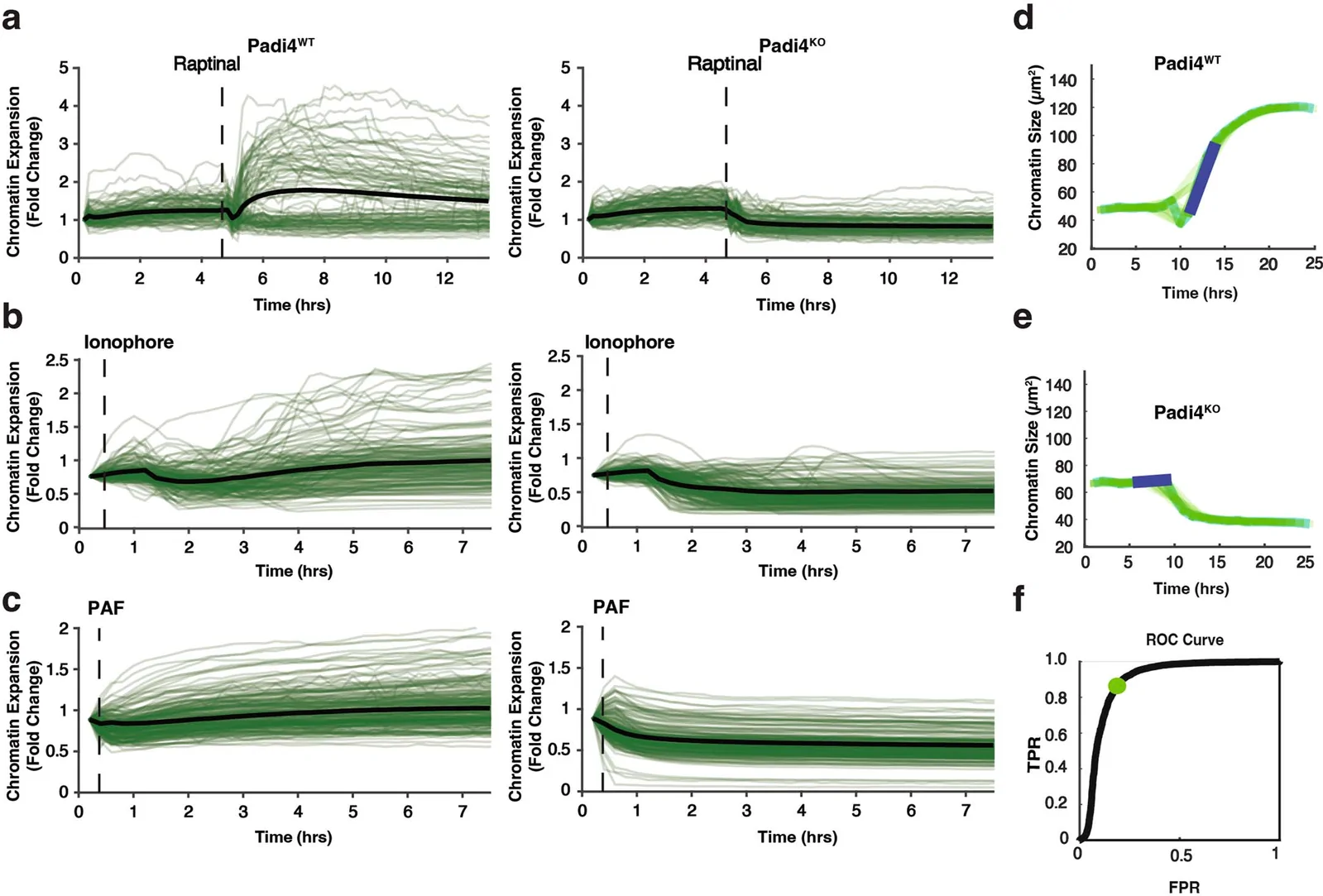
Time-lapse imaging, validation of nuclear expulsion by expulsion algorithm and CitH3. **a** - **c**. Individual traces showing chromatin expansion for 200 Padi4^WT^ and Padi4^KO^ H2B-GFP 4T1 cells treated with raptinal (**a**), A23187 ionophore (**b**) and PAF (**c**). **d** - **f**. Expulsion algorithm detects cells that went through nuclear expulsion. Expulsion values obtained for a Padi4^WT^ (**d**) and a Padi4^KO^ (**e**) cell. ROC curve to determine the expulsion threshold above which a cell is deemed to have gone through nuclear expulsion (**f**).

We next used time-lapse imaging to quantify the expansion of H2B-GFP-tagged chromatin in both 4T1 and EO771-LMB cell lines using a custom MATLAB script that allows us to track thousands of single cells at a time. Nuclear expulsion was characterized by a rapid increase in the chromatin area upon treatment with Raptinal (Fig. 1f), in the majority of Padi4^WT^ but not Padi4^KO^ cells (Fig. 1g–i and Extended Data Fig. 2a–c). Approximately 70–90% of Padi4^WT^ cells went through nuclear expulsion, when analyzed by the algorithm (Extended Data Fig. 2d–f). Notably, all are substantially different from the corresponding Padi4^KO^ cells, which had a false-positive rate of 15–20% (Fig. 1j,k). Taken together, these data suggest that dying tumor cells undergo Padi4-dependent nuclear expulsion, resulting in NEPs that consist of a decondensed histone–DNA complex marked with citrullination. This process is likely triggered by calcium-related cell deaths, especially apoptosis.

### Caspases and calcium are necessary for apoptosis-induced nuclear expulsion

**Fig. 2:**
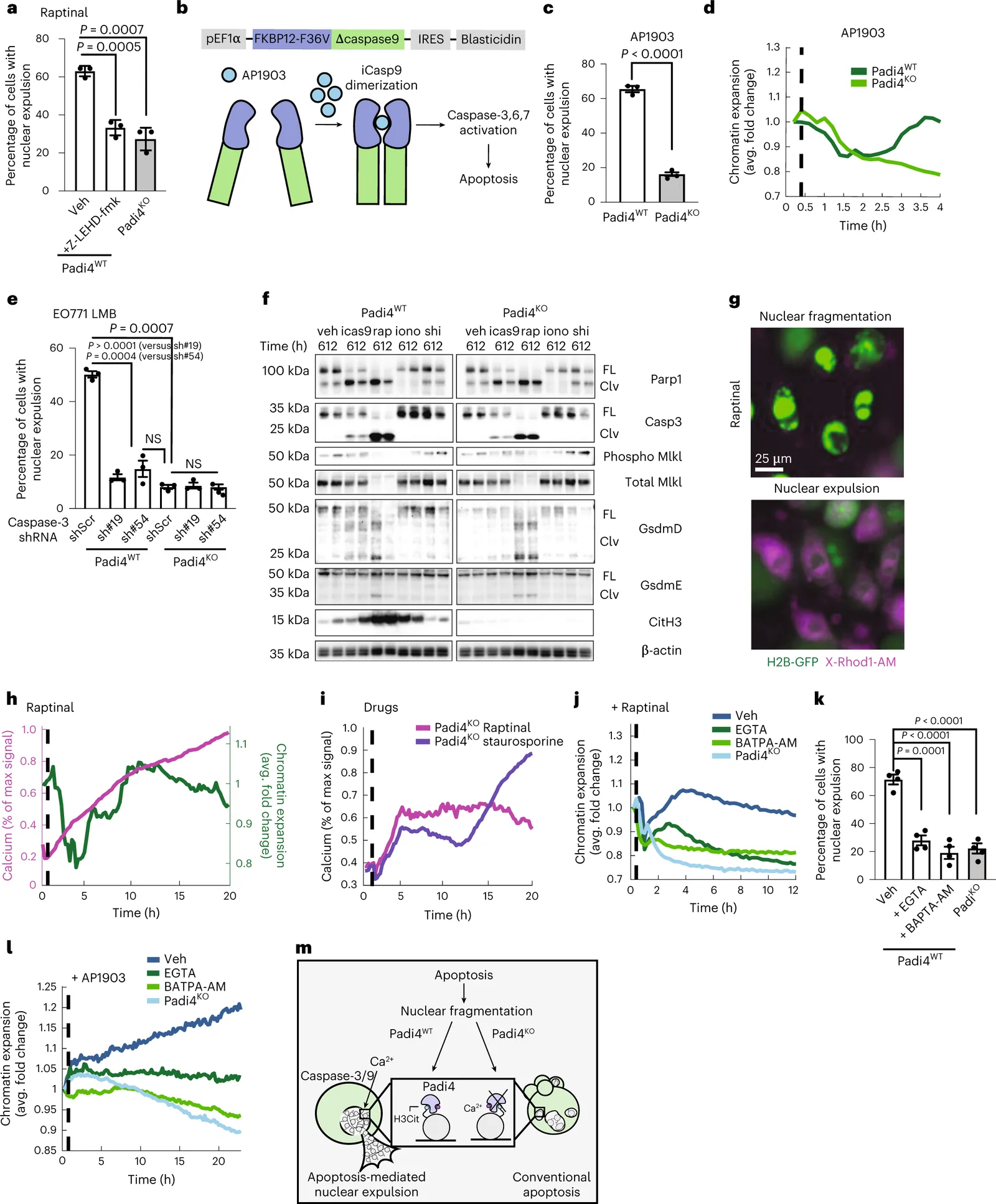
Caspases and calcium are necessary for apoptosis-induced nuclear expulsion. **a**, Percentage of Padi4^WT^ and Padi4^KO^ 4T1 cells treated with Z-LEHD-FMK undergoing nuclear expulsion measured by an expulsion algorithm (*n* = 4,852 of Padi4^WT^cells, *n* = 3,681 of Padi4^WT^ cells with Z-LEHD-FMK and *n* = 6,488 of Padi4^KO^ cells examined over three independent experiments). **b**, Diagram of inducible caspase-9 system. **c**,**d**, Percentage of cells undergoing nuclear expulsion (**c**) and median chromatin expansion (**d**) for Padi4^WT^ and Padi4^KO^ H2B-GFP E0771-LMB cells treated with iCasp9 dimerizing agent AP1903 (*n* = 1,368 of Padi4^WT^cells and *n* = 1,608 of Padi4^KO^ cells examined over three independent experiments). **e**, Percentage of Padi4^WT^/Padi4^KO^ E0771-LMB cells with or without caspase-3 knockdown undergoing nuclear expulsion with treatment of Raptinal (*n* = 773 of Padi4^WT^/caspase3^Scr^ cells, *n* = 1,220 of Padi4^WT^/caspase3^sh#19^ cells, *n* = 1,540 of Padi4^WT^/caspase3^sh#54^ cells, *n* = 1,490 of Padi4^KO^/caspase3^Scr^ cells, *n* = 1,729 of Padi4^WT^/caspase3^sh#19^ cells and *n* = 1,478 of Padi4^KO^/caspase3^sh#54^ cells examined over three independent experiments). **f**, Western blots of cell death markers, Parp1, caspase-3, Mlkl, Gsdm D/E and CitH3 in Padi4^WT^ and Padi4^KO^ cells upon treatment with indicated drugs, AP1903 (icas9), Raptinal (rap), A23187 ionophore (iono) and Shikonin (shi). **g**, Representative live IF images of X-Rhod1-AM as a calcium-signaling indicator in H2B-GFP 4T1 cells treated with Raptinal at 10 h and 16 h. **h**, Dynamics of calcium influx and chromatin expansion during nuclear expulsion in Padi4^WT^ 4T1 cells treated with Raptinal. **i**, Dynamics of calcium influx during nuclear expulsion in Padi4^KO^ 4T1 cells treated with Raptinal (*n* = 5,290 of Padi4^WT^cells and *n* = 5,862 of Padi4^KO^ cells examined over three independent experiments) and staurosporine (*n* = 9,108 of Padi4^WT^cells and *n* = 7,039 of Padi4^KO^ cells examined over three independent experiments). **j**,**k**, Median chromatin expansion (**j**) and percentage of cells undergoing nuclear expulsion (**k**) of EO771-LMB cells when treated with calcium chelators EGTA or BATPA-AM (*n* = 5,290 of Padi4^WT^cells, *n* = 5,862 of Padi4^WT^ cells with EGTA, *n* = 9,108 of Padi4^WT^ cells with BAPTA-AM and *n* = 7,039 of Padi4^KO^ cells examined over three independent experiments). **l**, Median chromatin expansion for thousands of EO771-LMB iCasp9 cells when treated with calcium signaling blockers. **m**, Diagram comparing conventional apoptosis and apoptosis-induced nuclear expulsion. Calcium activates Padi4 to induce histone citrullination which leads to nuclear expulsion. All data are represented as mean ± s.e.m. and *P* values are based on a two-tailed Student’s *t*-test. Western blotting and IF were repeated at least twice and representative data are shown. Source data

**Extended Data Fig. 3:**
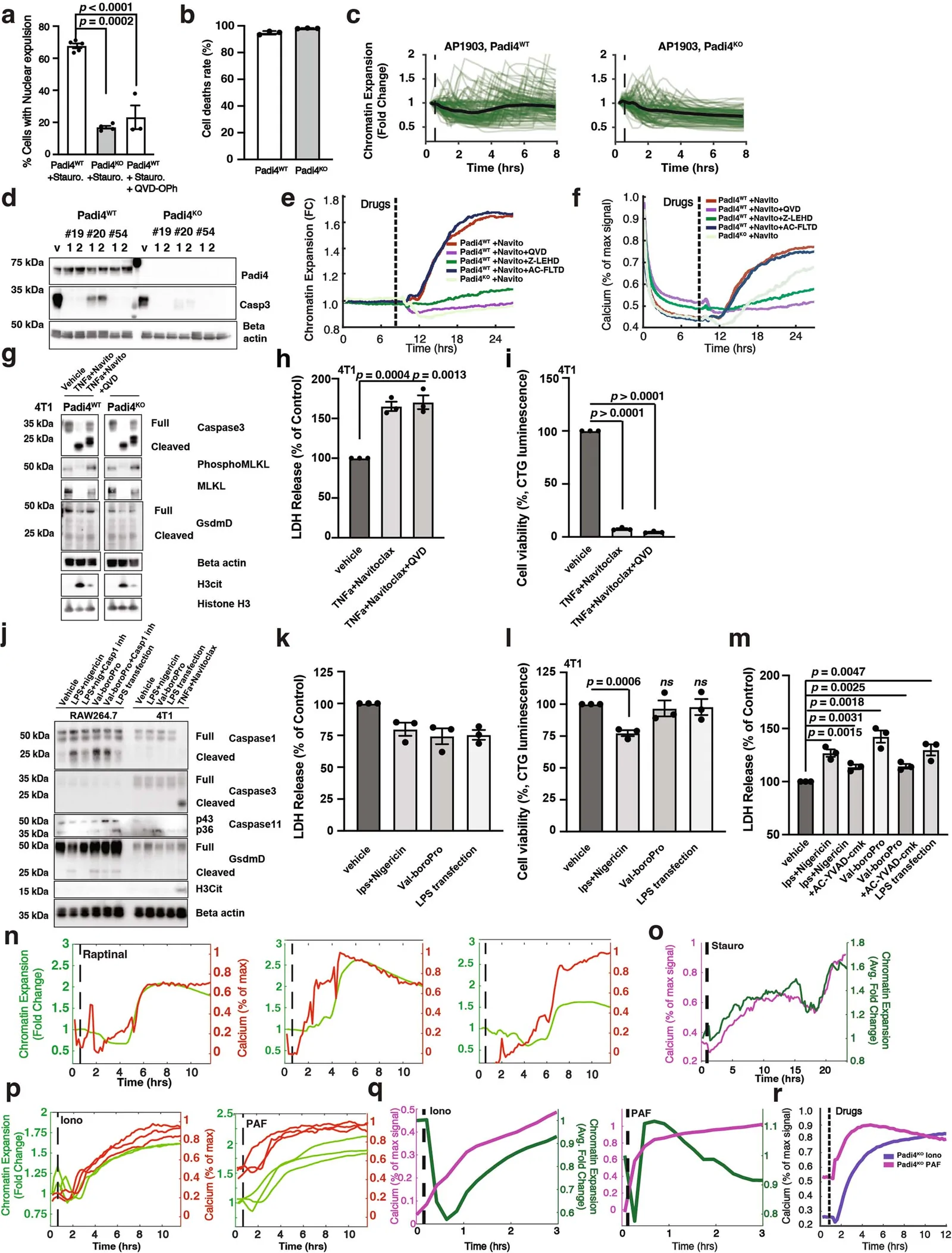
Calcium spike precedes nuclear expulsion during apoptosis-induced nuclear expulsion regardless of Padi4. **a**. Percentage of E0771-LMB cells undergoing nuclear expulsion measured using an expulsion algorithm following treatment of staurosporin with Q-VD-OPh. (*n* = 6304 of Padi4^WT^ cells, *n* = 3987 of Padi4^WT^ cells with Qvd-Oph and *n* = 7550 of Padi4^KO^ cells examined over 3 independent experiments) **b**. Percent cell death in iCaspase9 cells treated with AP1903. n = 3 biologically independent experiments. **c**. Individual traces showing chromatin expansion for 200 Padi4^WT^ and Padi4^KO^ H2B-GFP+ iCaspase9 4T1 cells treated with AP1903. **d**. Western blot of Padi4, caspase-3 (Casp3) in Padi4^WT^ and Padi4^KO^ 4T1 cells. **e - f**. Dynamics of chromatin expansion (**e**) and calcium influx (**f**) during nuclear expulsion in Padi4^WT^ or Padi4^KO^ 4T1 cells treated with navitoclax with caspase inhibitors. **g - i**. Western blot of caspase-3, MLKL, Gasdermin D (Gsdm D) and H3Cit (**g**), LDH release (**h**) and Cell viability (**i**) in Padi4^WT^ and Padi4^KO^ 4T1 cells upon indicated drug treatments. Cells were primed by TNF-a 4 hours before navitoclax treatment. QVD-OPh (QVD) was added 1 hour before navitoclax treatment; *n* = 3 biologically independent experiments. **j-m**. Western blot of caspase 1, −3 and gasdermin D (Gsdm D) and H3Cit in 4T1 and RAW264.7 (**j**), LDH release (**k**) and cell viability (**l**) of 4T1 and LDH release in RAW264.7 (**m**); *n* = 3 biologically independent experiments. **n**. Individual 4T1 cell traces showing chromatin expansion (green) and calcium levels (red) for cells treated with raptinal. **o**. Dynamics of calcium influx and chromatin expansion during nuclear expulsion in Padi4^WT^ 4T1 cells treated with staurosporine. **p**. Individual 4T1 cell traces showing chromatin expansion (green) and calcium level (red) for cells treated with A23187 ionophore (**left**) or PAF (**right**). **q**. Dynamics of calcium influx and chromatin expansion during nuclear expulsion in Padi4^WT^ 4T1 cells treated with A23187 ionophore (**left**), PAF (**right**). **r**. Dynamics of calcium influx during nuclear expulsion in Padi4^KO^ 4T1 cells treated with A23187 ionophore and PAF. All data are represented as mean ± s.e.m., and p values are based on two-tailed student’s t-test. Western blotting and IF were repeated at least twice, and representative data are shown. Source data

Nuclear expulsion is distinctive from the condensation and fragmentation of nuclear contents during apoptosis. To understand the molecular mechanisms, we used caspase inhibitors and calcium chelators to inhibit apoptosis or to block Padi4 function (Supplementary Table 2). 4T1 cells treated with Z-LEHD-FMK, a caspase-9-specific inhibitor or Q-VD-OPh, a pan-caspase inhibitor, showed a decreased number of cells that went through nuclear expulsion (Fig. 2a and Extended Data Fig. 3a). In addition, with an inducible caspase-9 (iCasp9) system, consisting of an FKBP12-F36V dimerizing domain fused with caspase-9 (Fig. 2b), treatment of dimerizing agent AP1903 induced apoptosis in 95% cells through iCasp9 dimerization and effector caspase activation (Extended Data Fig. 3b) and within 3 h, approximately 65% of Padi4^WT^ but not Padi4^KO^ cells began going through nuclear expulsion (Fig. 2c,d and Extended Data Fig. 3c). Furthermore, caspase-3 knockdown reduced nuclear expulsion similar to that of Padi4 knockout in both EO771 and 4T1 cells (Fig. 2e, Extended Data Fig. 3d and Supplementary Videos 6 and 7). We further tested canonical apoptosis inducers BH3-mimetics such as navitoclax and venetoclax (BCL-2 inhibitors), as well as S63845 (MCL-1 inhibitor) and found both robust nuclear bursting and calcium spike in 4T1 tumor cells (Extended Data Fig. 3e,f and Supplementary Video 8). These data demonstrate that effector caspases, such as caspase-9 and 3, are sufficient to induce nuclear expulsion in Padi4-expressing tumor cells.

To understand how nuclear expulsion relates to other forms of cell death, several markers were examined (Gasdermin E (GsdmE) processing for secondary necrosis, Mlkl phosphorylation for necroptosis and GsdmD processing for pyroptosis). Mlkl phosphorylation was not detected in Raptinal- or ionophore-treated cells, excluding necroptosis. Further, GsdmD and GsdmE processing were detected in both Padi4^WT^ and Padi4^KO^ cells treated with Raptinal indicating Padi4-independent induction of secondary necrosis and pyroptosis. Moreover, iCasp9-induced apoptosis did not display GsdmD and GsdmE processing but induced nuclear expulsion discounting the involvement of secondary necrosis and pyroptosis (Fig. 2f). In further investigations, specific induction of necroptosis by tumor necrosis factor (TNF)-α and navitoclax plus the pan-caspase inhibitor QVD-OPh increased pMLKL, decreased cell viability and increased lactate dehydrogenase (LDH) release (Extended Data Fig. 3g–i), but notably, with minimum H3-citrullination (Extended Data Fig. 3g,j). These results confirm that necroptosis is unlikely the main contributor. Unexpectedly, pyroptosis could not be induced in the 4T1 tumor cells by specific pyroptosis inducers (lipopolysaccharide (LPS) plus Nigericin, Val-boroPro or LPS transfection), unlike RAW264.7 cells (Extended Data Fig. 3j–m), indicating that pyroptosis is also unlikely to be a contributor. Considering that cell lysis occurs in Padi4^KO^ cells, but these cells do not go through nuclear expulsion, Padi4-mediated nuclear expulsion is unlikely coupled with general cell lysis. Collectively, we found that apoptosis, but not other forms of programmed cell death, likely triggered the nuclear expulsion.

Padi4, a calcium-dependent enzyme, was necessary for apoptosis-induced nuclear expulsion and both ionophore and PAF trigger calcium-regulated cell death, which suggests that calcium mediates apoptosis-induced nuclear expulsion. Indeed, Raptinal, ionophore and staurosporine clearly increased calcium levels in the nucleus as indicated by X-Rhod-1AM and this occurred before nuclear expulsion (Fig. 2g,h and Extended Data Fig. 3n–q). Notably, an increase in calcium levels was also seen in Padi4^KO^ cells, indicating that calcium levels are not impacted by Padi4 (Fig. 2i and Extended Data Fig. 3r). To further determine whether the calcium spike induced by apoptosis is required for nuclear expulsion, intracellular or extracellular calcium was blocked by pre-treating with BAPTA-AM or EGTA, respectively before Raptinal and AP1903 treatment, which blocked nuclear expulsion (Fig. 2j–l). Collectively, these data suggest that caspases and the subsequent calcium spike are necessary for apoptosis-induced nuclear expulsion (Fig. 2m).

### Padi4-mediated CitH3 and nuclear expulsion in metastatic mouse models

**Fig. 3:**
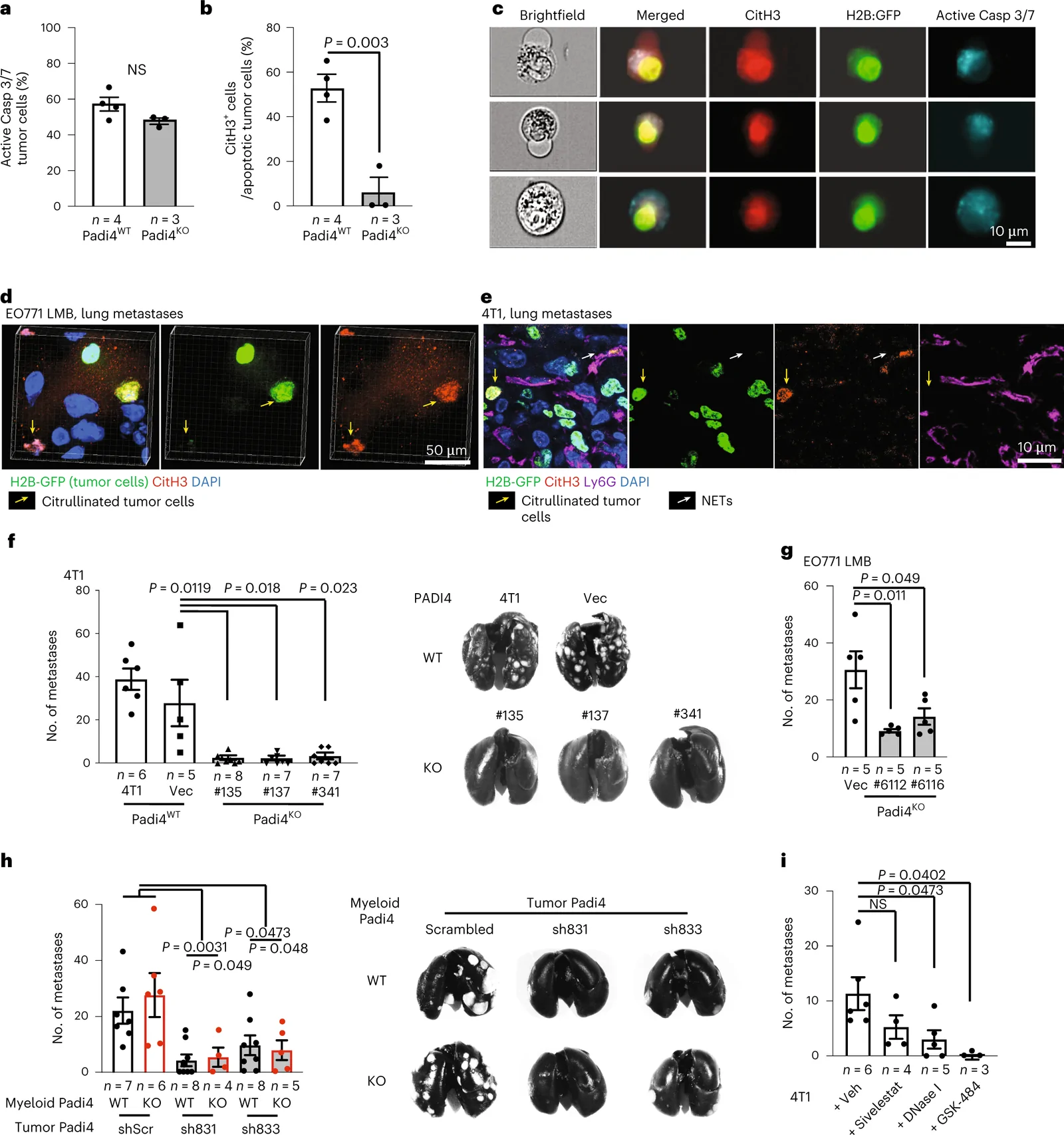
Padi4-mediated CitH3 and nuclear expulsion in mouse models of metastasis. **a**,**b**, Percentage of Padi4^WT^ or Padi4^KO^ H2B-GFP 4T1 with active caspase-3/7 (**a**) and with CitH3 positivity among cells showing active caspase-3/7 (**b**). Cells were collected from the lungs of the mice 24 h after TVI, *n* = mice. **c**, Imaging flow cytometry of Padi4^WT^ 4T1 cells that have gone through apoptosis-induced nuclear expulsion in vivo. Cells were stained for CitH3 and active caspase-3/7. **d**,**e**, Representative IF images of CitH3, Ly6G and H2B-GFP in lung metastases from mice bearing EO771-LMB (**d**) or 4T1 tumors. Yellow arrows, NEPs; white arrows, NETs. **f**,**g**, Number of lung metastases in mice that received mammary fat pad (MFP) injection of Padi4^WT^ or Padi4^KO^ 4T1 cells (**f**) and EO771-LMB cells (**g**). Representative images from Indian ink staining (**f** right); 4T1, non-infected control; Vec, empty vector control; #135, #137, #341 (4T1) and #6112, #6116 (EO771) represent each Padi4^KO^ clone. *n* = mice. **h**, Number of lung metastases from myeloid-specific Padi4^WT^ or Padi4^KO^ mice that bear EO771 tumors with Padi4 knockdown or scrambled short hairpin (sh)RNA (left), representative images from Indian ink staining (right), *n* = mice. **i**, Number of metastases from mice bearing 4T1 tumors treated with sivelestat, DNase I and GSK-484, *n* = mice. All data are represented as mean ± s.e.m. and *P* values are based on two-tailed Student’s *t*-test. Source data

**Extended Data Fig. 4:**
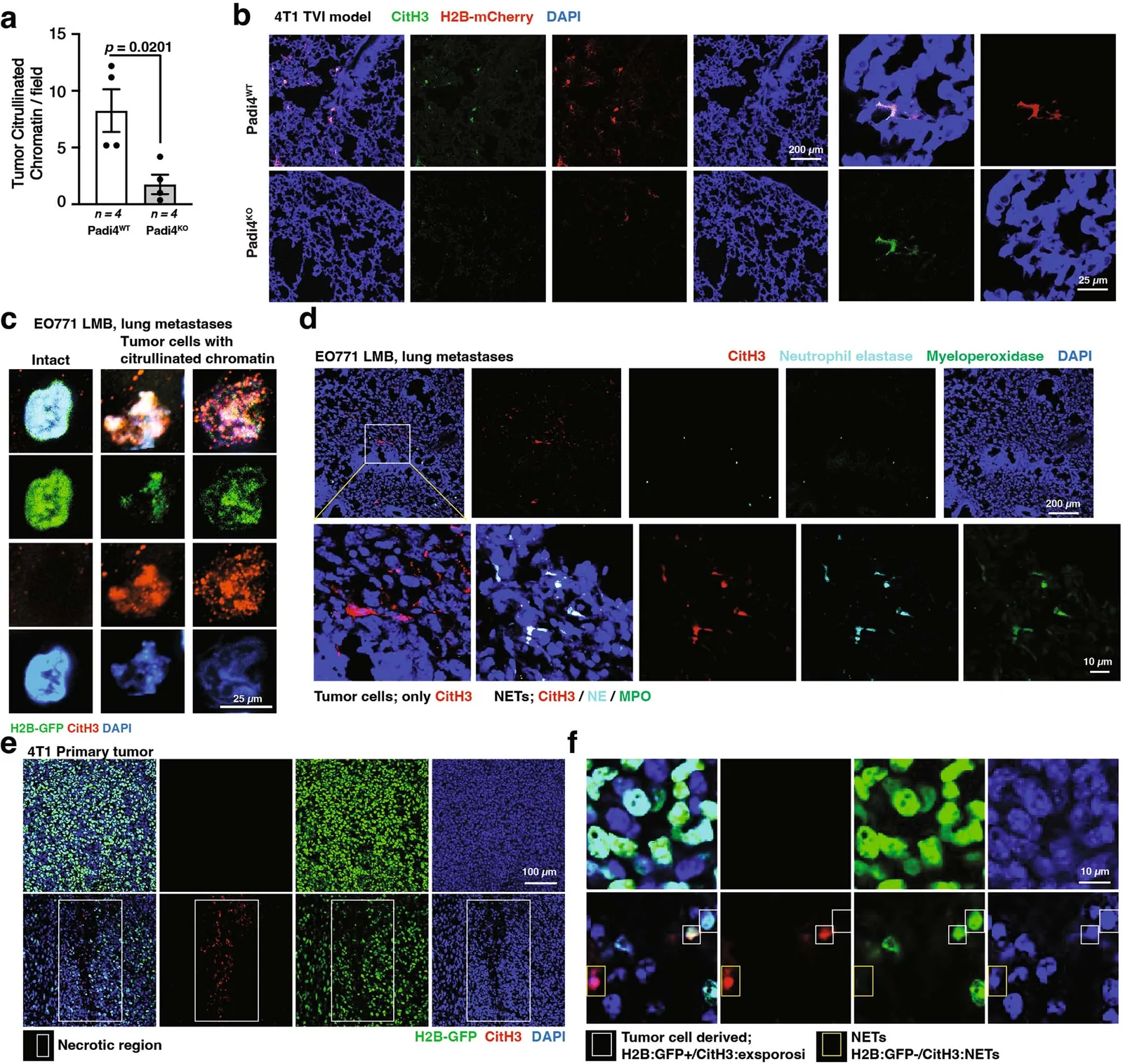
Tumor cell NEPs are found in mouse models of metastasis. **a**. Quantification of overlapping CitH3 and H2B-mCherry in the lungs of mice that received TVI of Padi4^WT^ or Padi4^KO^ 4T1 cells. *n* = mice. Error bars represent s.e.m., and p value is based on two-tailed student’s t-test. (*) P<0.05. **b**. CitH3 and H2B-mCherry immunostaining of NEPs in the lungs of mice that received TVI injection of 4T1 cells for 4 days. Right 4 panels are higher magnified images. Red: Tumor cells (H2B-mCherry), Green: citH3, Blue: DAPI. **c**. Super-resolution images showing overlap of CitH3 and H2B-GFP staining of metastatic tumor cells of EO771-LMB 28 days after MFP injection. Green: H2B-GFP only in tumor cells; Red: CitH3; Blue: DAPI. **d**. Representative IF images of tumor NEPs or NETs in lung metastases from mice bearing EO771-LMB tumors 35 days after MFP injection. Bottom panels are higher magnified images of citrullinated chromatin in tumor cells (**most left**) or NETs (**right 5 panels**): Cyan: Neutrophil elastase (NE), Green: Myeloperoxidase (MPO), Red: citH3, Blue: DAPI. **e**. IF images for CitH3 in tumor cells as well as NETs in necrotic region of primary tumor from mice bearing 4T1 tumors. Box represents necrotic region. Green: H2B-GFP only in tumor cells; Red: CitH3; Blue: DAPI. **f**. Higher magnification from Extended Data Fig. 4e. Top panels: representative images of intact tumor cells; Lower panels: tumor cells undergoing nuclear expulsion. Yellow box: NETs, White boxes: Tumor cells. Green: H2B-GFP (tumor cell only), Red: CitH3, Blue: DAPI. Source data

A majority of tumor cells die in circulation or distant sites during the metastatic process. We thus performed a tail vein injection (TVI) of 4T1 cells and checked caspase-3/7 activation and CitH3. Over 50% of tumor cells were apoptotic after 24 h with no difference between Padi4^WT^ and Padi4^KO^ cells (Fig. 3a). Among apoptotic tumor cells, more than 50% of Padi4^WT^ cells were CitH3 positive versus ~5% in Padi4^KO^ cells (Fig. 3b). There was clearly diffused CitH3 and activated caspase-3/7 in the Padi4^WT^ tumor cells (Fig. 3c), with little CitH3 in the Padi4^KO^ tumor cells (Extended Data Fig. 4a,b). CitH3 was also found in lung metastatic nodules in mouse models of spontaneous metastasis for both EO771-LMB and 4T1 tumors (Fig. 3d,e) and the CitH3 was diffuse, fiber-like and surrounded other tumor cells (Extended Data Fig. 4c,d). Notably, CitH3 was also observed in primary tumor tissues in necrotic regions where NETs were also found (Extended Data Fig. 4e,f).

**Extended Data Fig. 5:**
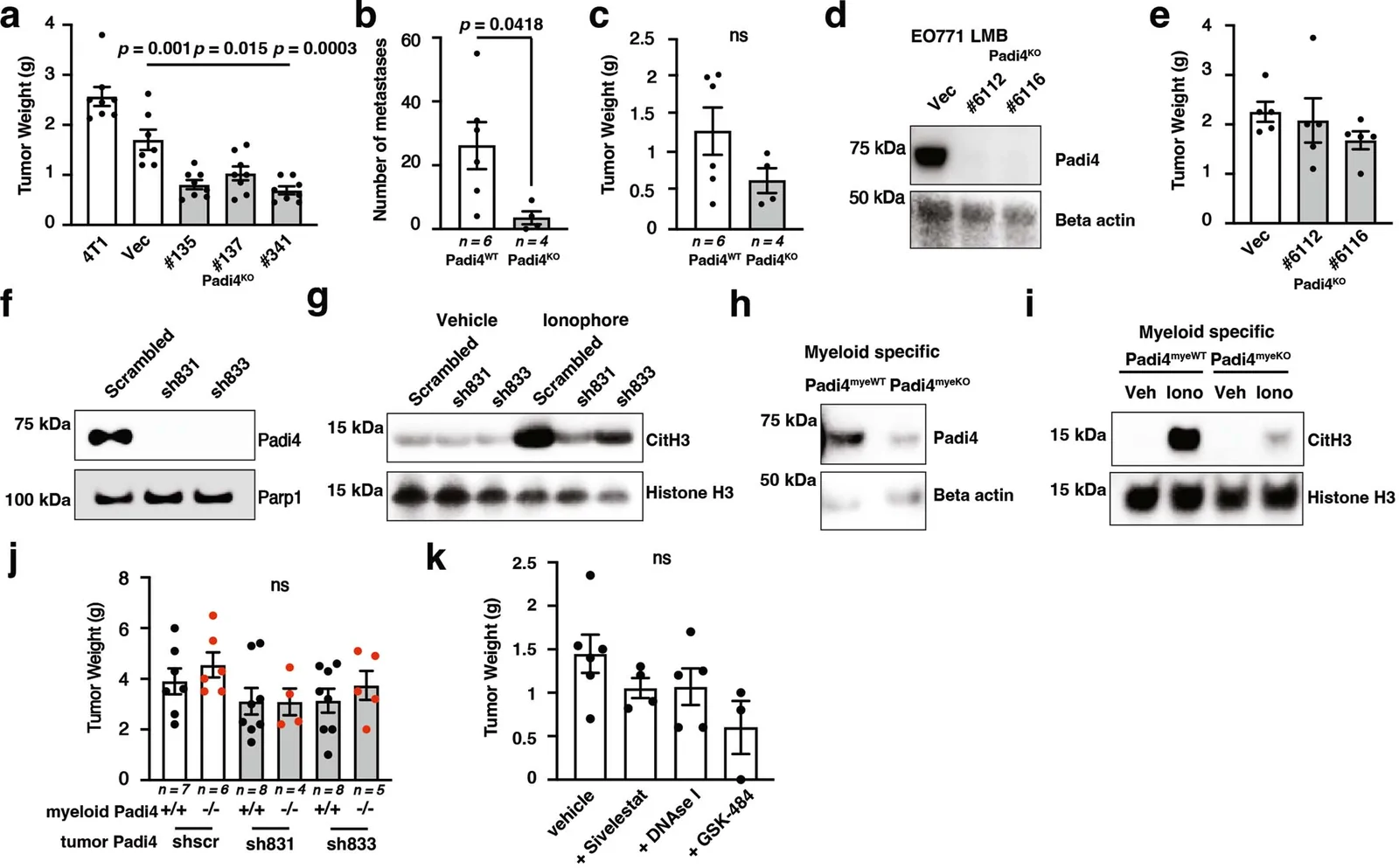
Tumor cell NEPs enhances metastasis in a manner independent from NETosis. **a**. Tumor weight from parental (*n* = 6), empty vector (*n* = 5), and Padi4^KO^ 4T1 (#135, *n* = 8; #137, *n* = 7; #341, *n* = 7) mouse models. *n* = tumors. **b** - **c**. Number of lung metastases from size-matched tumors (**b**) and tumor weight (**c**) from 4T1 empty vector versus Padi4^KO^ cells. *n* = mice (b) or tumors (c). **d**. Padi4 Western blot of EO771-LMB cells for validating Padi4 knockout. **e**. Tumor weight from empty vector (*n* = 5) and Padi4^KO^ EO771 (#6112, *n* = 5; #6116, *n* = 5) mouse model. *n* = tumors **f** - **g**. Padi4 (**f**) and CitH3 (**g**) Western of EO771-LMB cells with Padi4 knockdown and scrambled shRNA control. **h** - **i**. Western blots of Padi4 (**h**) and CitH3 (**i**) in Ly6G+ neutrophils from Padi4^myeWT^ or Padi4^myeKO^ mice to confirm Padi4 knockout in myeloid compartment. **j**. Tumor weight from Padi4^myeKO^ and wildtype mice orthotopically injected with scrambled shRNA control or Padi4 knockdown EO771-LMB cells. *n* = tumors. **k**. Tumor weight from 4T1 tumor-bearing mice treated with vehicle (*n* = 6), Sivelestat (*n* = 4), DNaseI (*n* = 5) and GSK-484 (*n* = 3). *n* = tumors. All data are represented as mean ± s.e.m., and p values are based on two-tailed student’s t-test. Source data

Next, we sought to determine whether Padi4 in tumor cells and the cognate citrullinated chromatin enhanced tumor metastasis. First, Padi4 knockout produced fewer metastatic nodules in orthotopic tumor models of 4T1, even when 4T1 primary tumor size was matched (Fig. 3f and Extended Data Fig. 5a–c) and EO771-LMB, with no difference in EO771 primary tumor size (Fig. 3g and Extended Data Fig. 5d,e). This suggests a more pronounced effect of Padi4 on metastasis than primary tumors. Second, a myeloid-specific Padi4 knockout mouse model (Padi4^myeKO^) was established to compare Padi4’s effect on metastasis between tumor cells and the myeloid compartment, as previous studies imply neutrophil-produced and Padi4-dependent NETs in metastasis^14–16^. Consistent with findings presented earlier, Padi4 knockdown in EO771-LMB cells led to fewer metastases, with no effect on primary tumor growth (Fig. 3h and Extended Data Fig. 5f–j). Notably, myeloid-specific Padi4 deletion did not have any effect on metastasis or on primary tumor growth, discounting the involvement of Padi4-mediated NETosis (Fig. 3h and Extended Data Fig. 5h–j). We also used sivelestat, a neutrophil elastase (NE) inhibitor, to block NET formation and found it had only a marginal effect on metastasis (Fig. 3i). In addition, GSK-484, a Padi4 inhibitor, as well as DNase I, an enzyme that degrades DNA, clearly decreased lung metastasis, but not primary tumor size, in the 4T1 model (Fig. 3i and Extended Data Fig. 5k). Together, these findings demonstrate that cancer-derived citrullinated chromatin occurs in vivo and likely enhances lung metastasis through mechanisms independent from Padi4-induced NETs.

### Nuclear expulsion mediates the metastatic outgrowth in the lung

**Fig. 4:**
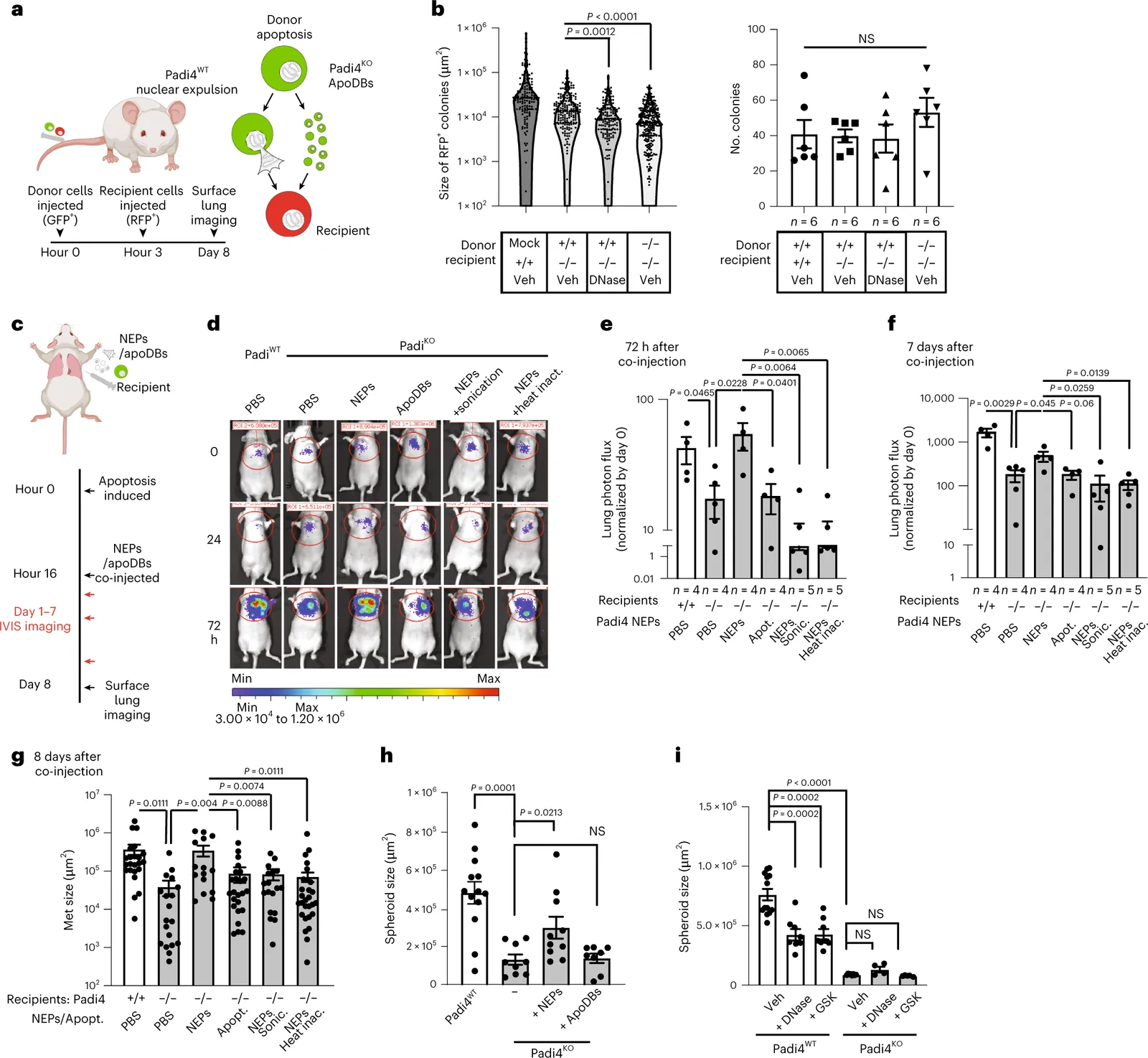
Nuclear expulsion promotes metastatic outgrowth. **a**, Schematic experimental design for non-autonomous effects of Padi4 on lung metastasis. **b**, Size (left, *n* = metastatic colonies) and number (right, *n* = mice) of mCherry^+^ metastatic colonies from Padi4^WT^ or Padi4^KO^ H2B-mCherry 4T1 cells (recipient) co-injected with Padi4^WT^ or Padi4^KO^ (donor) under indicated conditions. The thin dotted line on the violin plot represents the upper and lower quartiles and the thick dashed line represent the median. Padi4^WT^ + Padi4^WT^-H2B:mCherry, *n* = 132; Padi4^WT^ + Padi4^KO^-H2B:mCherry, *n* = 174; Padi4^WT^ + Padi4^KO^-H2B:mCherry + DNase I, *n* = 145; Padi4^KO^-H2B:mCherry only, *n* = 269. **c**, Schematic experimental design for NEP effect on lung metastasis using intrathoracic injection. **d**–**g**, Bioluminescent imaging of metastatic burden in the lungs. Bioluminescent images (**d**), quantitative data (**e**,**f**, *n* = mice) or size of surface lung metastatic nodules (**g**, *n* = metastatic colonies). 4T1, *n* = 23; 4T1 Padi4^KO^, *n* = 19; 4T1 Padi4^KO^ + NEPs, *n* = 15; 4T1 Padi4^KO^ + apoDBs, *n* = 28; Padi4^KO^ + sonic-NEPs, *n* = 23; Padi4^KO^ + heat-NEPs, *n* = 18; from mice that received intrathoracic co-injection of Padi4^WT^ or Padi4^KO^ 4T1 cells with NEPs, apoDBs or PBS at indicated times. Sonic., sonicated NEPs; Heat inac., heat-inactivated NEPs. **h**,**i**, Size of the spheroid from Padi4^WT^ or Padi4^KO^ 4T1 cells co-cultured with NEPs or apoDBs (**h**), as well as treated with DNase or GSK-484 (**i**), *n* = spheres. 4T1, *n* = 13; 4T1 Padi4^KO^, *n* = 9; 4T1 Padi4^KO ^+ NEPs, *n* = 10; 4T1 Padi4^KO^ + apoDBs, *n* = 8; **i**: 4T1, *n* = 13; 4T1 + DNase I, *n* = 8; 4T1 + GSK-484, *n* = 8; 4T1 Padi4^KO^, *n* = 8; 4T1 Padi4^KO^ + DNase I, *n* = 4; 4T1 Padi4^KO^ + GSK-484, *n* = 5 (**h**). All data are represented as mean ± s.e.m. and *P* values are based on a two-tailed Student’s *t*-test. Source data

**Extended Data Fig. 6:**
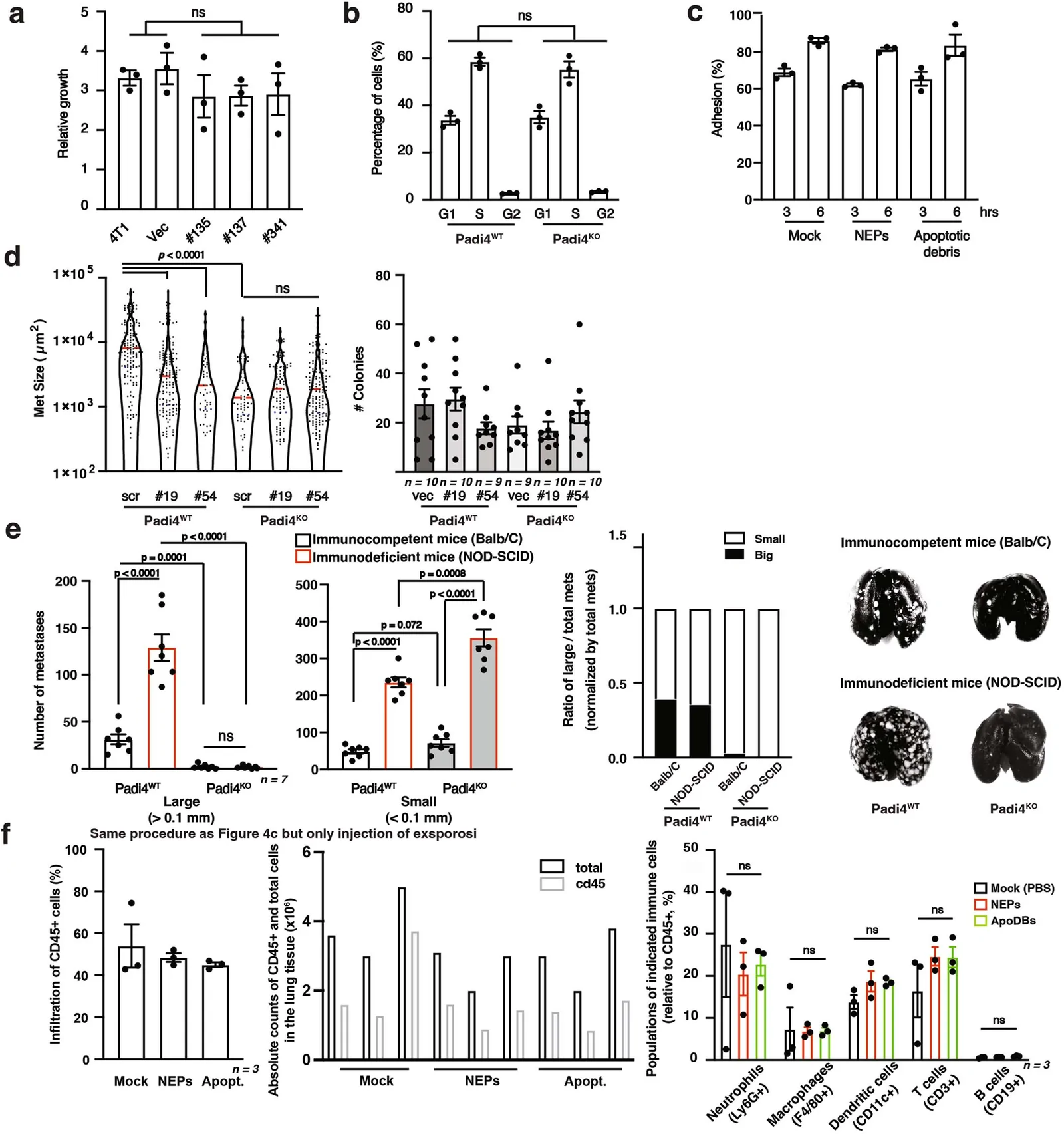
No significant effect of Padi4 or NEPs on tumor cell proliferation, cell cycle, cell adhesion as well as immune response. **a**. MTT assay for relative growth of Padi4^WT^ and Padi4^KO^ 4T1 cells. Day 3 signals were normalized by day 0. *n* = 3 biologically independent experiments. **b**. Distribution of cell cycle phase in Padi4^WT^ and Padi4^KO^ 4T1 cells. *n* = 3 biologically independent experiments. **c**. Adhesion assay of Padi4^KO^ 4T1 cells. NEPs or ApoDBs were pre-coated 4 hours before tumor cells seeded. *n* = 3 biologically independent experiments. **d**. Size (**left**) and number (**right**) of GFP+ metastatic colonies from empty vector, two caspase3 knockdown #19 or #54 in both Padi4^WT^ and Padi4^KO^ H2B-GFP 4T1 cells. The thin blue dotted line on the violin plot represents the upper and lower quartiles and the thick red dashed line represents the median. *n* = individual GFP positive colonies (4T1-Scr, *n* = 140; 4T1-#19, *n* = 156; 4T1-#54, *n* = 52; 4T1 Padi4^KO^-Scr *n* = 64; 4T1 Padi4^KO^-#19, *n* = 84; 4T1 Padi4^KO^-#54, *n* = 156; left) or mice (right). **e**. Number of metastases of Padi4^WT^ and Padi4^KO^ cells in immune competent mice (black) and immune deficient mice (red) (**left two panels**). Ratio of big mets divided by total mets (**the third panel**) and representative images of Indian ink staining in the right panels. *n* = mice. **f**. Flow cytometry of immune cells in the lungs after the injection of NEPs or apoDBs. **Left two panels** are percentage and absolute counts of CD45^+^ population in lung tissue, **the third panel** is immune cell subsets within CD45^+^ population. *n* = mice. All data are represented as mean ± s.e.m., and p values are based on two-tailed student’s t-test. Source data

Our data led us to hypothesize that apoptotic tumor cells produce NEPs that could be utilized by the surviving tumor cells for metastatic colonization. We first noticed that there were no differences between Padi4^WT^ and Padi4^KO^ cells in general growth, cell cycle and cell adhesion (Extended Data Fig. 6a–c), indicating a likely non-cell-autonomous effect. We thus performed an intravenous co-injection of 4T1 GFP^+^ donor cells (Padi4^WT^, producing NEPs) and 4T1 mCherry^+^ recipient cells (Padi4^KO^, not producing NEPs) (Fig. 4a). Of note, the size of mCherry^+^ recipient Padi4^KO^ metastatic nodules was increased when co-injected with Padi4^WT^ compared to Padi4^KO^ cells injected alone (Fig. 4b, left), with no difference in the nodule number (Fig. 4b, right). A single DNase treatment following injection reduced the size of Padi4^KO^ nodules but not the number (Fig. 4b). Caspase-3 knockdown impaired nuclear expulsion (Fig. 2f) and decreased metastasis size (Extended Data Fig. 6d) without affecting metastasis number, phenocopying that of the Padi4 knockout. We next performed intrathoracic co-injection of Padi4^KO^ recipient cells with isolated NEPs or apoptotic debris (apoDBs) as a control. Tumor lesions were monitored for early metastatic outgrowth (Fig. 4c). As expected, co-injection with NEPs enhanced Padi4^KO^ metastatic outgrowth at 72 h and showed an increased metastases size on day 8, which was not observed when co-injected with apoDBs (Fig. 4d–g). Notably, sonicating or heat-inactivating NEPs, which disrupts the DNA structure and protein activity, decreased metastatic nodule size (Fig. 4d–g). Of note, NEP-mediated outgrowth was decreased, whereas Padi4^WT^ cells grew rapidly at day 7 (Fig. 4f), likely resulting from a continuous NEP production by the Padi4^WT^ cells. These results are consistent with spheroid culture in which co-culturing Padi4^KO^ cells with NEPs but not apoDBs increased the Padi4^KO^ spheroid size (Fig. 4h). Consistently, DNase I and GSK-484 decreased the size of Padi4^WT^ but not Padi4^KO^ spheroids (Fig. 4i).

To investigate the possibility that NEPs increase metastatic outgrowth by acting on the host immune system, Padi4^WT^ and Padi4^KO^ 4T1 cells were injected into immune-competent Balb/c and immune-deficient NOD-SCID mice. Padi4^KO^ cells failed to produce large metastases (>0.1 mm) in both Balb/c and NOD-SCID mice (Extended Data Fig. 6e, left). While immunodeficient mice had more metastasis in general, there was no difference in the ratio of large metastases to the total number of metastases (Extended Data Fig. 6e). These data indicate that the immune system affects the overall metastatic colonization, but Padi4 is critical for the outgrowth of small nodules. Furthermore, immune cell profiling of lungs from Balb/c mice receiving an intrathoracic injection of either NEPs or apoDBs showed no difference in infiltration of CD45^+^ immune cells (Extended Data Fig. 6f, left) as well as no changes in neutrophils, macrophages, dendritic cells and T-cell and B-cell subsets among CD45^+^ cells (Extended Data Fig. 6f, right). Together, these findings suggest that the chromatin in NEPs mediates tumor outgrowth and the host immune system may not play a major role in the process.

### Chromatin-bound S100a4 is essential for NEP-mediated metastatic outgrowth

**Fig. 5:**
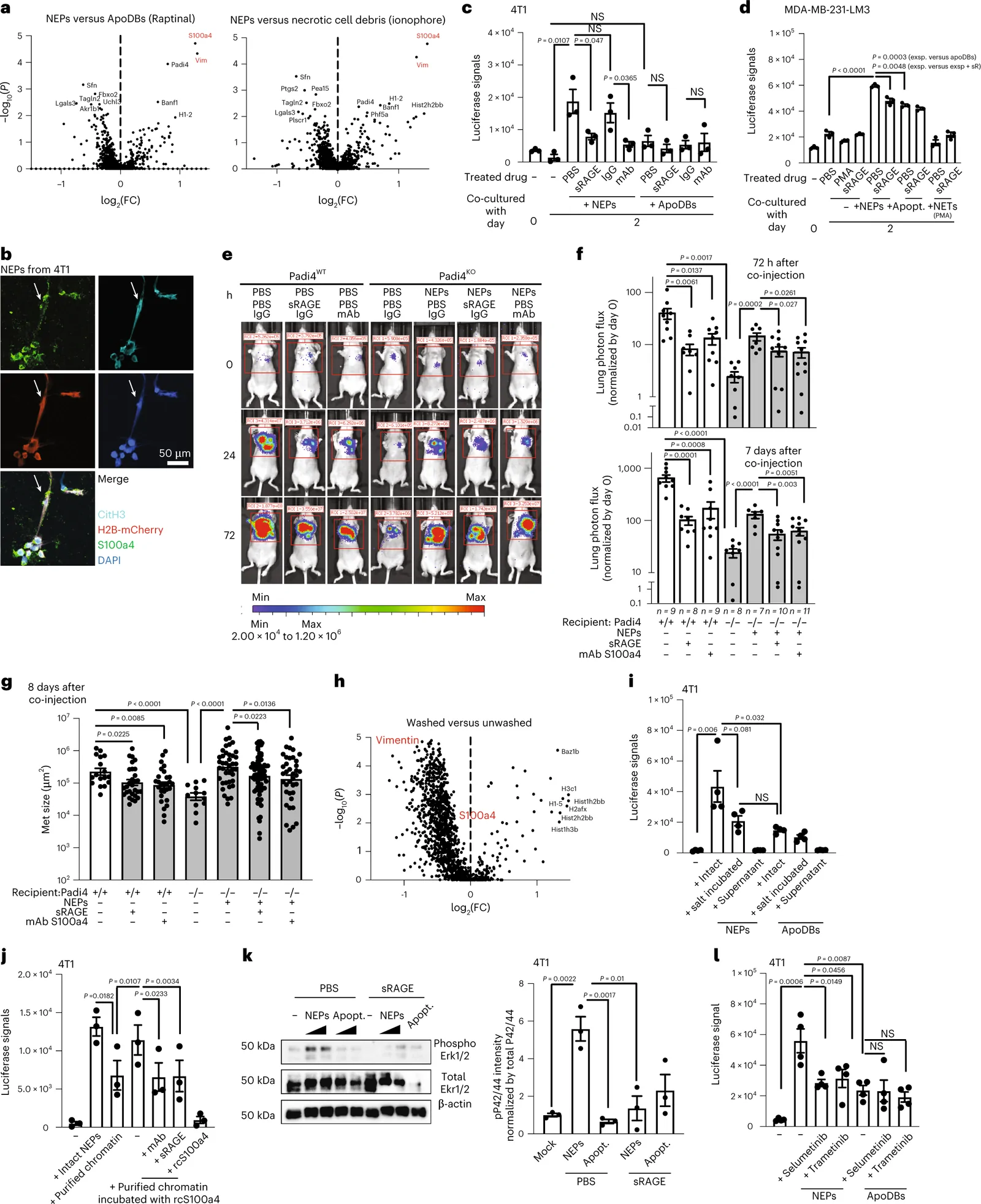
Chromatin-bound S100a4 in NEPs mediates RAGE activation in tumor cells leading to metastatic outgrowth. **a**, Volcano plots of proteomics analysis −log_10_(*P*) versus log_2_ expression levels comparing NEPs to apoDBs or necrotic cell debris upon Raptinal (left) or A23187 ionophore (right) treatment. Mass spectrometry was performed using biologically independent samples (*n* = 3). FC, fold change. **b**, IF images for S100a4 and CitH3 in NEPs. cyan, CitH3; red, H2B-mCherry; green, S100a4; blue, DAPI. White arrows, S100a4 co-localized NEPs. **c**, Luciferase signals of tumor cell growth from co-culture of Padi4^KO^ 4T1 cells with NEPs or apoDBs upon treatment of a sRAGE peptide (decoy RAGE receptor) or S100a4 neutralizing antibody (monoclonal antibody (mAb)), *n* = 3 biologically independent experiments. **d**, Luciferase signals of tumor cell growth from co-culture of MDA-MB-231-LM3 cells with iCasp9-generated NEPs or NETs upon treatment with sRAGE. PMA was used to induce NETs in neutrophils, *n* = 3 biologically independent experiments. PMA, phorbol 12-myristate 13-acetate. **e**–**g**, Bioluminescent imaging for lung metastatic burden. Representative images (**e**) or quantitative data (**f**, *n* = mice) and lung surface nodule size (**g**, *n* = metastatic colonies) from mice that received intrathoracic co-injection of Padi4^WT^ or Padi4^KO^ 4T1 cells with NEPs or apoDBs at indicated time. 4T1, *n* = 17; 4T1+sRAGE, *n* = 28; 4T1+neuAb, *n* = 28; 4T1 Padi4^KO^, *n* = 12; Padi4^KO^+NEPs, *n* = 40; Padi4^KO^+NEPs+sRAGE, *n* = 75; Padi4^KO^+NEPs+neuAb, *n* = 37 (**g**). **h**, Volcano plot of proteomics analysis −log_10_(*P*) versus log_2_ expression levels comparing washed NEPs (NEP-bound proteins) relative to unwashed NEPs (soluble and bound). Mass spectrometry was performed using biologically independent samples (*n* = 3). **i**,**j**, Luciferase signals of tumor cell growth from co-cultured Padi4^KO^ 4T1 cells with salt-incubated NEPs, apoDBs or their supernatants (**i**), as well as with purified chromatin or recombinant S100a4 re-bound to chromatin upon treatment of sRAGE or mAb (**j**), *n* = 3 biologically independent experiments. **k**, Western blot of Erk1/2 with Padi4^KO^ 4T1 cells co-cultured with NEPs or apoDBs (Apopt.) upon a treatment with sRAGE or PBS. Quantitative ratio of phosphor Erk1/2 to total Erk1/2 (right). **l**, Luciferase signal of tumor cell growth from co-cultured Padi4^KO^ 4T1 cells with NEPs or apoDBs upon treatment with selumetinib or trametinib, p42/44 MAPK inhibitors, *n* = 3 biologically independent experiments. All data are represented as mean ± s.e.m. and *P* values are based on a two-tailed Student’s *t*-test. Western blotting and IF were repeated at least twice and representative data are shown. Source data

**Extended Data Fig. 7:**
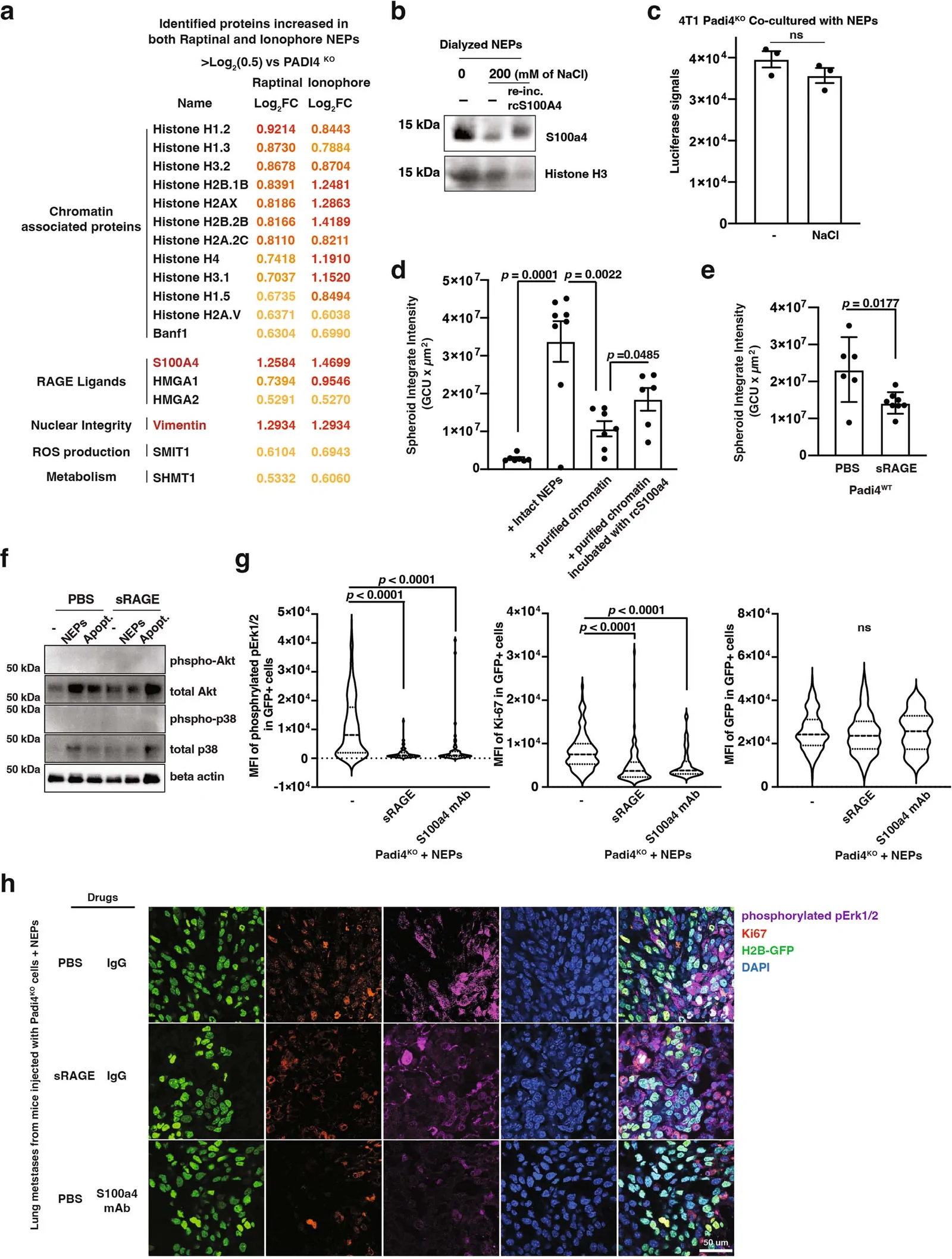
Chromatin bound S100a4 in NEPs mediates RAGE activation in tumor cells leading to metastatic outgrowth. **a**. List of proteins identified as NEPs abundant components from proteomic analysis. **b**. Western blots of S100a4 or histone H3 from dialyzed NEPs or NEPs re-incubated with S100a4. **c**. Luciferase signals of co-cultured Padi4^KO^ 4T1 cells with NEPs upon treatment of NaCl. Same amount of salt as in Fig. 5i was added directly into culture media. *n* = 3 biologically independent experiments. **d**. Spheroid culture of Padi4^KO^ 4T1 cells treated with dialyzed NEPs or S100a4 re-bound NEPs. *n* = spheres. Padi4^WT^, *n* = 7; Padi4^KO^+NEPs, *n* = 8; Padi4^KO^+purified chromatin, *n* = 7; Padi4^KO^+purified chromatin+rcs100a4. **e**. Spheroid culture of Padi4^WT^ 4T1 cells treated with sRAGE. *n* = spheres. Padi4^WT^ with vehicle (*n* = 6) or sRAGE (*n* = 8). **f**. Western blots of indicated signaling pathways with Padi4^KO^ 4T1 cells co-cultured with NEPs or apoDBs upon a treatment with sRAGE. **g**. Mean Fluorescence Intensity (MFI) of Erk1/2 (**left**), Ki-67 (**middle**) and GFP (**right**) in lung metastases from mice that were treated with vehicle (*n* = 290), sRAGE (*n* = 100) or S100a4 mAb (*n* = 50) used in Fig. 5e. *n* = individual H2B:GFP labeled tumor cells. The thin dotted line on the violin plot represents the upper and lower quartiles and the thick dashed line represents the median. **h**. Representative IF images of Fig. 7g. Purple: phosphorylated Erk1/2, Red: Ki-67, Green: H2B-GFP (Tumor cell only), Blue: DAPI. All data are represented as mean ± s.e.m., and p values are based on two-tailed student’s t-test. Source data

To identify the molecular mediators by which NEPs promote tumor metastatic outgrowth, we performed tandem mass spectrometry on NEPs from 4T1 Padi4^WT^ cells and apoDBs from Padi4^KO^ cells induced by Raptinal and ionophore. This resulted in 1,215 proteins that had two or more peptide-spectrum matches. There was a clear enrichment in histone variants and chromatin-associated proteins in NEPs when compared to apoDBs (Fig. 5a and Extended Data Fig. 7a). S100a4 and vimentin were the top differentially increased proteins for both Raptinal- and ionophore-induced NEPs (Fig. 5a and Extended Data Fig. 7a). Other RAGE ligands, such as the HMG family, were also abundant suggesting the importance of the RAGE pathway (Extended Data Fig. 7a). In accordance with the mass spectrometry results, S100a4 clearly colocalized with chromatin and CitH3 (Fig. 5b).

S100a4 activates the RAGE pathway, which is known to enhance tumor growth and metastasis^23–26^. In NEP-mediated metastatic outgrowth, S100a4 neutralization by monoclonal antibody S100a4 or inhibition of S100a4-RAGE interaction by sRAGE, a soluble decoy receptor, substantially diminished the tumor cell growth in a co-culture of Padi4^KO^ cells with NEPs (Fig. 5c). Furthermore, sRAGE also diminished the growth induced by NEPs generated from MDA-MB-231-LM3 cells, suggesting that this pathway is shared in human cells (Fig. 5d). To investigate the effect in vivo, a mixture of tumor cells and NEPs were injected intrathoracically into the lungs followed by injection of monoclonal antibodies S100a4 or sRAGE, which decreased the metastatic burden (Fig. 5e,f) and the size of the metastatic nodules (Fig. 5g). These results suggest a dependency of the RAGE pathway in NEP-mediated metastatic outgrowth.

We propose that chromatin-bound proteins are a key player in NEPs as an intact DNA structure was required for its effect (Figs. 3i and 4b). To determine which proteins were bound to chromatin, we performed tandem mass spectrometry and compared unwashed versus washed ionophore-induced NEPs. S100a4 was present in roughly equal proportions in unwashed and washed conditions, suggesting that S100a4 is loosely bound to chromatin (Fig. 5h). On the other hand, vimentin was higher in the unwashed condition, suggesting it is soluble and it is unlikely to be involved in mediating metastatic outgrowth (Fig. 5h). To further investigate the requirement of chromatin-bound S100a4 in NEPs, we dissociated non-histone proteins such as S100a4 from NEPs and remained core histones by salt fractionation (Extended Data Fig. 7b)^27^. When added to the co-culture, salt-incubated NEPs as well as the supernatant from salt-incubated NEPs had a greatly diminished effect on growth (Fig. 5i and Extended Data Fig. 7c), which was not observed in cells co-cultured with apoDBs (Fig. 5i). Furthermore, re-attachment of recombinant S100a4 (rcS100a4) to salt-purified chromatin rescued the effect of NEPs, which was then inhibited by sRAGE and monoclonal antibody S100a4 in both co-culture and a spheroid culture system (Fig. 5j and Extended Data Fig. 7d,e). Consistently, tumor cells co-cultured with NEPs showed a specific increase in pErk1/2, which was inhibited by sRAGE (Fig. 5k and Extended Data Fig. 7f) and tumor cell growth was diminished by the MAPK inhibitors selumetinib or trametinib (Fig. 5l). In addition, lung metastasis lesions showed a clear increase in pErk1/2 and Ki-67 in tumor cells but were inhibited by monoclonal antibody S100a4 or sRAGE (Extended Data Fig. 7g,h). Thus, chromatin-bound S100a4 in NEPs is critical in facilitating metastatic outgrowth via RAGE-mediated Erk1/2-MAPK signaling.

### Inflammatory mediators in the lung induce Padi4 expression

**Fig. 6:**
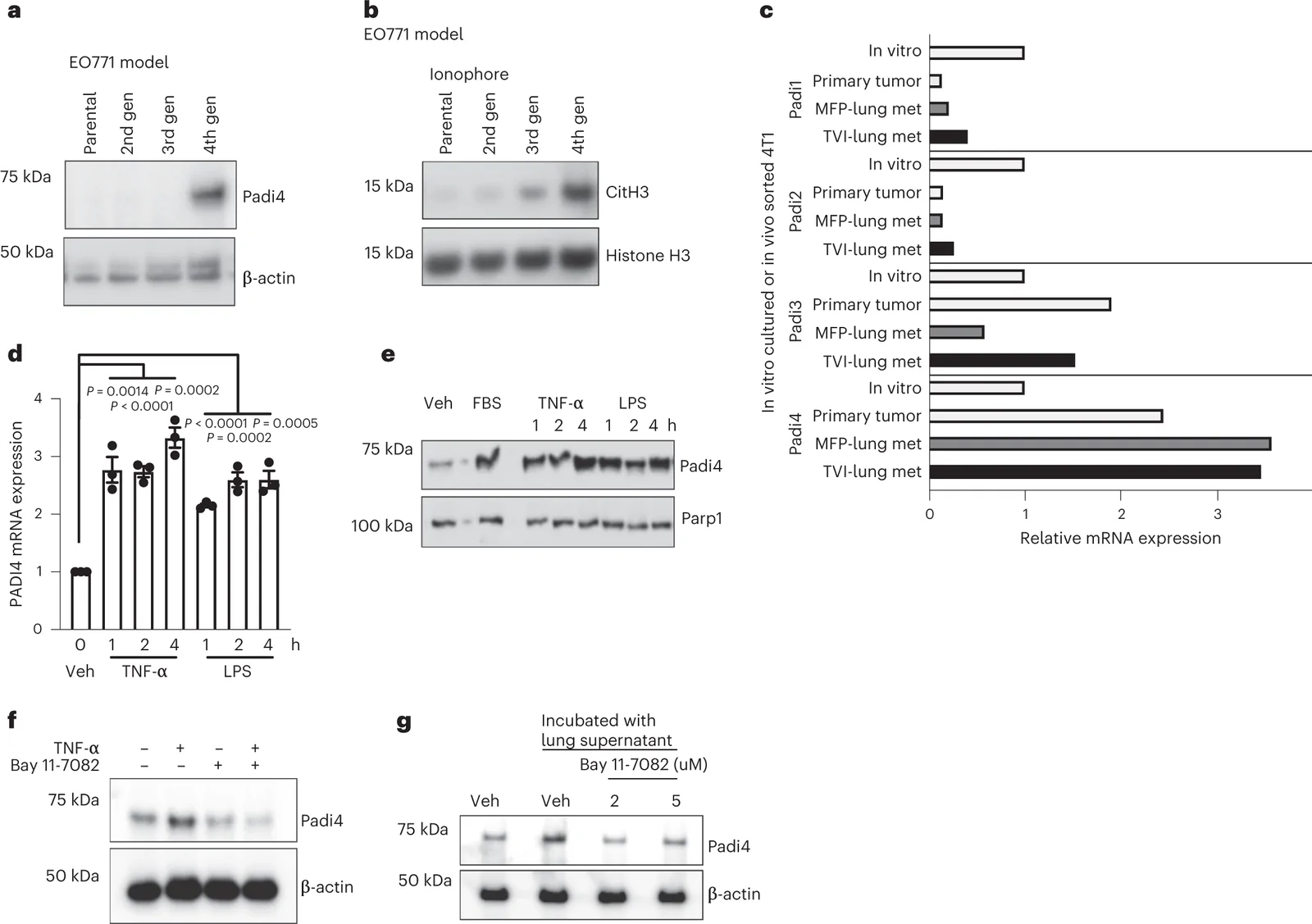
Inflammatory lung microenvironment is critical in Padi4 induction. **a**,**b**, Generation of highly metastatic derivatives of EO771. Padi4 western blot (**a**) or CitH3 (**b**) in EO771 parental and its metastatic derivatives, LM2 (second generation), LM3 (third) and LM4 (fourth). **c**, mRNA expression of Padi genes from in vitro cultured and in vivo sorted 4T1 cells, *n* = 3 biologically independent experiments. **d**, Fold increase of Padi4 mRNA upon treatments of TNF-α or LPS with indicated time. **e**,**f**, Padi4 western blot of 4T1 cells treated with TNF-α or LPS (**e**) and with or without NF-κB inhibitor BAY 11-7082 (**f**). **g**, Padi4 western blot of 4T1 cells incubated with lung supernatant from mice bearing 4T1 tumors (day 30) with or without BAY 11-7082. All data are represented as mean ± s.e.m. and *P* values are based on two-tailed Student’s *t*-test. Western blotting and qPCR were repeated at least twice and representative data are shown. Source data

**Extended Data Fig. 8:**
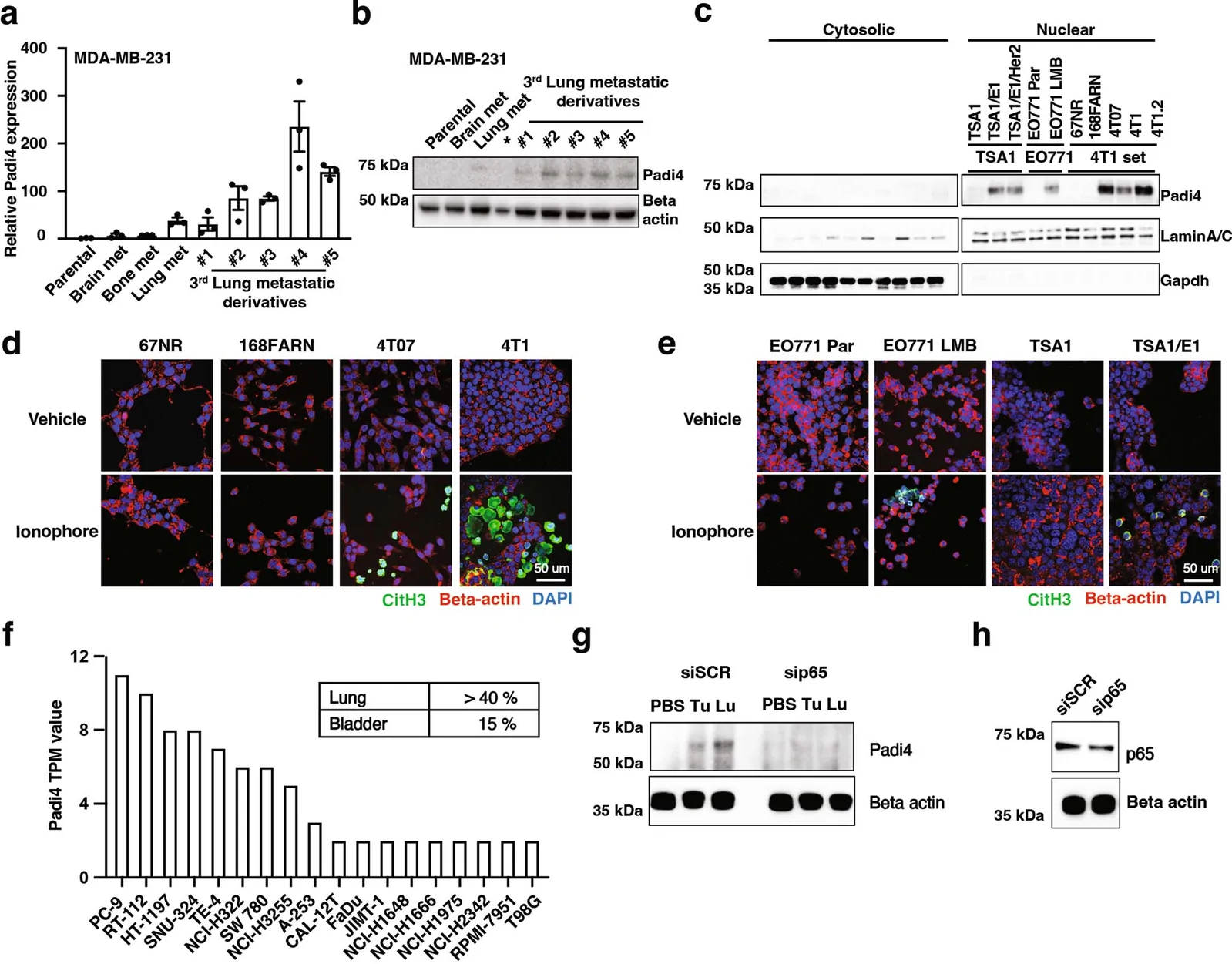
Padi4 and CitH3 are associated with metastatic capacity in breast and lung cancer cell lines. **a** - **b**. Padi4 expression of mRNA (**a**: Q-PCR) or protein (**b**: Western) in metastatic derivatives of human breast cancer MDA-MB-231 cells. Asterisk indicates size ladder lane; *n* = 3 biologically independent experiments. **c**. Padi4 Western of cytosolic or nuclear protein extraction from murine breast cancer cell lines including series of 4T1, EO771 and TSA1. **d** - **e**. IF of CitH3 in various murine breast cancer cell lines including series of 4T1 (**d**), EO771 and TSA1 (**e**) with A23187 ionophore treatment. **f**. Padi4 mRNA expression among human cancer cell lines. **g** - **h**. Padi4 (**g**) and p65 (**h**) Western of 4T1 cells with or without p65 knockdown upon incubation with tumor or lung tissue conditioned media. All data are represented as mean ± s.e.m., Western blotting, qPCR and IF were repeated at least twice, and representative data are shown. Source data

We next tried to understand how Padi4 is regulated, as nuclear expulsion requires high levels of Padi4. CitH3 states and Padi4 expression were profiled in several metastatic variant cell lines. The serially enriched lung metastatic line EO771-LM4 from EO771 parental cells, displayed an increased level of Padi4 (Fig. 6a) and were CitH3 positive when treated with ionophore (Fig. 6b). These results were also observed in MDA-MB-231-LM3 when compared to parental MDA-MB-231 or bone/brain metastatic derivatives (Extended Data Figs. 1c and 8a,b). Metastatic variants of murine cancer cell lines TSA1/E1, 4T1 and 4TO7 but not non-metastatic TSA1, 67NR and 168FARN also showed higher Padi4 (Extended Data Fig. 8c) and CitH3 upon ionophore treatment (Extended Data Fig. 8d,e). Moreover, RNA-seq of lung metastatic tumor cells revealed increased Padi4 messenger RNA but not in other Padi family members (Fig. 6c). Notably, in the human cancer cell line atlas, more lung cancer cell lines showed high Padi4 expression compared to other solid tumors, which is consistent with our CitH3 results (Extended Data Figs. 1c–e and 8f). These results indicate that the lung microenvironment likely potentiates nuclear expulsion by enriching Padi4-high cells or by directly inducing Padi4 expression. Our previous studies show that the lung is a highly inflammatory organ and that tumor cells utilize it to their advantage^28^. We thus treated the 4T1 cells with LPS and TNF-α, which increased Padi4 mRNA and protein levels (Fig. 6d,e). Blocking NF-κB signaling with BAY 11-7082 diminished Padi4 upregulation (Fig. 6f). In addition, conditioned medium from the lungs of 4T1 tumor-bearing mice clearly increased Padi4 and both a NF-κB inhibitor and p65 knockdown reversed this effect (Fig. 6g and Extended Data Fig. 8g,h). Collectively, these results suggest that inflammatory mediators in the lungs enhance Padi4 expression thus potentiating tumor cell nuclear expulsion.

### Human NEP markers and patient prognosis by NEP signature

**Fig. 7:**
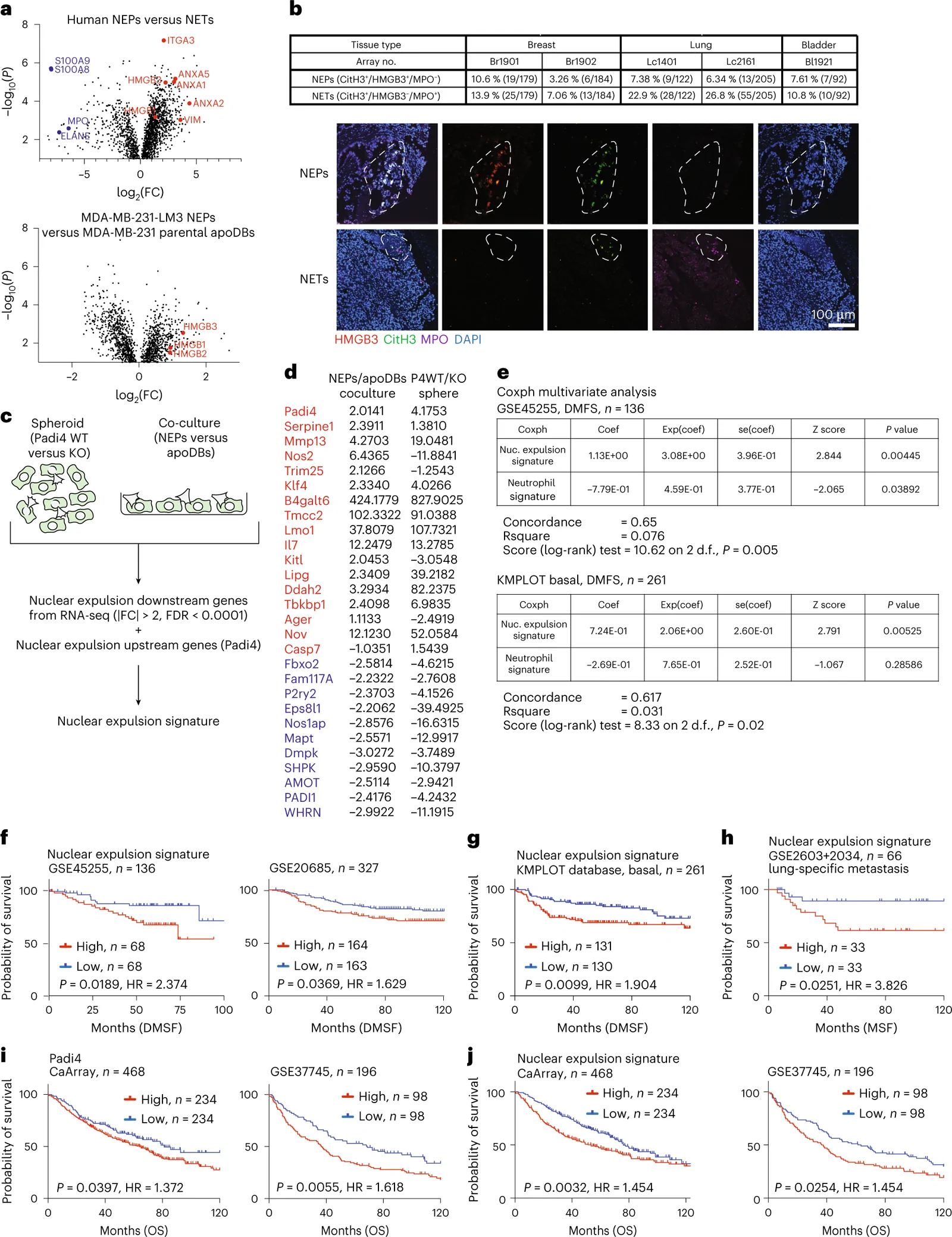
Nuclear expulsion molecular signature correlate with poor prognosis. **a**, Volcano plots of proteomics analysis −log_10_(*P*) versus the log_2_ expression levels comparing MDA-MB-231-LM3 NEPs with NETs (top) or apoDBs (bottom) from MDA-MB-231. Mass spectrometry was performed using biologically independent samples (*n* = 3). **b**, Quantification of NEPs and NETs in patient tumor tissue arrays from multiple types of cancers. CitH3^+^HMGB3^+^ or CitH3^+^MPO^+^ were stained for NEPs and NETs, respectively. Representative images of NEPs (top) and NETs (bottom) are shown. Dashed lines represent NEP or NET boundaries. **c**, Schematic design for generating nuclear expulsion signature. **d**, List of nuclear expulsion molecular signature and fold change in two experimental conditions from RNA-seq. **e**, Multivariate analysis for DMFS in patients with breast cancer from GSE45255 (top) or KMPLOT (bottom) database. Nuclear expulsion signature and neutrophil signature were used as covariates for the Cox proportional hazard model. **f**, DMFS with high or low nuclear expulsion signature in patients with breast cancer from the GSE45255 (left) or GSE20685 (right) datasets. **g**, DMFS with high or low nuclear expulsion signature in basal type of patients with breast cancer from KMPLOT database. **h**, Lung-specific metastasis-free survival (lung-MFS) of patients with TNBC with high or low nuclear expulsion signature within GSE2603+2034 dataset. **i**,**j**, Overall survival (OS) of patients with lung cancer with high or low Padi4 (**i**) or with high or low nuclear expulsion signature (**j**) from CaArray (left) and GSE37745 (right). All *P* values are based on log-rank (Mantel–Cox test).

**Extended Data Fig. 9:**
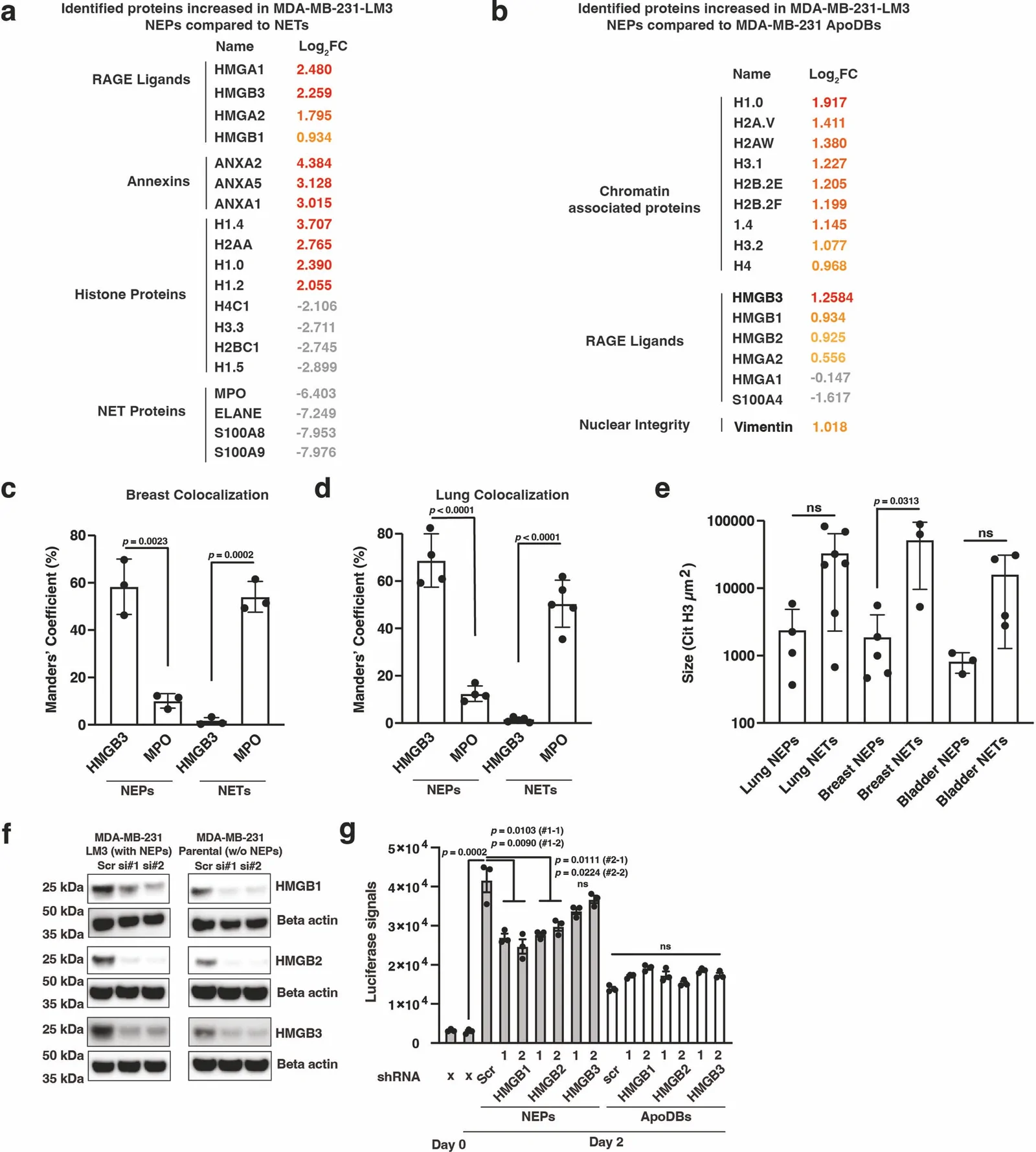
Proteomic analysis of human NEPs and NETs, and validation of NEPs-specific markers. **a**. List of differential proteins from MDA-MB-231-LM3 NEPs compared to NETs. **b**. List of differential proteins from MDA-MB-231-LM3 NEPs compared to MDA-MB-231-Parental derived ApoDBs. **c** - **d**. Manders’ overlap coefficient values of CitH3 and HMGB3 or MPO in breast **(c)** and lung **(d)** cancer tissue arrays; *n* = independent samples. (c): *n* = 3; (d): NEPs, *n* = 4; NETs, *n* =5. **e**. Size of NETs and NETs found in lung, breast, and bladder cancers from human tissue arrays; *n* = independent samples. Lung NEPs, *n* = 4; Lung NETs, *n* = 7; Breast NEPs, *n* =5; Breast NETs, *n* = 3; Bladder NEPs, *n* = 3; Bladder NETs, *n* = 4. **f**. Western blots of HMGB1, 2 and 3 in icas9-MDA-MB-231-Parental and LM3. **g**. Luciferase signals of tumor cell growth of MDA-MB-231 cells co-cultured with HMGB1, 2 or 3 depleted NEPs or apoDBs; *n* = 3 biologically independent experiments. All data are represented as mean ± s.e.m., and p values are based on two-tailed student’s t-test. Western blotting was repeated at least twice, and representative data are shown. Source data

We next examined human tumor cell NEP molecular mediators and tried to distinguish from NETs by tandem mass spectrometry of MDA-MB-231-LM3 NEPs and human derived NETs. MPO, ELANE, S100A8 and S100A9 were abundant in NETs but not in NEPs (Fig. 7a and Extended Data Fig. 9a). Rather, NEPs had highly specific markers such as human RAGE ligand (HMGB3) (Fig. 7a and Extended Data Fig. 9a). In addition, when compared to apoDBs, RAGE agonists HMGB1, HMGB2 and HMGB3 in NEPs were also enriched from proteomics analysis of human NEPs (Extended Data Fig. 9b). Further, IF staining of tumor tissue arrays from 782 patients with breast, lung and bladder cancer revealed two distinct populations: CitH3^+^MPO^+^ and CitH3^+^HMGB3^+^. There was no significant overlap between by Manders’ overlap coefficient, suggesting that HMGB3 is specific to human tumor cell NEPs and that NEPs are distinguishable from NETs (Extended Data Fig. 9c,d). Notably, the CitH3^+^HMGB3^+^ signals seemed smaller in size than CitH3^+^MPO^+^ signals, while still averaging more than 1,000 μm^2^ (Extended Data Fig. 9e). The percent of patients with CitH3^+^HMGB3^+^ and CitH3^+^MPO^+^ was further quantified in the two breast, two lung as well as one bladder tumor tissue arrays. They range from approximately 3–11% for NEPs and 7–27% for NETs (Fig. 7b), suggesting a rather generalized NEP presence in human cancers considering the limited sampling from the core needle biopsy. MDA-MB-231 cells co-cultured with Hmg1, Hmg2 or Hmg3-depleted NEPs showed decreased cell growth, with the most effect from HMGB1 and HMGB2 depleted NEPs (Extended Data Fig. 9f,g). These results suggest that HMGB1 and HMGB2 play a role in RAGE-mediated tumor growth in humans.

**Extended Data Fig. 10:**
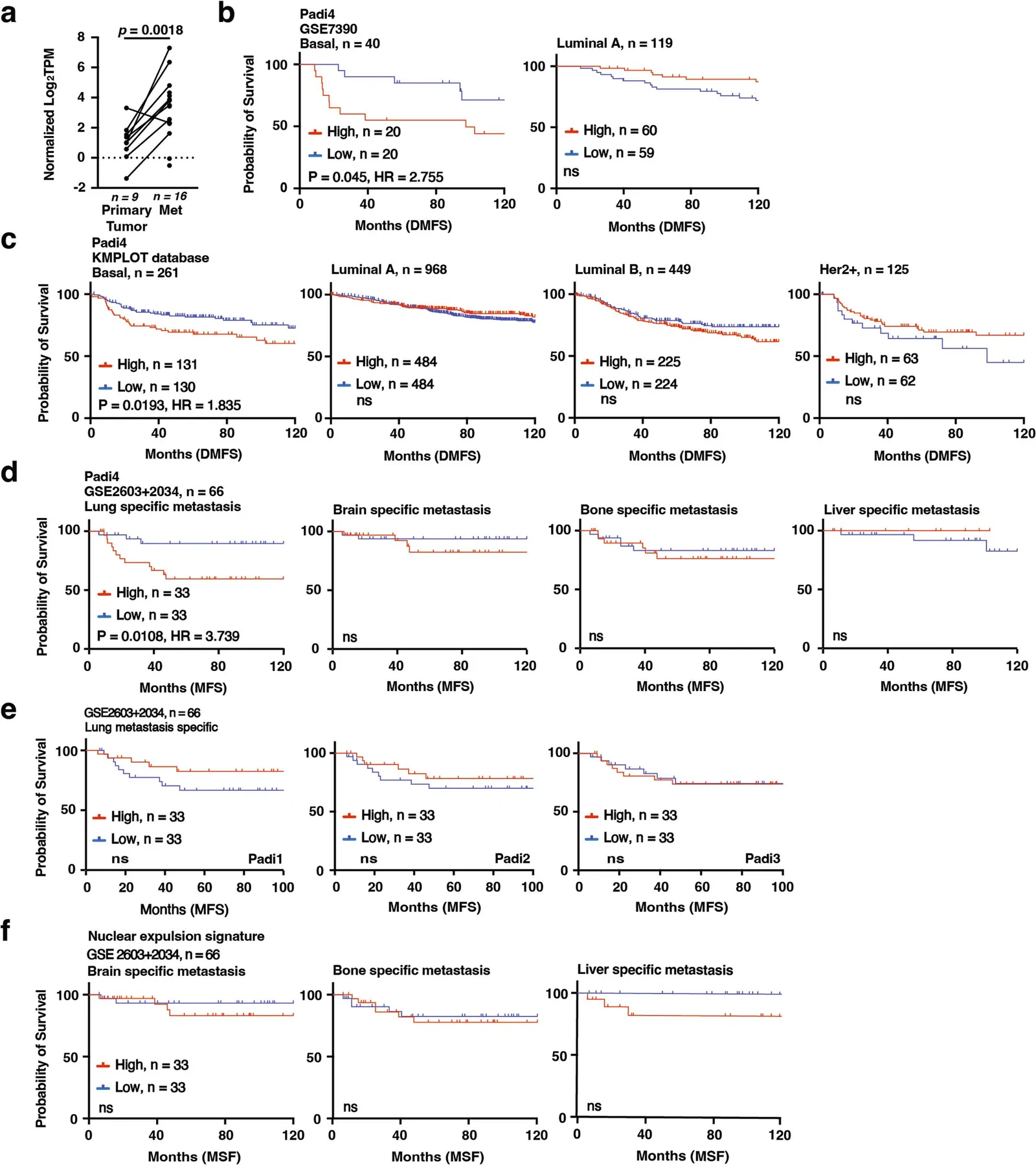
Padi4 expression correlates with poor prognosis of lung metastasis in TNBC patients. **a**. Paid4 expression levels in paired primary tumor and metastases from breast cancer patients. *n* = patients. p value was calculated by two-tailed paired t-test. **b**. Distant metastasis free survival (DMFS) with high or low Padi4 in Basal (**left**), or Luminal A (**right**) types of breast cancer patients within GSE7390 database. **c**. Distant metastasis free survival (DMFS) with high or low Padi4 in Basal, Luminal A, Luminal B, or Her2+ types of breast cancer patients (KMPLOT database). **d**. Metastasis free survival (MFS) of triple negative breast cancer patients with high or low Padi4 in lung, brain, bone, or liver in GSE2603+2034 dataset. **e**. DMFS of triple negative breast cancer patients with lung specific metastasis separated by high and low Padi1, 2 and 3 in GSE2603+2034 dataset. **f**. Metastasis free survival (MFS) of triple negative breast cancer patients with high or low nuclear expulsion signature in Brain, Bone or Liver in GSE2603+2034 dataset. p values for survival curves are based on log-rank (Mantel-Cox) test.

We further investigated the correlation of NEPs with clinical prognosis using various breast and lung cancer patient cohorts. First, an increase in Padi4 expression was observed in matched metastases versus primary tumors (Extended Data Fig. 10a). The basal subtype but not the luminal or Her2^+^ breast cancers showed significant correlation of Padi4 expression with distant metastasis-free survival (DMFS) (Extended Data Fig. 10b,c). Notably, Padi4-high groups showed a decreased DMFS specifically for lung but not for brain, bone or liver metastasis in patients with triple negative breast cancer (TNBC) (Extended Data Fig. 10d). Other Padi family genes did not show similar survival correlation in the same cohort (Extended Data Fig. 10e). Second, to establish a nuclear expulsion signature, we performed RNA-seq on two in vitro systems: the spheroid culture of Padi4^WT^ and Padi4^KO^ cells and Padi4^KO^ cells co-cultured with NEPs or apoDBs (Fig. 7c). The combination of the differentially expressed genes shared by both spheroid culture and 2D co-cell culture with genes upstream of apoptosis-induced nuclear expulsion such as Padi4, was used as our nuclear expulsion signature (Fig. 7d). Using a Cox proportional hazards model for multivariate analysis within patients with breast cancer, we found that the nuclear expulsion signature correlated with metastatic risk independent from the neutrophil signature published (Fig. 7e)^29^. Consistently, the elevated nuclear expulsion signature clearly correlated with decreased survival in two independent breast cancer cohorts (Fig. 7f). In addition, the nuclear expulsion signature also predicted decreased survival in basal or patients with TNBC that was specific for lung but not for brain, bone or liver metastasis (Fig. 7g,h and Extended Data Fig. 10f). Notably, both Padi4 and the nuclear expulsion signature showed worse overall survival specific for patients with lung cancer (Fig. 7i,j). Together, these clinical studies support our hypothesis that apoptosis-induced nuclear expulsion in tumor cells promotes metastatic outgrowth.

## Discussion

Our studies demonstrate that Padi4-expressing apoptotic cancer cells undergo nuclear expulsion and release a DNA/protein structure or NEPs that promotes metastatic outgrowth. Thus, dying cancer cells have a beneficial effect on nearby live cancer cells through an unreported chromatin-bound S100a4. Separation of S100a4 from chromatin diminished the effect of NEPs. In addition, targeting NEPs using DNase, monoclonal antibody S100a4 or sRAGE decreased tumor outgrowth.

Tumor cell-derived NEPs have distinctive components and mechanisms. Chromatin-bound S100a4 mediates NEPs effect on metastatic outgrowth and has not been found to be attached to NETs; and tumor cell NEPs do not have NET-associated enzymes such as NE or MPO, which are essential for NET functions. In addition, apoptosis is critical for nuclear expulsion induction in tumor cells, which is not thought to induce NET formation^30,31^. Furthermore, the effect of NETs on cancer are mostly associated with highly inflamed conditions such as LPS inhalation or smoking to affect lung metastases^15,16,32^, whereas NEPs occur in tumor-bearing conditions without artificial stimulation. Notably, when compared side by side, tumor-specific, but not myeloid-specific, Padi4 deletion substantially decreased lung metastasis (Fig. 3h). For clinical relevance, our nuclear expulsion signature correlates with metastatic risk but not a previously published neutrophil signature (Fig. 7f). We thus caution the cause-and-effect conclusion of NETs on tumor progression.

We first identified that Padi4 is necessary for nuclear expulsion-mediated metastatic outgrowth, which is consistent with the observation that Padi4 promotes lung metastasis through an extracellular chromatin network^33^. Second, we found that either apoptosis or ionophore-mediated calcium influx could activate Padi4. Endoplasmic reticulum calcium channels are critical for calcium regulation during apoptosis^34–36^; however, whether this plays a role in the promotion of apoptosis-induced nuclear expulsion needs further investigation. Third, caspase activation and the accompanied calcium spike are essential for apoptosis-induced nuclear expulsion. The molecular characterization of nuclear expulsion here and above is only a start. It is perceivable that there may be other forms of cellular processes where the nuclear content is expelled into the extracellular space and in many cell types other than tumor cells. Thus, it is tempting to use this new nomenclature of nuclear expulsion and NEPs broadly, covering tumor cells, neutrophils and other cell types, for better clarity in characterization and scientific understanding.

Nuclear expulsion enhanced metastatic outgrowth, but it did not increase the number of metastatic nodules, suggesting it has a higher impact on the micro- to macro-metastasis transition. Chromatin-bound S100a4’s effect on metastatic outgrowth is quite different from intracellular S100a4, which normally plays a role in tumor motility^37,38^. Perhaps this specific effect of S100a4 could be due to its extracellular attachment to chromatin, which may increase the avidity of S100a4 signaling through RAGE on nearby tumor cells. Of note, HMG family proteins such as HMGB1, HMGB2 and HMGB3 are elevated in human tumor cell NEPs in contrast to S100a4 in mice suggesting that NEP signaling through the RAGE pathway may be conserved between mice and humans. In support of this, blocking the RAGE pathway by sRAGE or HMG family knockdown also impacted the effect of human tumor cell NEPs on tumor growth, suggesting that this pathway is shared between mice and humans. RAGE blockade could have a good clinical utilization as *AGER*, a gene encoding the RAGE receptor was found specifically expressed in patients with basal type breast cancer^20^. Our data suggest that Padi4 inhibition or RAGE signaling blockade provides alternative therapeutic options for TNBC that currently lacks effective treatments.

The significance of nuclear expulsion in a clinical therapeutic setting has yet to be realized. Chemotherapy and radiation are known to induce massive amounts of apoptosis and it is well documented that chemotherapy can enhance metastasis and relapse^39–43^. Our observation warrants the need to determine the effect nuclear expulsion can have during treatment and if it contributes to relapse, resistance and therapy-induced metastasis. We anticipate that a combination treatment of chemotherapy and drugs that block the metastasis-promoting effects of nuclear expulsion, such as sRAGE, should be tested in the future.

## Methods

### Mice

BALB/c and C57Bl/6 mice (female, 6–8 weeks old) were purchased from Charles River. Nu/nu and NOD-SCID mice (female, 6–8 weeks old) were purchased from The Jackson Laboratory. The Padi4 flox/flox mouse line was obtained from the Center for Advanced Preclinical Research at the National Cancer Institute (NCI). Padi4 flox/flox mice were bred with LysM-Cre (B6.129P2-Lyz2tm1(cre)Ifo/J) mice from The Jackson Laboratory to generate the Padi4 deletion in myeloid cells (Padi4^MyeKO^). All animal protocols were approved by NCI’s Animal Care and Use Committee, protocol no. LCBG007. A 12-h light–dark cycle was used and temperatures of 65–75 °F (~18–23 °C) with 40–60% humidity were maintained.

### Cell lines

Murine 4T1, 67NR, 167FARN, 4T07 and EO771, as well as human MDA-MB-231 cell lines were purchased from the American Type Culture Collection. A highly metastatic EO771 cell line, EO771-LM4, was established from metastatic lung nodules using four sequential rounds of EO771 tumor transplantation. EO771-LMB, which is another highly metastatic cell line of EO771, was gifted by R.L. Anderson. A highly lung metastatic MDA231-lung met-3 (LM3) was also established from metastatic lung nodules by additional enrichment from MDA-MB-231 LM2 and BrM2 cell line, which was gifted by J. Massague. PC9, a human lung cancer cell line, was gifted by J. Amann and D. Carbone. RT112 and SW780 cells were gifted by P.K. Agarwal. Cells were cultured in DMEM supplemented with 10% heat-inactivated FBS, 500 U ml^−1^ penicillin and 500 mg ml^−1^ streptomycin at 37 °C in a humidified atmosphere containing 5% CO_2_ and confirmed to be *Mycoplasma* negative.

### Padi4 knockout and knockdown

4T1 or EO771 cells were transduced with Pspcas9-2A-puro-px459 (Addgene 48139) constructs containing each of Padi4 single-guide RNAs designed by F. Zhang’s laboratory, which was followed by 2 μg ml^−1^ puromycin selection for 2 d. Immunoblotting and genotyping were used for verifying gene knockout. Primers are listed in Supplementary Table 3.

For Padi4 knockdown, the 4T1 cells were transduced with lentiviral particles in 8 μg ml^−1^ of polybrene for 5 h. After 1 d, cells were selected by 2 μg ml^−1^ of puromycin for 3 d. For Padi4 knockdown, Mission shRNAs (Sigma, TRCN0000101831 and TRCN000101833) were used.

### iCasp9-transduced cell line generation

The 4T1 Padi4^WT^ or Padi4^KO^ tumor cells were transduced with an iCasp9 vector that was made using a Gibson (NEB E5510S) kit and the iCasp9 gene from the EF1α-iCasp9-hΔCD19 vector^44^ was cloned into a pLV-EF1a-IRES-Blast backbone (Addgene 85133).

### Spontaneous or experimental metastasis and intrathoracic lung metastasis model

For orthotopic metastasis, the 2.0 × 10^5^ 4T1 or E0771-LMB/LM4 mammary tumor cells were injected into the no. 2 MFP. The number of lung metastases were evaluated after 4–5 weeks by Indian ink staining. Tumor volume was calculated as tumor volume (mm^3^) = (length in mm) × (width in mm)^2^ × 0.5. The maximal tumor volume permitted by the protocol was 3,000 mm^3^ and the maximum permitted tumor burden was not exceeded.

For experimental metastasis, the 1 × 10^5^ 4T1 cells were injected through the tail vein. The number of metastatic nodules in lung was evaluated at indicated time points by Indian ink staining.

For intrathoracic lung metastasis, the mixture of 2 × 10^4^ luciferase-expressing 4T1 cells and equivalent NEPs or apoDBs were intrathoracically injected into the left lung. The amounts of NEPs or apoDBs are shown in the ‘Preparation of NEPs or apoptotic debris from tumor cells’ section; these were injected directly into the left side of the lungs. Luminescent signals of metastases burdens were imaged with an IVIS system after retro-orbital injection of luciferin. In vivo Padi4 inhibition was achieved using intraperitoneal administration of GSK-484 (4 mg kg^−1^) into a 4T1 MFP mouse model. DNase I (10,000 U kg^−1^) or sivelestat (an elastase inhibitor, 50 mg kg^−1^) was used for blocking DNA-based structures for both NEPs and NETs or only NETs, respectively.

For in vivo NEP depletion, anti-mouse S100A4 (6B12, 100 μg per mouse) or mouse IgG1 isotype control (MOPC-21, 100 μg per mouse) as well as soluble RAGE peptide (10 μg per mouse) were intraperitoneally injected every other day after intrathoracic injection of tumor cells. All mice were randomized before treating with drugs or NEPs. No statistical method was used to predetermine sample size and no data exclusions were used.

### Flow cytometry

Lungs were collected from mice that received co-injection of tumor cells with NEPs or apoDBs. Lungs were minced and incubated with dissociation buffer (plain DMEM containing 1 mg ml^−1^ of collagenase, 120 μg ml^−1^ of dispase and 0.15 mg ml^−1^) for 45 min with rotation (150 r.p.m. at 37 °C). Dissociated tissues were then filtered by 70-μm cell strainer and washed with MACS buffer (PBS containing 2% FBS and 1 mM of EDTA). Red blood cells were removed by incubation with ACK buffer. After washing with MACS buffer, cells were resuspended with MACS buffer containing 7AAD, followed by staining with primary antibodies. Antibody information is included in the Reporting summary. Data were analyzed with FlowJo and gating strategies are shown in Supplementary Fig. 1.

#### Cell cycle analysis

Padi4^WT^ or Padi4^KO^ cells (3 × 10^5^) were seeded into six-well plates and allowed to grow for 24 h. Cells were then, incubated with 5 μM 5-ethynyl-2'-deoxyuridine (EdU) for 1 h After incubation, cells were detached and fixed with 70% ethanol for 2 h at −20 °C. Fixed cells were washed and subjected into Click-iT EdU Alexa Fluor 647 Flow Cytometry Assay kit followed by incubation with propidium iodide and RNase. Flow cytometry was used to measure distribution of the cell cycle.

### Imagestream

H2B-GFP-tagged Padi4WT or Padi4KO 4T1 cells were injected through the tail vein and the lungs were collected 24 h after injection. Dissociated cells were then stained with Magic Red active caspase-3/7 (Abcam, ab270771) and Alexa Fluor-647-conjugated CitH3 (Abcam, ab237374). The fluorescence intensities were measured with Amnis ImageStream MkII (Luminex Corporation). The images were generated and analyzed by INSPIRE software (Luminex Corporation).

### Immunoblotting

For detecting citrullination of cells upon treatment with A23187 ionophore, Raptinal or AP1903, cells were seeded, starved and treated with 2 μM, 10 μM or 5 nM of the above drugs for 2, 6 and 4 h, respectively. For checking cell death signals, including Gsdm D/E, Mlkl, caspase-3 or Parp1, cells were starved overnight and treated with A23187 ionophore, Raptinal or AP1903 and Shikonin (10 μM) for 6 h and 12 h. For necroptosis induction, the starved and TNF-α-primed (4 h) 4T1 tumor cells were treated with pan-caspase inhibitor QVD-OPh (10 μM) before Navticlax (10 μM) addition. For pyroptosis induction, LPS (1 μg ml^−1^)-primed 4T1 or Raw264.7 cells were treated with Nigericin (10 μM) or 4T1 or Raw264.7 cells were treated with Val-boroPro (10 μM). AC-YVAD-cmk (10 μM) was used to block caspase-1-dependent pyroptosis. For caspase-11-dependent pyroptosis induction in the 4T1 or Raw264.7 cells, LPS transfection by Lipofectamin was performed. Cells were collected and incubated with Triton Extraction Buffer (TEB; PBS containing 0.5% Triton X-100 (v/v), 0.02% (w/v) NaN3) at a cell density of 10^7^ cells per ml for 10 min. Cells were then centrifuged and resuspended in 0.2 N HCl at a density of 4 × 10^7^ nuclei per ml for 16 h. Supernatants were neutralized by 2 M NaOH at 1:10 of the volume of the supernatant and used for further western blot. A NE-PER Nuclear and Cytoplasmic Extraction kit (Thermo, cat. no. 78835) was used for collecting nuclear or cytosolic extracts per manufacture instruction. Whole cell lysates were collected using RIPA lysis buffer (20 mM, pH 7.4, Tris-HCl, 150 mM NaCl, 1 mM EDTA, 0.01% NP-40, 0.01% Triton X-100 and 0.5% of deoxycholate). Antibodies used in this study are listed in the Reporting summary. Image Lab v.6.0.1. was used for analyzing images.

### Immunofluorescence

The tumors and lungs were surgically resected, incubated in 4% paraformaldehyde at 4 °C overnight, embedded in OCT and sliced into 10-μm thick increments. For staining tissue arrays, slides were deparaffinized and antigen retrieval was performed with citrate buffer (H-3300, Vectorlab). Hmgb3 or Mpo with CitH3 were used for staining NEPs or NETs, respectively. Quantitation analysis of colocalization between Hmgb3 or Mpo and CItH3 was performed using the Manders’ overlap coefficient in the JACoP plugin for ImageJ^45^. Breast (Br1901 and Br1902), lung (Lc1401 and Lc2162) and bladder (Bl1921) samples were purchased from Biomax. For staining in vitro co-culture tumor cells with NEPs, cells were seeded in a chamber slide for 24 h. NEPs prepared from the iCasp9 system were added to the culture medium for 24 h. Co-cultured cells were fixed by 4% paraformaldehyde, immunofluorescence stained and followed by microscopic analysis with a Nikon SORA microscopy system. Quantification was performed blind. Antibodies used in this study are listed in the Reporting summary.

### Time-lapse imaging

Cells were cultured in DMEM + 10% FBS for 48 h and serum-starved 16 h before imaging with a final confluency around 70%. Medium was changed to Fluorobite DMEM before imaging. Epifluorescence images were taken on a Nikon Ti-ZE microscope with a Hamamatsu Flash 4 V3 at ×20 at 37 °C in 5% CO_2_. Confocal images were taken on a Nikon SoRa Spinning Disk with a Photometrics BSI sCMOS camera at ×20 at 37 °C in 5% CO_2_. Custom MATLAB scripts were used for tracking the cells and for analysis.

### Expulsion algorithm

For each cell trace we found the numerical gradient of the nuclear expansion over different intervals of time to determine the maximum increase in size or expulsion. To find a threshold above which a cell is deemed to have gone through nuclear expulsion, a receiver operating characteristic curve was implemented with Padi4^WT^ cells as the positive condition and Padi4^KO^ cells as the negative. All original code has been deposited at Figshare at https://doi.org/10.6084/m9.figshare.14832234.

### Calcium tracking

Cells were incubated with X-Rhod-1AM (Thermo, X14210) at a 1:4,000 dilution for 10 min, washed twice with PBS and the cells were imaged in widefield at ×10 with the cells in serum-free medium. Using our custom MATLAB tracking software we masked every cell using the H2B:GFP signal and measured the calcium inside this area.

### Preparation of NEPs or apoptotic debris from tumor cells

Cells were cultured in DMEM + 10% FBS and allowed to grow to 90% confluence followed by serum starvation for 16 h. Cells were detached, washed with PBS three times and were then resuspended with 1 ml 1 mM CaCl_2_ containing DPBS for mouse cells or RPMI for human cells and plated at a concentration of 1 × 10^7^ per ml on a 12-well plate. Raptinal or A23187 ionophore was added for 4 h for mouse cells and 11 h for human cells to induce nuclear expulsion. For NEPs or apoDBs with Hmg family knockdown, 20 nM of each siRNA for Hmgb1, Hmgb2 and Hmgb3 was transfected using Lipofectamine 3000 into iCas9-carrying MDA-MB-231 cells. With a treatment of 8 nM AP1903, iCasp9-transduced Padi4^WT^ or Padi4^KO^ cells were used for enriching NEPs or apoDBs, respectively. NEPs or apoDBs were added to recipient cells in the following amounts, co-culture (NEPs/apoDBs from 5,000 cells to 500 recipient cells), sphere formation (NEPs/apoDBs from 30,000 cells to 2,000 recipient cells) and intrathoracic injection (NEPs/apoDBs from 200,000 cells to 20,000 recipient cells). BCA assay was used to verify NEPs and apoDB concentration. For proteomics, following incubation with drugs, NEPs were additionally treated with 10 units of MNase for 10 min to solubilize attached proteins. Samples were centrifuged at 5,000*g* for 5 min. The cleared supernatant was then stored at −80 °C.

### Preparation of NETs for proteomics

Neutrophils were isolated as previously described^46^. In a 12-well plate in 1 ml RPMI (Thermo, 11835030) 1 × 10^7^ neutrophils from three separate patients were stimulated with ionophore for 4 h. Carefully, 900 μl of the supernatant was removed and 300 μl of RPMI with 10 units of MNase was used to treat NET samples for 10 min. The sample was centrifuged at 5,000*g* for 5 min. The cleared supernatant was then stored at −80 °C.

### NEPs LC–MS proteomics

For TCA precipitation and trypsin digestion, 100% TCA was added to 10 μg of protein in 100 μl of H_2_O and incubated on ice for 1.5 h. The samples were centrifuged and supernatants removed. Thermo EasyPrep digestion kit (PNA40006) was used to solubilize the pellet in which the pellets were resuspended in 100 μl of the lysis buffer, with addition of 10 μl 1 M HEPES, pH 8.0. The samples were sonicated in a 37 °C water bath for 10 min, resulting in no visible pellet. BCA was used to determine protein concentration. Then, 50 μl of reduction solution and 50 μl of alkylation solution from the kit were added and the samples were incubated for 10 min at 95 °C and then allowed to cool to room temperature. Then, 500 μl of enzyme reconstitution solution was added to each vial of 100 μg trypsin/Lys-C, then added to 50 μl of each sample and incubated at 37 °C for 3 h.

For TMT labeling, trypsinized samples were labeled with the designated TMTpro label. The label stocks were 3 μg μl^−1^ in 100% ACN and 40 ul of the designated label was added to the samples for a ratio of 10:1 (label:peptide) and incubated for 1 h at room temperature. The TMTpro reagents were quenched with 50 μl of 5% hydroxyamine, 20% formic acid and incubated for 10 min at room temperature. Then, 1 μg of peptide from each sample was taken and mixed together in a clean tube. The cleanup columns from the EasyPrep kit were used for all 16 samples, which were then dried in the speedvac and stored at −80 °C.

For LC–MS analysis of TMT-labeled samples, the TMT-labeled pellet was resuspended in 40 μl of 0.1% GA and 10 μl was injected 3× on the QE-HF using a 2-h gradient. Raw files were searched in PD2.4 using the Reporter Quan nodes. Pathway analysis of the samples was performed using Gene Set Enrichment Analysis v.4.0.3 using the pre-ranked functionality was calculated using the formula 1 / *P* value for the different comparisons.

### NEP purification using dialysis

Dialysis tubes (300 kD, Spectra, 131450) were prepared according to the manufacturer’s instructions and equilibrated in 20 mM HEPES. NEP samples were added to the tubes and placed in 4 °C in 100 ml 20 mM HEPES. NaCl concentration was gradually increased up to 200 mM using a peristaltic pump over 16 h. Samples were equilibrated back to physiological salt conditions in 1-kD dialysis tubes (Spectra, 131090) over 4 h.

### RNA-seq and generation of the nuclear expulsion gene signature

RNA was extracted from Padi4^KO^ cells co-cultured with NEPs or apoDBs, as well as Padi4^WT^ or Padi4^KO^ spheroids using Zymo Quick-DNA/RNA Miniprep. Samples were sequenced on an Illumina NextSeq. Quantification, quality control, differential expression and pathway analysis were performed using the CCBR Pipeliner (https://github.com/CCBR/Pipeliner).

To generate the nuclear expulsion signature, we identified 200 genes that were differentially expressed in both the co-culture and spheroid groups by selecting genes that were upregulated using log_2_(FC) > 2 or downregulated using log_2_(FC) < −2 and both with FDR < 0.0001. Among these 200 genes, 28 genes were selected as nuclear expulsion signature by gene function, upstream or downstream of nuclear expulsion and single gene clinical relevance. Finally, the 28-gene signature was examined for clinical correlation using significance for DMFS in KMPLOT, GSE2603 +2034 of breast cancer as well as OS in CaArray and GSE37745 of lung cancer cohorts. A Cox proportional hazards model was used to validate significance and specificity of nuclear expulsion signature.

### Spheroid culture

Tumor cells (2,000) were added to a U-bottomed ULA plate (Thermo, 174925) in 5% FBS in Fluorobrite DMEM. NEPs were added and incubated with the cells for 20 min. The plate was then centrifuged at 400*g* for 10 min and placed on the Incucyte S3 for imaging every 6 h. After 3 d, the medium was removed and 40 μl 75% Matrigel was added. sRAGE and S100a4 were added directly to the Matrigel and the medium in their respective conditions. The plate was then incubated for 30 min in 37 °C to polymerize the Matrigel. Then, 100 μl 2% FBS Fluorobrite DMEM was added to the well. Medium was changed every 3 d.

### Cell proliferation, viability and adhesion when co-cultured with NEPs

Tumor cells (5 × 10^2^) were seeded in triplicate into 96-well plates and cell viability was measured after 2 d using a luminescent-based cell viability assay kit (Cell-Titer-Glo, Promega, G7572) or MTT assay kit (Promega, No. G4000). For co-culture of tumor cells with NEPs or apoDBs, 5 × 10^2^ of Padi4^KO^ 4T1 cells or MDA-MB-231 parental cells were plated and NEPs or apoDBs, generated using the iCasp9 system, were added to cells 1 d after seeding. For dissecting the downstream signaling pathways, RAGE antagonist peptides (sRAGE, 10 μM), S100A4-neutralizing antibody (5 μg ml^−1^) or MAPK inhibitors (Selleckchem, trametinib (S2673) and selumetinib (1008)) were added to the cell culture 2 h before NEPs were added.

For the adhesion assay, NEPs or apoDBs were pre-coated onto 96-well plates 4 h before tumor cells were seeded. Then, 2 × 10^3^ tumor cells were then plated in triplicate and cells were washed with PBS three times at 3 h and 6 h. The number of remaining cells was measured by Cell-Titer-Glo assay kit.

### RT–qPCR

Total RNA from 5 × 10^5^ tumor cells were collected 24 h after seeding and purified using Qiazol according to the manufacturer’s protocol. In some experiments, 2 × 10^5^ tumor cells were seeded onto a six-well plate and cultured for 16 h, starved for an additional 24 h and treated with 20 ng ml^−1^ TNF-α and 5 μM BAY 11-7082 (NF-κB inhibitor) for 4 h and 8 h.

Then, 400 ng of total RNA was reverse transcribed using a high-capacity complementary DNA reverse transcription kit (Applied Biosystems) and roughly 10 ng of the resulting cDNA was then mixed with SYBR green PCR Master Mix (Applied Biosystems) and the appropriate primers. mRNA expression was quantified by performing RT–qPCR using a Quantstudio 6 Flex Real-Time PCR System (Applied Biosystems). Mouse or human GAPDH was used as an endogenous control for normalization of SYBR. Primers used are listed in Supplementary Table 3. Data were analyzed with CFX Manager.

### Statistics and reproducibility

GraphPad Prism v.9 was used for statistics. Unless otherwise indicated, all data were analyzed based on a two-tailed Student’s *t*-test and expressed as mean ± s.e.m. Statistical analysis of survival data used the log-rank (Mantel–Cox) test. Differences were considered statistically significant for *P* < 0.05. Event numbers and statistical details are shown in the relevant figure legends. Significance is noted in figures or figure legends; *P* values are denoted as ^∗^*P* < 0.05, ^∗∗^*P* < 0.01, ^∗∗∗^*P* < 0.001, ^∗∗∗∗^*P* < 0.0001, NS > 0.05. Data distribution was assumed to be normal, but this was not formally tested. No statistical methods were used to predetermine sample sizes, but our sample sizes are similar to those reported in previous publications. Data collection and analysis were performed blind to the conditions of quantification in IF experiments but were not performed blind to the conditions of in vivo experiments because the same observer treated and measured tumors and tumors that were treated responded noticeably.

### Materials availability

All reagents generated in this study (including cell lines and plasmids) are available on request to the corresponding author.

### Reporting summary

Further information on research design is available in the Nature Portfolio Reporting Summary linked to this article.

## Supplementary information

Supplementary information Supplementary Figs. 1–4 of flow cytometry gating strategy and Supplementary Tables 1–3 of reagent and primer lists.

Reporting Summary

Supplementary Video 1 4T1 cells undergo nuclear expulsion under A23187 ionophore treatment. Time-lapse imaging of 4T1 upon an A23187 ionophore treatment under starved conditions. Green represents H2B-GFP.

Supplementary Video 2 4T1 cells undergo nuclear expulsion under Raptinal treatment. Time-lapse imaging of 4T1 upon a Raptinal treatment under starved conditions. Green represents H2B-GFP.

Supplementary Video 3 MDA-MB-231-LM3 cells undergo nuclear expulsion under Raptinal treatment. Time-lapse imaging of 4T1 upon a Raptinal treatment under starved conditions. Green represents H2B-GFP.

Supplementary Video 4 Padi4 knockout diminishes nuclear expulsion under A23187 ionophore treatment. Time-lapse imaging of 4T1 Padi4^KO^ cells upon an A23187 ionophore treatment under starved conditions. Green represents H2B-GFP.

Supplementary Video 5 Padi4 knockout diminishes nuclear expulsion under Raptinal treatment. Time-lapse imaging of 4T1 Padi4^KO^ cells upon an A23187 ionophore treatment under starved conditions. Green represents H2B-GFP.

Supplementary Video 6a Caspase-3 knockdown phenocopies loss of Padi4 in EO771-LMB cells. Time-lapse imaging of Padi4^WT/KO^ of EO771-LMB with or without caspase-3 knockdown under navitoclax treatment under starved conditions. Padi4^WT^ and Caspase-3^WT^ (a), Padi4^WT^ and Caspase-3^KD^ (b), Padi4^KO^ and Caspase-3^WT^ (c) Padi4^KO^ and Caspase-3^KD^ (d). Green represents H2B-GFP.

Supplementary Video 6b Caspase-3 knockdown phenocopies loss of Padi4 in EO771-LMB cells. Time-lapse imaging of Padi4^WT/KO^ of EO771-LMB with or without caspase-3 knockdown under navitoclax treatment under starved conditions. Padi4^WT^ and Caspase-3^WT^ (a), Padi4^WT^ and Caspase-3^KD^ (b), Padi4^KO^ and Caspase-3^WT^ (c) Padi4^KO^ and Caspase-3^KD^ (d). Green represents H2B-GFP.

Supplementary Video 6c Caspase-3 knockdown phenocopies loss of Padi4 in EO771-LMB cells. Time-lapse imaging of Padi4^WT/KO^ of EO771-LMB with or without caspase-3 knockdown under navitoclax treatment under starved conditions. Padi4^WT^ and caspase-3^WT^ (a), Padi4^WT^ and caspase-3^KD^ (b), Padi4^KO^ and caspase-3^WT^ (c) Padi4^KO^ and caspase-3^KD^ (d). Green represents H2B-GFP.

Supplementary Video 6d Caspase-3 knockdown phenocopies loss of Padi4 in EO771-LMB cells. Time-lapse imaging of Padi4^WT/KO^ of EO771-LMB with or without caspase-3 knockdown under navitoclax treatment under starved conditions. Padi4^WT^ and caspase-3^WT^ (a), Padi4^WT^ and caspase-3^KD^ (b), Padi4^KO^ and caspase-3^WT^ (c) Padi4^KO^ and caspase-3^KD^ (d). Green represents H2B-GFP.

Supplementary Video 7a Caspase-3 knockdown phenocopies loss of Padi4 in 4T1 cells. Time-lapse imaging of Padi4^WT/KO^ of 4T1 with or without caspase-3 knockdown under navitoclax treatment under starved conditions. Padi4^WT^ and caspase-3^WT^ (a), Padi4^WT^ and caspase-3^KD^ (b), Padi4^KO^ and caspase-3^WT^ (c) Padi4^KO^ and caspase-3^KD^ (d). Green represents H2B-GFP.

Supplementary Video 7b Caspase-3 knockdown phenocopies loss of Padi4 in 4T1 cells. Time-lapse imaging of Padi4^WT/KO^ of 4T1 with or without caspase-3 knockdown under navitoclax treatment under starved conditions. Padi4^WT^ and caspase-3^WT^ (a), Padi4^WT^ and caspase-3^KD^ (b), Padi4^KO^ and caspase-3^WT^ (c) Padi4^KO^ and caspase-3^KD^ (d). Green represents H2B-GFP.

Supplementary Video 7c Caspase-3 knockdown phenocopies loss of Padi4 in 4T1 cells. Time-lapse imaging of Padi4^WT/KO^ of 4T1 with or without caspase-3 knockdown under navitoclax treatment under starved conditions. Padi4^WT^ and caspase-3^WT^ (a), Padi4^WT^ and caspase-3^KD^ (b), Padi4^KO^ and caspase-3^WT^ (c) Padi4^KO^ and caspase-3^KD^ (d). Green represents H2B-GFP.

Supplementary Video 7d Caspase-3 knockdown phenocopies loss of Padi4 in 4T1 cells. Time-lapse imaging of Padi4^WT/KO^ of 4T1 with or without caspase-3 knockdown under navitoclax treatment under starved conditions. Padi4^WT^ and caspase-3^WT^ (a), Padi4^WT^ and caspase-3^KD^ (b), Padi4^KO^ and caspase-3^WT^ (c) Padi4^KO^ and caspase-3^KD^ (d). Green represents H2B-GFP.

Supplementary Video 8a BH3-mimetic-mediated canonical apoptosis causes nuclear expulsion in 4T1 cells. Time-lapse imaging of 4T1 under navitoclax (a), Venetoclax (b) or S63845 (c) treatment under starved conditions. Green represents H2B-GFP.

Supplementary Video 8b BH3-mimetic mediated canonical apoptosis causes nuclear expulsion in 4T1 cells. Time-lapse imaging of 4T1 under navitoclax (a), Venetoclax (b) or S63845 (c) treatment under starved conditions. Green represents H2B-GFP.

Supplementary Video 8c BH3-mimetic mediated canonical apoptosis causes nuclear expulsion in 4T1 cells*.* Time-lapse imaging of 4T1 under navitoclax (a), Venetoclax (b) or S63845 (c) treatment under starved conditions. Green represents H2B-GFP.

## Source data

Source Data Fig. 1 Statistical source data.

Source Data Fig. 1 Unprocessed western blots and/or gels.

Source Data Fig. 2 Statistical source data.

Source Data Fig. 2 Unprocessed western blots and/or gels.

Source Data Fig. 3 Statistical source data.

Source Data Fig. 4 Statistical source data.

Source Data Fig. 5 Statistical source data.

Source Data Fig. 5 Unprocessed western blots and/or gels.

Source Data Fig. 6 Statistical source data.

Source Data Fig. 6 Unprocessed western blots and/or gels.

Source Data Extended Data Fig. 1 Unprocessed western blots and/or gels.

Source Data Extended Data Fig. 3 Statistical source data.

Source Data Extended Data Fig. 3 Unprocessed western blots and/or gels.

Source Data Extended Data Fig. 4 Statistical source data.

Source Data Extended Data Fig. 5 Statistical source data.

Source Data Extended Data Fig. 5 Unprocessed western blots and/or gels.

Source Data Extended Data Fig. 6 Statistical source data.

Source Data Extended Data Fig. 7 Statistical source data.

Source Data Extended Data Fig. 7 Unprocessed western blots and/or gels.

Source Data Extended Data Fig. 8 Statistical source data.

Source Data Extended Data Fig. 8 Unprocessed western blots and/or gels.

Source Data Extended Data Fig. 9 Statistical source data.

Source Data Extended Data Fig. 9 Unprocessed western blots and/or gels.

## Data Availability

RNA-seq data that support the findings of this study have been deposited in the Gene Expression Omnibus under accession code GSE178512. Mass spectrometry data have been deposited in MassiVE at ftp://massive.ucsd.edu/MSV000087691/. The public dataset that supports the findings of this study is available in the Gene Expression Omnibus, KMPLOT or Oncomine under accession codes GSE20685, GSE45255, GSE7390, GSE2603, GSE2634 and GSE37745. Source data for Figs. 1–6 and Extended Data Figs. 3–9 are provided. All other data supporting the findings of this study are available from the corresponding author on reasonable request. Source data are provided with this paper.

## Code availability

All original code that supports the findings of this study have been deposited in Figshare at https://doi.org/10.6084/m9.figshare.14832234.
