# Supplementary material for: Apoptosis-induced nuclear expulsion in tumor cells drives S100a4-mediated metastatic outgrowth through the RAGE pathway
Source: Nat Cancer. 2023 Mar 27;4(3):419–35. doi: 10.1038/s43018-023-00524-z (PMC10042736; doi:10.1038/s43018-023-00524-z)
Supplement: Supplementary file 1 — Supplementary Figs. 1–4 of flow cytometry gating strategy and Supplementary Tables 1–3 of reagent and primer lists. [file 43018_2023_524_MOESM1_ESM.pdf]

nature cancer

Article

https://doi.org/10.1038/s43018-023-00524-z

Apoptosis-induced nuclear expulsion in tumor cells drives S100a4-mediated metastatic outgrowth through the RAGE pathway

In the format provided by the authors and unedited

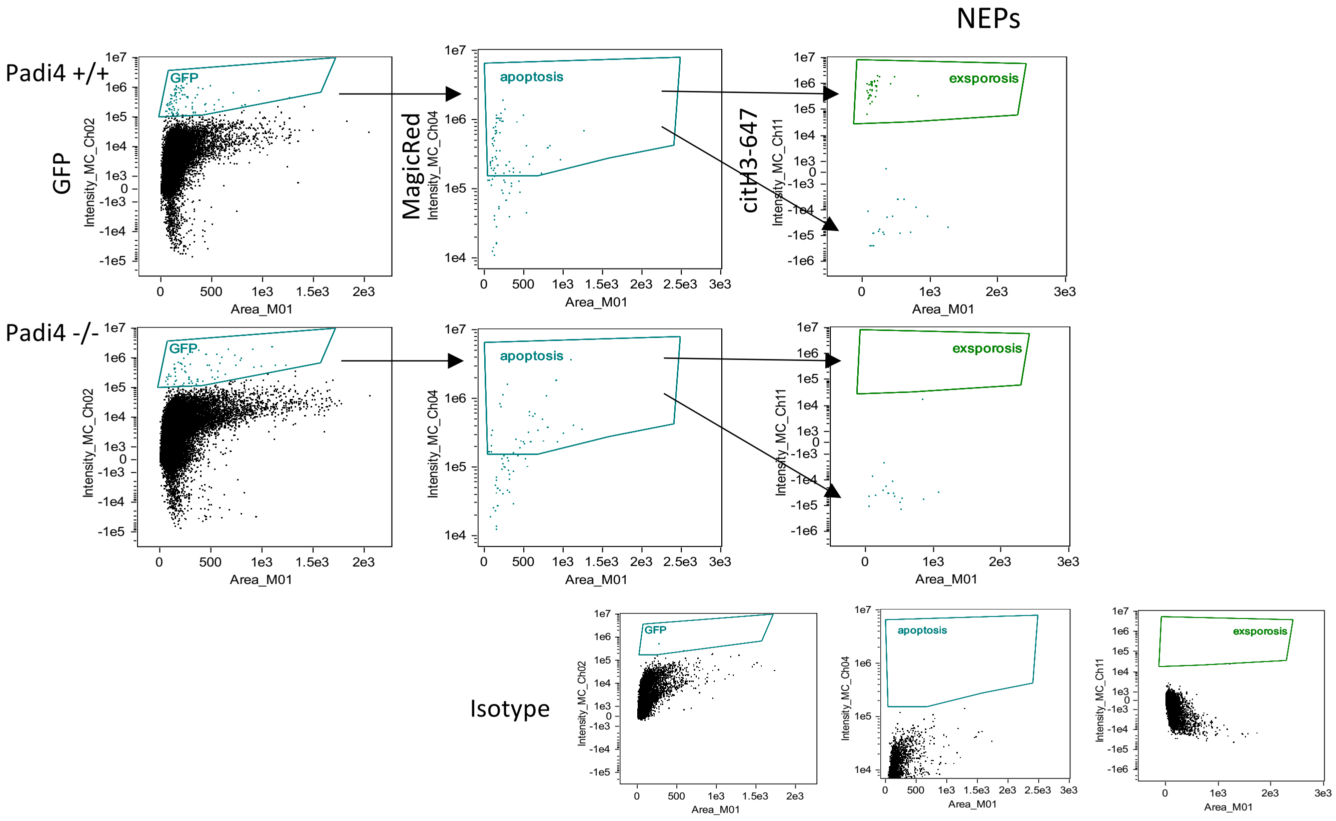

Supplementary Figure 1. Gating strategy of imagestream in Figure 3a, b and Extended Data Figure 4a

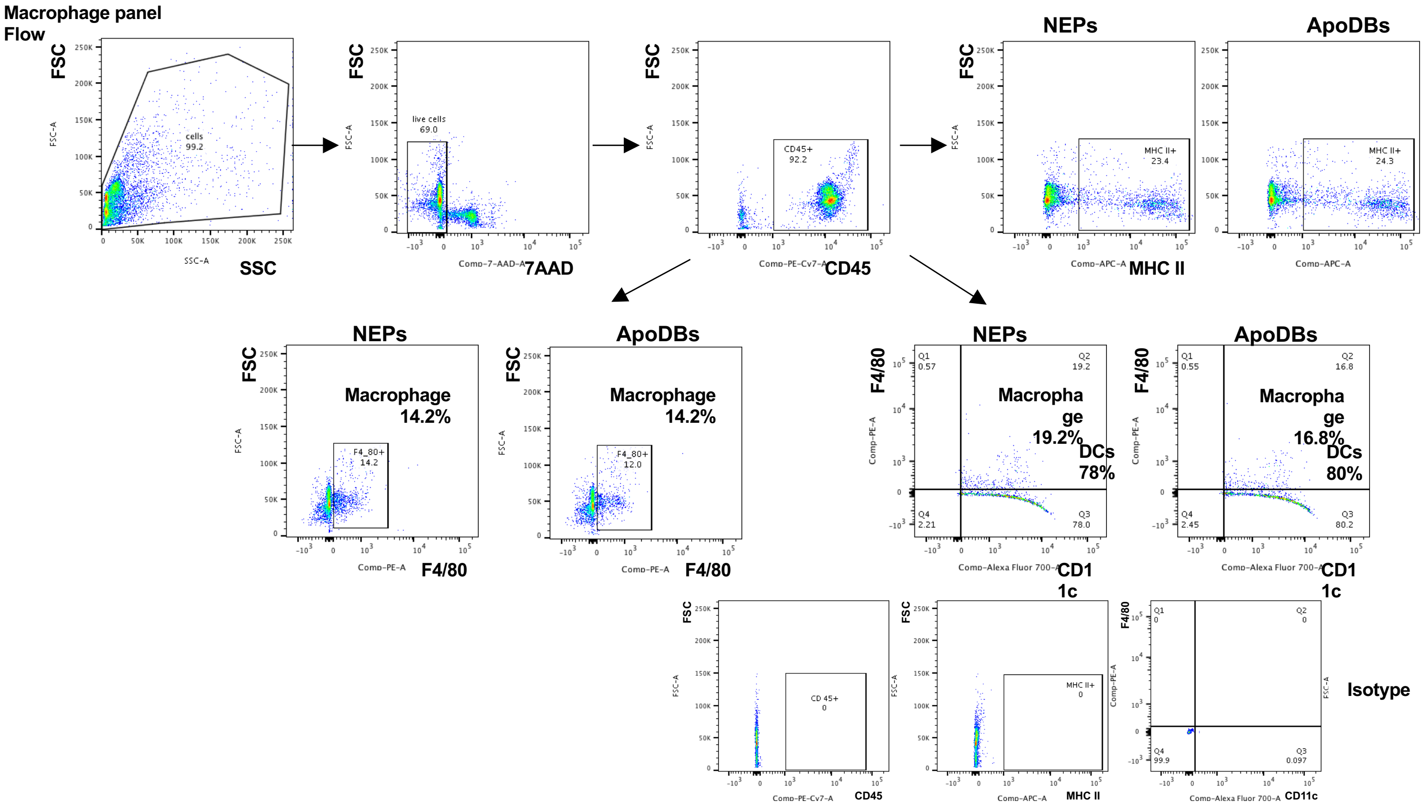

Supplementary Figure 2. Macrophage panel gating strategy of Flow cytometry in Extended Data Figure 6f

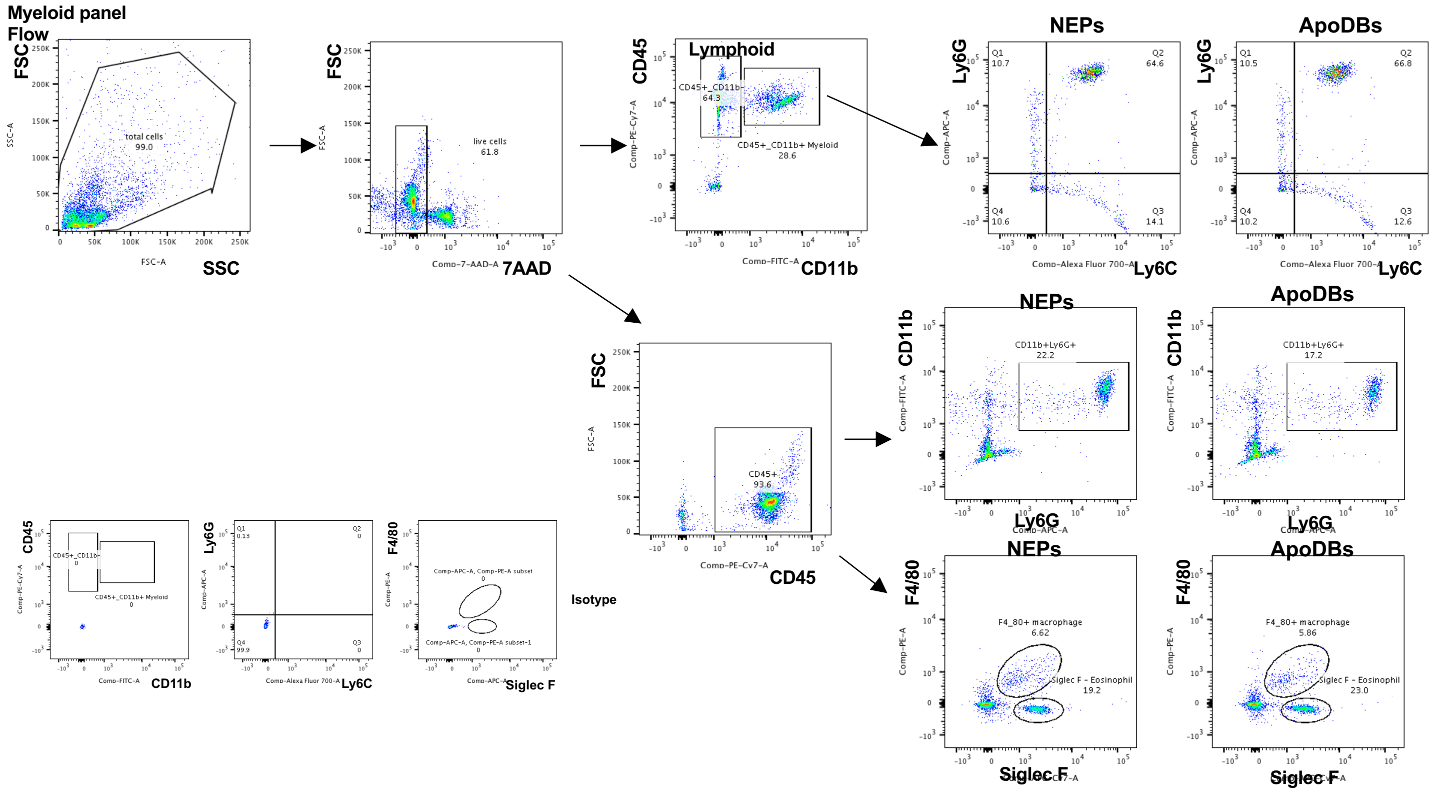

Supplementary Figure 3. Myeloid panel gating strategy of Flow cytometry in Extended Data Figure 6f

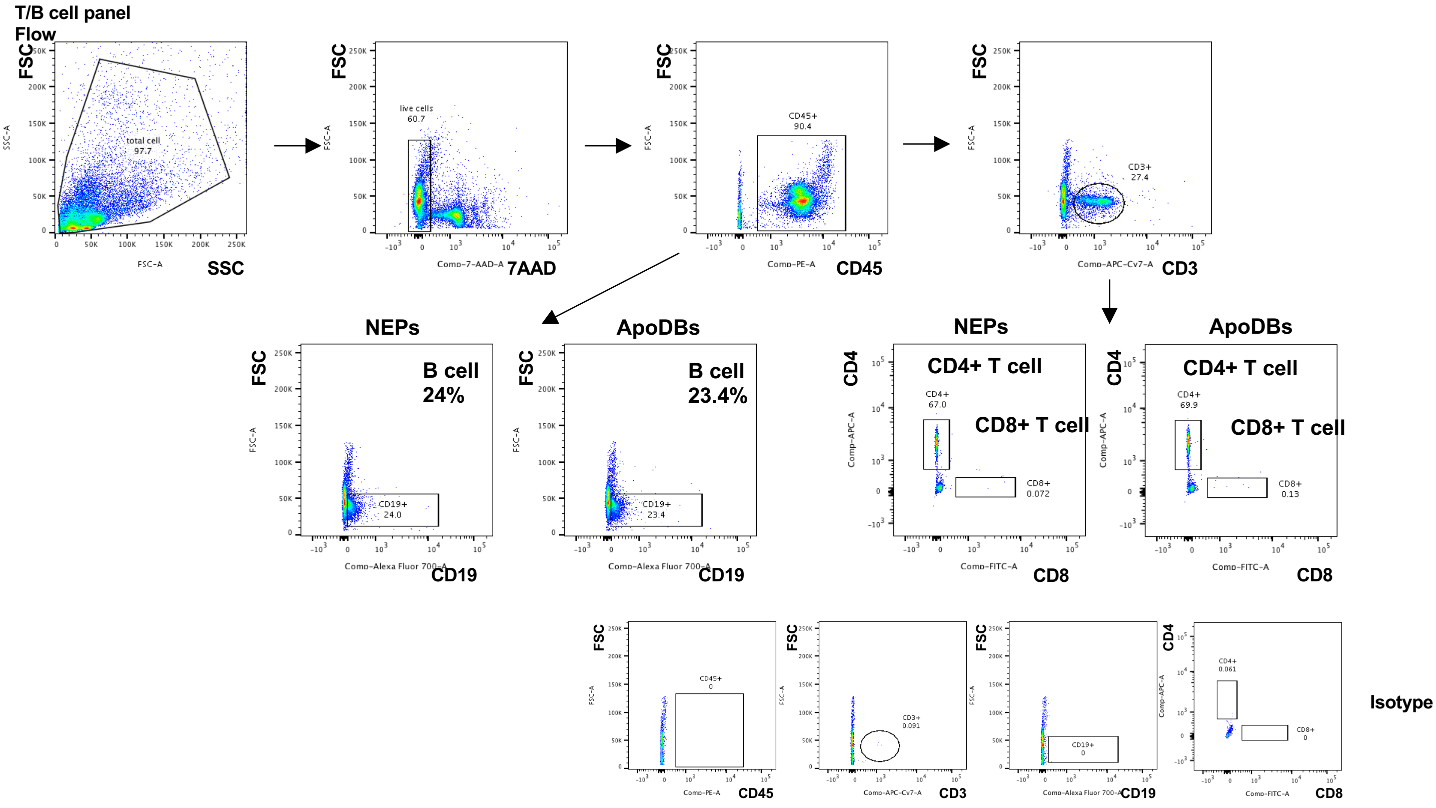

Supplementary Figure 4. T/B cell panel gating strategy of Flow cytometry in Extended Data Figure 6f

Supplementary Table 1. List of cell death inducing reagents

Drug	Description	Tumor cell nuclear expulsion	NET formation
A23187 Ionophore	Calcium ionophore that increases calcium permeability and allows extracellular calcium to flow into cells	Yes	Yes
PAF	Platelets-activating factor is a phospholipid mediator is well-known for its ability to cause platelet aggregation or inflammation	Yes	Yes
Raptinal	A cell-permeable bifluorene-decarbaldehyde that acts as a rapid activator of mitochondrial pathway-mediated intrinsic apoptosis	Yes	No
Staurosporine	Staurosporine is a protein kinase inhibitor and canonical apoptosis inducer	Yes	No
TNF-α	An inflammatory cytokine that can prime neutrophils to undergo NET formation and also apoptosis of many cell types	No	Yes
PMA	Phorbol myristate acetate, phorbol ester, is PKC activator	No	Yes

Supplementary Table 2. List of Caspase inhibitors and calcium chelators to inhibit apoptosis or to block Padi4 function

Drug	Description	Tumor cell nuclear expulsion	NET formation
GSK-484	A benzoimidazole derivative, inhibits enzymatic activities of Padi4	Blocks	Blocks
Sivelestat	Selective, reversible and competitive neutrophil elastase inhibitor	No effect	Blocks
Q-VD-OPh	Quinoline-Val-Asp-Difluorophenoxymethylketone, a potent pan-caspase inhibitor that inhibits caspases 1, 3, 4 and 9	Blocks apoptosis induces nuclear expulsion	No effect
Z-LEHD-FMK	Cell pemeable fluoromethyl ketone (FMK)- derivatized peptide that acts as an effective irreversible caspase 9 inhibitor	Blocks apoptosis induces nuclear expulsion	No effect
EGTA	Aminopolycarboxylic acid, a chelating agent that sequesters extracellular calcium	Blocks	Blocks
BAPTA-AM	A cell-permeablet chelator, highly selective for intracellular calcium	Blocks	Blocks

Supplementary Table 3. List of primers

Genes	Sequences
For sgRNAs,	
Padi4 #1	Fw-5’CACCG-CCGAAGGTTGTGTAGCCCTT’3
	Rv-5’AAAC-AGGGCTACACAACCTTCGG’3
Padi4 #2	Fw-5’CACCG-TAGGTTCGAGTTTCATACTA’3
	Rv-5’AAAC-TAGTATGAAACTCGAACCTA’3
Fir siRNAs,	
Hmgb1 #1	Fw-5’GGACAAGGCCCGUUAUGAA’3
	Rv-5’UUCAUAACGGGCCUUGUCC’3
Hmgb1 #2	Fw-5’CCCGUUAUGAAAGAGAAAU’3
	Rv-5’AUUUCUCUUUCAUAACGGG’3
Hmgb2 #1	Fw-5’CAUCUGCCUUCUUCCUGUU’3
	Rv-5’AACAGGAAGAAGGCAGAUG’3
Hmgb2 #2	Fw-5’CCGUCAAUUUCGCGGAAUU’3
	Rv-5’AAUUCCGCGAAAUUGACGG’3
Hmgb3 #1	Fw-5’CUGUAUCAAAGUUGUACAU’3
	Rv-5’AUGUACAACUUUGAUACAG’3
Hmgb3 #2	Fw-5’GGCAGAUAAAGUGCGCUAU’3
	Rv-5’AUAGCGCACUUUAUCUGCC’3
For genotype,	
Padi4	Fw-5’AGCTTTGTAAGGGGCATCCT’3
	Rv-5’TCAAAGTACCTGATGTGTTGACTG’3
For qPCR,	
Mouse	
Padi1	F- 5’TCCTGGGTCCTGACTTTGGA’3
	R-5’CCAGGCCAGAGGCACCT’3
Padi2	F- 5’AGCAGCGGAGGGCTTAC’3
	R-5’CACGCGGCTCCCATACT’3
Padi3	F- 5’TGCAGGCGCCACACAA’3
	R-5’CCTGCAGCTCTCCGTTCCT’3
Padi4	F- 5’TGACCAATGGATGCAGGACG’3
	R-5’CTCTGTCCCTCGGGGAGTC’3
Padi6	F- 5’TCTTCGCGAGGCCGTACT’3
	R-5’GGATTCCAAGGTTCTTGCCC’3
Elane	F- 5’GTTGGGCACAAACAGACC’3
	R-5’GCAAACTCAGCCACAGG’3
Mpo	F- 5’TCCCACTCAGCAAGGTCTT’3
	R-5’TAAGAGCAGGCAAATCCAG’’3
Gapdh	F- 5’AATGTGTCCGTCGTGGATCTGA’3
	R-5’GATGCCTGCTTCACCACCTTCT’3
Human	
Padi4	F- 5’GTTTAGGGTCAGACAGTCCTGG’3
	R-5’AGATGTGAGTAGTGGCACATGC’3
Gapdh	F- 5’GGAAGGTGAAGGTCGGAGTC’3
	R-5’GTTGAGGTCAATGAAGGGGTC’3
