# Supplementary material for: Apoptosis-induced nuclear expulsion in tumor cells drives S100a4-mediated metastatic outgrowth through the RAGE pathway
Source: Nat Cancer. 2023 Mar 27;4(3):419–35. doi: 10.1038/s43018-023-00524-z (PMC10042736; doi:10.1038/s43018-023-00524-z)
Supplement: Supplementary file 2 — Reporting Summary [file 43018_2023_524_MOESM2_ESM.pdf]

nature portfolio

Corresponding author(s): Li Yang

Last updated by author(s): Jan 18, 2023

Reporting Summary

Nature Portfolio wishes to improve the reproducibility of the work that we publish. This form provides structure for consistency and transparency in reporting. For further information on Nature Portfolio policies, see our Editorial Policies and the Editorial Policy Checklist.

Statistics

For all statistical analyses, confirm that the following items are present in the figure legend, table legend, main text, or Methods section.

n/a	Confirmed	
☐	☒	The exact sample size (*n*) for each experimental group/condition, given as a discrete number and unit of measurement
☐	☒	A statement on whether measurements were taken from distinct samples or whether the same sample was measured repeatedly
☐	☒	The statistical test(s) used AND whether they are one- or two-sided<br>*Only common tests should be described solely by name; describe more complex techniques in the Methods section.*
☐	☒	A description of all covariates tested
☐	☒	A description of any assumptions or corrections, such as tests of normality and adjustment for multiple comparisons
☐	☒	A full description of the statistical parameters including central tendency (e.g. means) or other basic estimates (e.g. regression coefficient) AND variation (e.g. standard deviation) or associated estimates of uncertainty (e.g. confidence intervals)
☐	☒	For null hypothesis testing, the test statistic (e.g. *F*, *t*, *r*) with confidence intervals, effect sizes, degrees of freedom and *P* value noted<br>*Give P values as exact values whenever suitable.*
☒	☐	For Bayesian analysis, information on the choice of priors and Markov chain Monte Carlo settings
☒	☐	For hierarchical and complex designs, identification of the appropriate level for tests and full reporting of outcomes
☒	☐	Estimates of effect sizes (e.g. Cohen's *d*, Pearson's *r*), indicating how they were calculated

Our web collection on statistics for biologists contains articles on many of the points above.

Software and code

Policy information about availability of computer code

Data collection	Image obtaining: Nikon Ti-ZE microscope with a Hamamatsu Flash 4 V 3, Nikon SoRa Spinning Disk with a Photometrics BSI sCMOS camera, Biorad Chemidoc with Image Lab v6.0.1.<br>Flow Cytometry: BD FACSDIVA software v8.0.1, INSPIRE™ Software<br>qPCR: Quantstudio 6 Flex Real-Time PCR System
Data analysis	Statistical analysis: Prism v9, R v3.5.1<br>Image analysis: ImageJ2 2.3.0, Imaris 9.8 software<br>Flow Cytometry data: FlowJo 10.8.0<br>nuclear expulsion algoritms (computer code): Matlab r2021a

For manuscripts utilizing custom algorithms or software that are central to the research but not yet described in published literature, software must be made available to editors and reviewers. We strongly encourage code deposition in a community repository (e.g. GitHub). See the Nature Portfolio guidelines for submitting code & software for further information.

Data

Policy information about availability of data

All manuscripts must include a data availability statement. This statement should provide the following information, where applicable:

- Accession codes, unique identifiers, or web links for publicly available datasets
- A description of any restrictions on data availability
- For clinical datasets or third party data, please ensure that the statement adheres to our policy

1. Expulsion algorithm has been deposited in FigShare, DOI is 10.6084/m9.figshare.14832234
2. RNA sequencing for exsporosis signature (see STAR methods) has been deposited in GEO, with accession number GSE178512
3. Exsporosi LC/MS proteomics has been deposited in massIVE with accession link: ftp://massive.ucsd.edu/MSV000087691/
password: 4T1Exspor
The public dataset that supports the findings of this study is available in GEO, KMPLOT or Oncomine with accession codes; GSE20685, GSE45255, GSE7390, GSE2603, GSE2634, GSE37745.

Human research participants

Policy information about studies involving human research participants and Sex and Gender in Research.

Reporting on sex and gender	n/a
Population characteristics	n/a
Recruitment	The tissue arrays were purchased from US Biomax.<br>Per the company: the tissues were collected under the highest ethical standards with the donor being informed completely and with their consent. We make sure we follow standard medical care and protect the donors' privacy. All human tissues are collected under HIPPA approved protocols.<br>company's link is here (Q10): https://www.tissuearray.com/FAQs
Ethics oversight	n/a

Note that full information on the approval of the study protocol must also be provided in the manuscript.

Field-specific reporting

Please select the one below that is the best fit for your research. If you are not sure, read the appropriate sections before making your selection.

☒ Life sciences ☐ Behavioural & social sciences ☐ Ecological, evolutionary & environmental sciences

For a reference copy of the document with all sections, see nature.com/documents/nr-reporting-summary-flat.pdf

Life sciences study design

All studies must disclose on these points even when the disclosure is negative.

Sample size	No statistical methods were used to pre-determine sample sizes, but our sample sizes are similar to those reported in previous publications (e.g., Cancer Res. 80(12): 2612-2627, 2020; Mol. Carcinogenesis, Jul;59(7):679-690, 2020).
Data exclusions	No data points or animals were excluded.
Replication	All results were obtained from at least two repeating biological experiments and ensured that all attempts were successful regarding the biological findings.<br>As for qPCR, each biological experiment performed with technical replicates.
Randomization	All mice used in this study were randomized before treating drugs or NEPs.
Blinding	Data collection and analysis were performed blind to the conditions of quantification in IF experiments but not performed blind to the conditions of in vivo experiments because the same observer treated and measured tumors.

Reporting for specific materials, systems and methods

We require information from authors about some types of materials, experimental systems and methods used in many studies. Here, indicate whether each material, system or method listed is relevant to your study. If you are not sure if a list item applies to your research, read the appropriate section before selecting a response.

Materials & experimental systems

n/a	Involved in the study
☐	☒ Antibodies
☐	☒ Eukaryotic cell lines
☒	☐ Palaeontology and archaeology
☐	☒ Animals and other organisms
☒	☐ Clinical data
☒	☐ Dual use research of concern

Methods

n/a	Involved in the study
☒	☐ ChIP-seq
☐	☒ Flow cytometry
☒	☐ MRI-based neuroimaging

Antibodies

Antibodies used

The following antibodies were used as primary antibody for Western blotting; Histone H3 (Cell signaling, CS3638; 1:2,000), Citrullinated Histone H3 (citrulline R2 + 8 + 17, Abcam, ab5103; 1:2,000), Citrullinated Histone H3 (citrulline R17, Abcam, ab219407; 1:1,000), Padi4 (Biolegend, 684202; 1:1,000), p-p44/42 MAP kinase (phosphorylated Erk 1/2, Cell signaling, CS9101; 1:1,000), p44/42 MAP kinase (Cell signaling, CS9102; 1:1,000), p-p38 MAP kinase (pT180/pY182, BD, 612281; 1:1,000), p38 MAP kinase (Cell signaling, CS9212; 1:1,000), p-Akt (Ser473, Cell signaling, CS9271; 1:1,000), Akt (Cell signaling, CS9272; 1:1,000), Parp1 (Cell signaling, CS9532; 1:2,000), Caspase1 (Abcam, ab179515; 1:500), Cleaved Caspase3 (Asp175, Cell signaling, CS9661; 1:1,000), Caspase3 (Cell signaling, CS9662; 1:2,000), GsdmD (Abcam, ab209845; 1:1,000), GsdmD (Cell signaling, CS39754; 1:1,000), GsdmE (Abcam, ab215191; 1:1,000), Mlkl (Proteintech, 66675; 1:1,000), p-Mlkl (phosphorylated ser 345, Abcam, ab196436; 1:1,000), S100a4 (R&D systems, MAB4138; 1:500), Hmgb1 (Thermo, MA5-17278; 1:1,000), Hmgb2 (Cell signaling, CS14163; 1:1,000), Hmgb3 (R&D systems; MAB55071; 1:1,000), Gapdh (Cell signaling, CS5174; 1:2,000), Lamin A/C (Cell signaling, CS2032; 1:1,000) Beta-actin (Santacruz, sc-69879; 1:2,000).
The following antibodies were used as primary antibody for IF; Citrullinated Histone H3 (citrulline R2 + 8 + 17, Abcam, ab5103; 1:200), Citrullinated Histone H3 (citrulline R17, Abcam, ab219407; 1:200), Citrullinated Histone H3 conjugated wih Alexa Fluor 647(citrulline R17, Abcam, ab237374; 1:200), S100a4 (R&D systems, MAB4138; 1:100), Ly6G (BD, 551459; 1:100), Mpo (R&D systems, AF3667; 1:100), GFP (Abcam, ab13970; 1:500), mCherry (Abcam, ab205402; 1:500), dsDNA (Abcam, ab27156; 1:500), Beta-actin (Santacruz, sc69879; 1:500), E-cadherin (Termo, 14-3249-82; 1:200), Hmgb3 (R&D systems; MAB55071; 1:100), Lamin A/C (Cell signaling, CS2032; 1:200).
The following antibodies were used as primary antibody for flow cytometry; CD45 (BD science, 552848), CD11b (BD science, 564454), Ly6G (BD science, 560599), F4/80 (Thermo, 12-4801-82), CD3e (BD science, 557596) and CD19 (BD science, 557958).

Validation

Antibodies were only used if validated by the manufacturer based on their website.
HIstone H3 : Dai, J. et al. (2005) Genes Dev 19, 472-88.
Citrulline H3 : Mondal S et al. Site-specific incorporation of citrulline into proteins in mammalian cells. Nat Commun 12:45 (2021).
Citrulline R17 H3 : Ghosh S et al. Neutrophils homing into the retina trigger pathology in early age-related macular degeneration. Commun Biol 2:348 (2019).
pp44/42 MAPK : Nat. commun. 2022 Oct 14;13(1):6092. doi: 10.1038/s41467-022-33842-4.
p44/42 MAPK : Marais, R. et al. (1993) Cell 73, 381-93.
pp38 MAPK : Science. 1996; 272(5268):1652-1655.
p38 MAPK : Rouse, J. et al. (1994) Cell 78, 1027-37.
PAKT : Inoki, K. et al. (2002) Nat Cell Biol 4, 648-57.
AKT : Zhou, B.P. et al. (2001) Nat Cell Biol 3, 245-52.
PARP1 : Tewari, M. et al. (1995) Cell 81, 801-809.
caspase1: Int J Mol Med 47:607-620 (2021).
clv caspase3: Nicholson, D.W. et al. (1995) Nature 376, 37-43.
caspase3 : Cell Death Differ. 2022 Nov;29(11):2218-2232.
GsdmD : Abcam - Cell Rep. 2021 Mar 9;34(10):108756, Cell signaling -Front Immunol. 2022 Aug 5;13:963582
GsdmE : Cell Rep. 2021 Apr 13;35(2):108998.
MLKL : Cell Death Discovery volume 7, Article number: 338 (2021)
pMLKL : Cell Death Differ 28:985-1000 (2021).
s100a4 : Scientific Reports, 2021;11(1):9668.
Hmgb1: using knockout, it was validated by manufacturer
Hmgb2: Elife. 2019 Dec 16;8:e49551.
Hmgb3: it was validated by manufacturer, https://www.rndsystems.com/products/human-mouse-hmgb3-antibody-546519_mab55071#product-details
GAPDH : Genes Dis. 2021 Nov 19;9(6):1701-1715.
Lamin A/C : Nat Commun. 2022 Aug 20;13(1):4906.
beta actin : Wu, YH. et al. 2022. Sci Adv. 8: eabn9912.
Ly6G: J Immunol. 1993; 151(5):2399-2408.
Mpo: Cancers, 2022;14(14):.
e-cadherin : Cell Rep. 2020 Nov 24;33(8):108409.
CD45 : Nat Med. 2000; 6(11):1212-1213.
CD11b : J Immunol. 1981; 126(1):359-364.
Ly6G for flowcytometry : J Immunol. 1993; 151(5):2399-2408.
F4/80 : Oncoimmunology. 2017 Jun 19;6(8):e1334744.
CD3e : J Exp Med. 1995; 181(1):375-380
CD19 : J Immunol. 2000; 165(12):6915-6921.

Eukaryotic cell lines

Policy information about cell lines and Sex and Gender in Research

Cell line source(s)	Murine 4T1, 67NR, 167FARN, 4T07, and E0771, as well as human MDA-MB-231 cell lines :ATCC<br>PC9 (Immuno-Biological Laboratories Co) was gifted from Joseph Amann . MDA-MB-231 LM2 and BrM2 cell line were gifted by Joan Massague. EO771-LMB cell line was gifted by Robin L. Anderson.<br>EO771 LM4 and MDA-MB-231 LM3 were generated by serial enrichment in the lung metastases from their parental lines respectively.
Authentication	Authentication has not been performed.
Mycoplasma contamination	All cell lines were mycoplasma negative.
Commonly misidentified lines (See ICLAC register)	None of commonly misidentified cell lines were used in the study.

Animals and other research organisms

Policy information about studies involving animals; ARRIVE guidelines recommended for reporting animal research, and Sex and Gender in Research

Laboratory animals	Mice, BALB/c, female, 6-8 week old.<br>Mice, C57Bl/6, female, 6-8 week old.<br>Mice, athymic nu/nu, female, 6-8 week old.<br>Mice, NOD-SCID mice , female, 6-8 week old.<br>Mice, Padi4 myeloid specific knockout (Padi4 flox/flox x LysM-Cre [B6.129P2-Lyz2tm1(cre)Ifo/J]), female, 6-8 week old.<br>12 light/12 dark cycle was used and Temperatures of 65-75°F (~18-23°C) with 40-60% humidity are maintained.
Wild animals	No wild animals were involved in the study.
Reporting on sex	female mice were only used for this study because of our focus on breast cancer occurred only in female.
Field-collected samples	No field-collected samples were used in the study.
Ethics oversight	All animal protocols were approved by National Cancer Institute's Animal Care and Use Committee.

Note that full information on the approval of the study protocol must also be provided in the manuscript.

Flow Cytometry

Plots

Confirm that:

- [x] The axis labels state the marker and fluorochrome used (e.g. CD4-FITC).
- [x] The axis scales are clearly visible. Include numbers along axes only for bottom left plot of group (a 'group' is an analysis of identical markers).
- [x] All plots are contour plots with outliers or pseudocolor plots.
- [x] A numerical value for number of cells or percentage (with statistics) is provided.

Methodology

Sample preparation	Lungs were collected from mice that received co-injection of tumor cells with NEPs or apoptotic debris. Lungs were minced and incubated with dissociation buffer (plain DMEM containing 1 mg/mL of Collagenase, 120 ug/mL of Dispase and 0.15 mg/mL) for 45 minutes with rotation (150 rpm at 37 OC). Dissociated tissues were then filtered by 70 um cell strainer and washed with MACS buffer (PBS containing 2 % FBS and 1 mM of EDTA). Red blood cells were removed by incubation with ACK buffer. After washing with MACS buffer, cells were resuspended with MACS buffer containing 7AAD followed by staining with appropriate antibodies
Instrument	Amnis ImageStream MkII (Luminex Corporation), BD FACS Aria II
Software	INSPIRE software (Luminex Corporation), BD FACSDIVA and FlowJo 10.8.0
Cell population abundance	1. Measuring apoptosis/nuclear expulsion positivity in in vivo system: positively gated GFP+ cells were ~1 %. Among GFP+

Cell population abundance	cells, MagicRed positive populations were 50~60 % and followed citH3 positive cells were ~60 or ~5 % depending on padi4<br>2. Exploring immune landscape after NEPs/apoDBs injection:
Gating strategy	1. Control stains (unstained/GFP negative, GFP positive, MagicRed positive, citH3 positive) were used to set gates. Tumor cells were selected by GFP. Dead and live cells were gated by MagicRed followed by citH3 gating to get nuclear expulsion populations<br>2. Control stains (unstained,7AAD or each antibodies single labeled cells) were used to set gates. All samples were FSC-A/SSC-A gated, followed by FSC-A/FSC-H gating to remove cell debris and select single cells. 7AAD- cells were gated as live cells, then immune cells were gated and analyzed,<br>*detailed information is described in method and Supplementary Data for reviewers.

☒ Tick this box to confirm that a figure exemplifying the gating strategy is provided in the Supplementary Information.
