# Supplementary figures and images for: Apoptosis-induced nuclear expulsion in tumor cells drives S100a4-mediated metastatic outgrowth through the RAGE pathway
Source: Nat Cancer. 2023 Mar 27;4(3):419–35. doi: 10.1038/s43018-023-00524-z (PMC10042736; doi:10.1038/s43018-023-00524-z)

Figure 1c 4T1

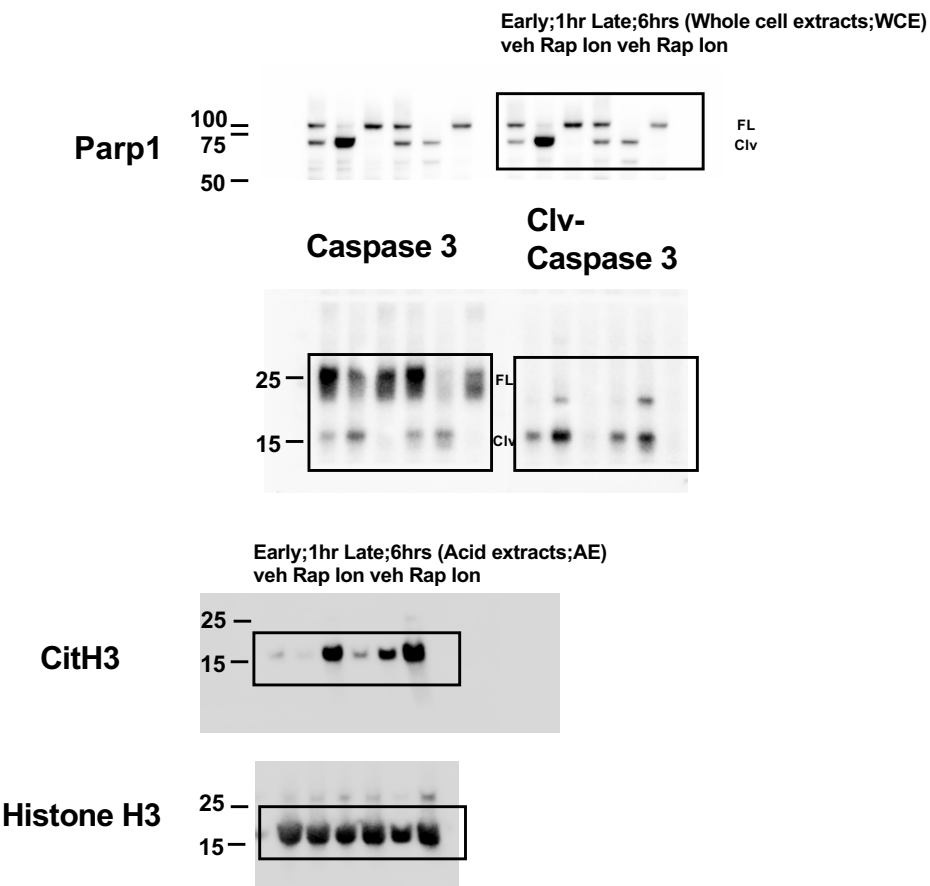

Supplement: Source Data Fig. 1 — Unprocessed western blots and/or gels. [file 43018_2023_524_MOESM20_ESM.pdf]

**Figure 5k**     **4T1**

PBS                      sRAGE  
-   NEPs   Apopt. -   NEPs Apopt.

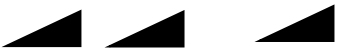

**Phospho-Erk1/2**

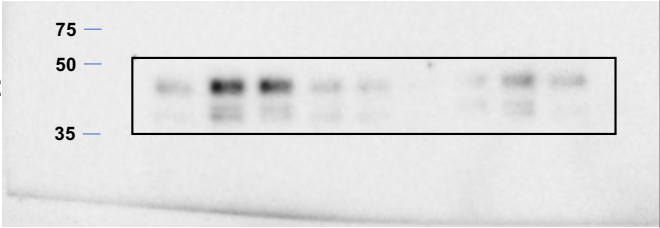

**Total Erk1/2**

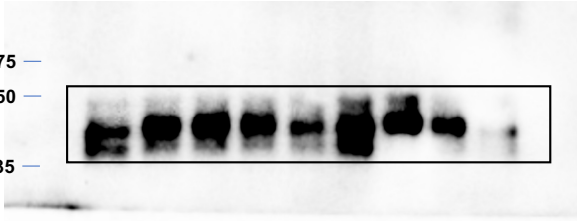

**Beta-actin**

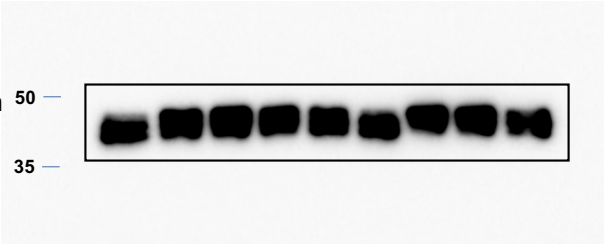

Supplement: Source Data Fig. 5 — Unprocessed western blots and/or gels. [file 43018_2023_524_MOESM26_ESM.pdf]

Figure 6a EO771

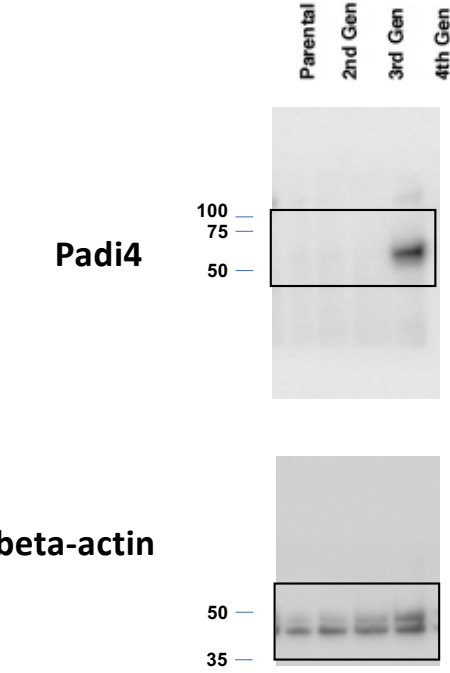

Figure 6b EO771

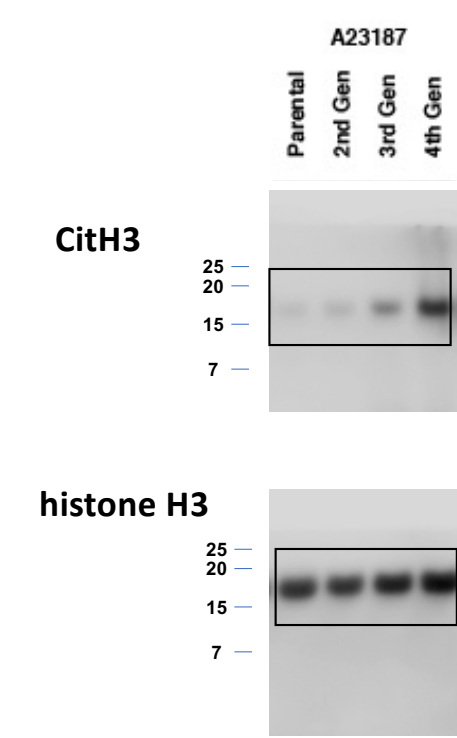

Figure 6E 4T1

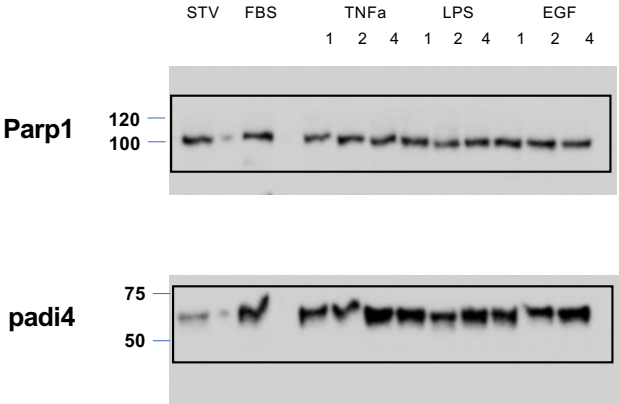

Figure 6F 4T1

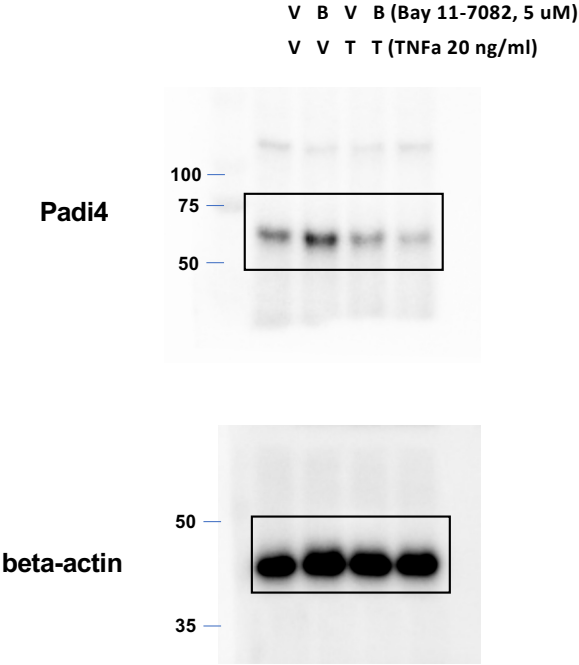

Figure 6G      4T1

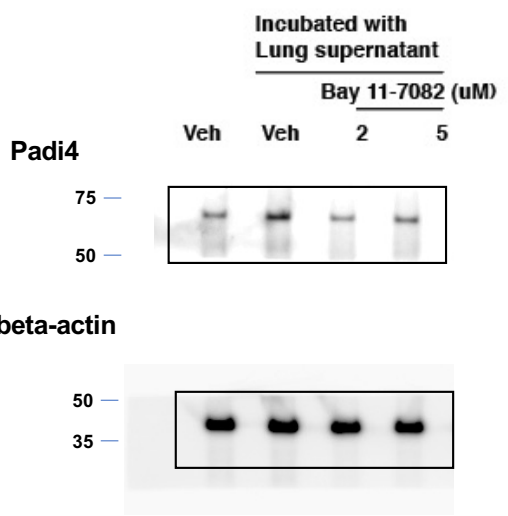

Supplement: Source Data Fig. 6 — Unprocessed western blots and/or gels. [file 43018_2023_524_MOESM28_ESM.pdf]

Extended Data Figure 3d 4T1

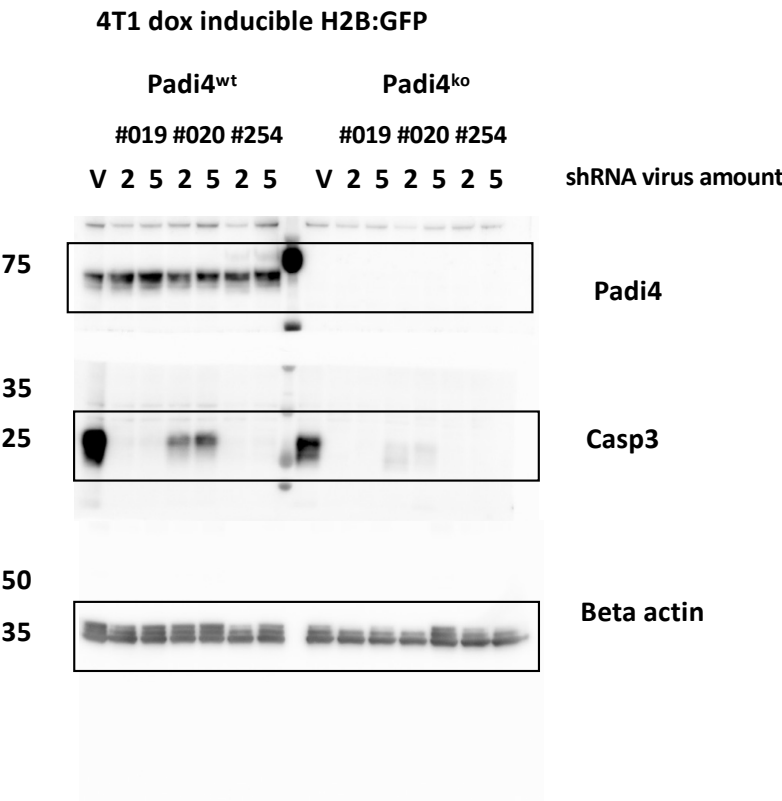

Extended Data Figure 3g and J 4T1, RAW 264.7

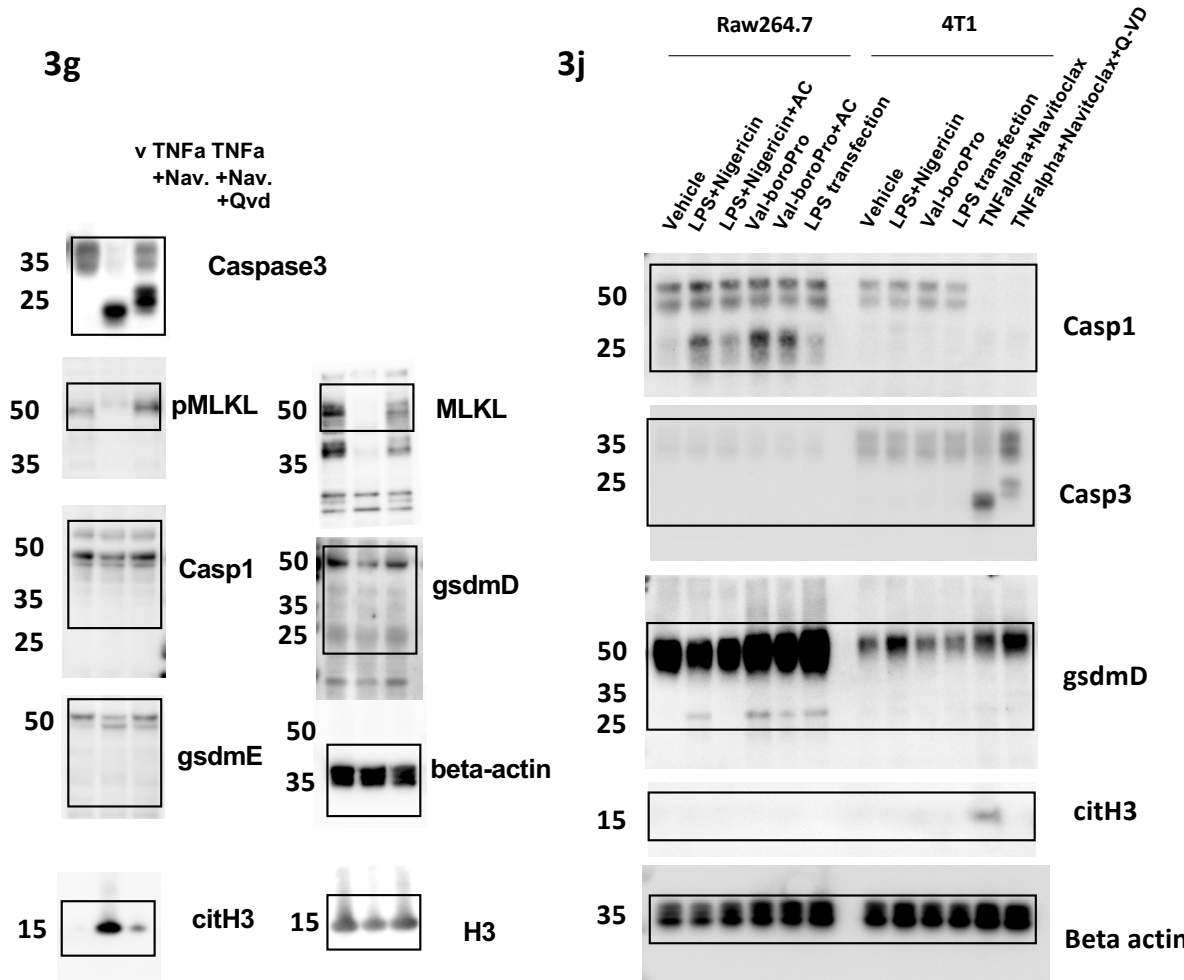

Supplement: Source Data Extended Data Fig. 3 — Unprocessed western blots and/or gels. [file 43018_2023_524_MOESM31_ESM.pdf]

## Extended Data Figure 9f

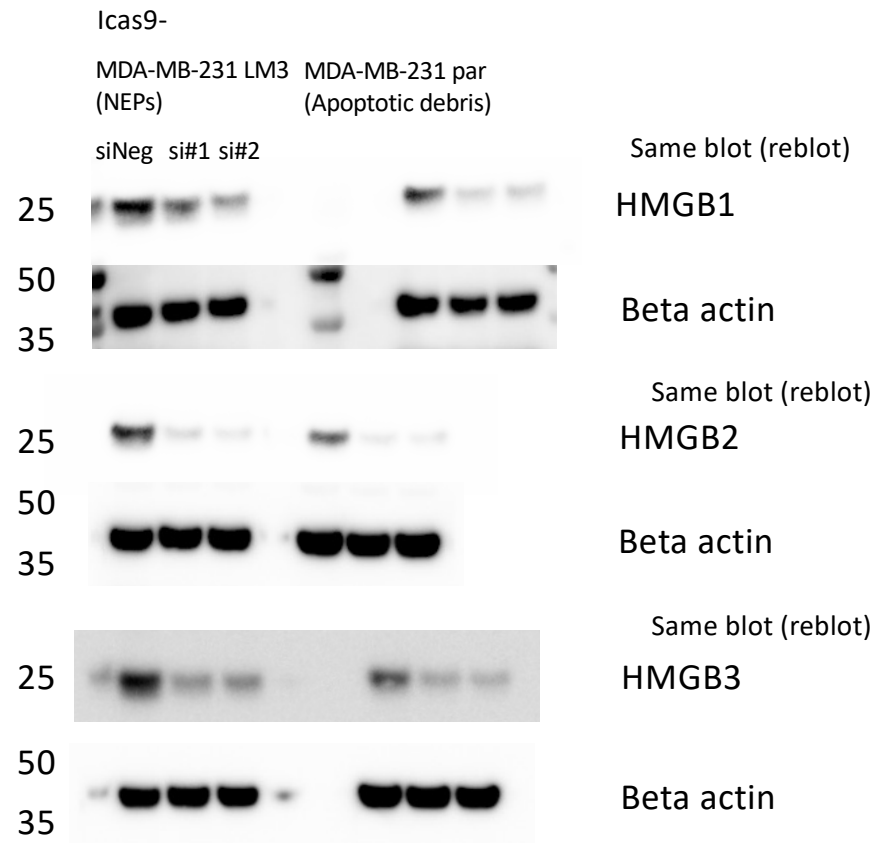

Supplement: Source Data Extended Data Fig. 9 — Unprocessed western blots and/or gels. [file 43018_2023_524_MOESM41_ESM.pdf]
