# Supplementary material for: Apoptosis-induced nuclear expulsion in tumor cells drives S100a4-mediated metastatic outgrowth through the RAGE pathway
Source: Nat Cancer. 2023 Mar 27;4(3):419–35. doi: 10.1038/s43018-023-00524-z (PMC10042736; doi:10.1038/s43018-023-00524-z)
Supplement: Source Data Fig. 2 — Unprocessed western blots and/or gels. [file 43018_2023_524_MOESM22_ESM.pdf]

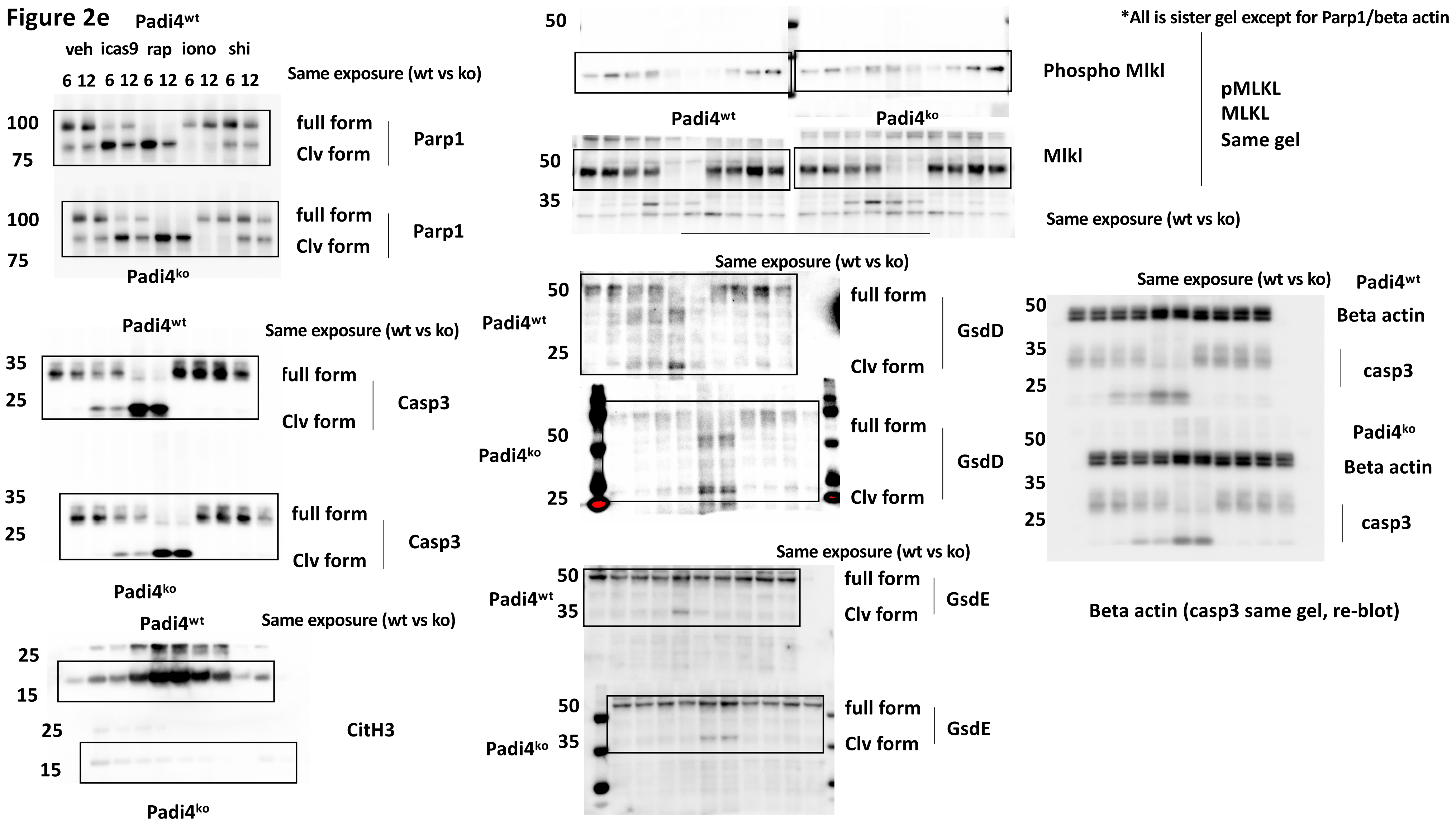
Figure 2e
Padi4wt
veh icas9 rap iono shi
6 12 6 12 6 12 6 12 6 12
Same exposure (wt vs ko)
100
75
full form
Clv form
Parp1
100
75
full form
Clv form
Parp1
Padi4ko
Padi4wt
Same exposure (wt vs ko)
35
25
full form
Clv form
Casp3
35
25
full form
Clv form
Casp3
Padi4ko
Padi4wt
Same exposure (wt vs ko)
25
15
25
15
CitH3
Padi4ko
50
Phospho Mlkl
Padi4wt
Padi4ko
50
35
Mlkl
pMLKL
MLKL
Same gel
Same exposure (wt vs ko)
*All is sister gel except for Parp1/beta actin
Same exposure (wt vs ko)
50
25
Padi4wt
full form
Clv form
GsdD
50
25
Padi4ko
full form
Clv form
GsdD
Same exposure (wt vs ko)
50
35
Padi4wt
full form
Clv form
GsdE
50
35
Padi4ko
full form
Clv form
GsdE
Same exposure (wt vs ko)
Padi4wt
50
Beta actin
35
25
casp3
Padi4ko
50
Beta actin
35
25
casp3
Beta actin (casp3 same gel, re-blot)
