# Supplementary material for: Apoptosis-induced nuclear expulsion in tumor cells drives S100a4-mediated metastatic outgrowth through the RAGE pathway
Source: Nat Cancer. 2023 Mar 27;4(3):419–35. doi: 10.1038/s43018-023-00524-z (PMC10042736; doi:10.1038/s43018-023-00524-z)

Extended Data Figure 1b 4T1

CitH3

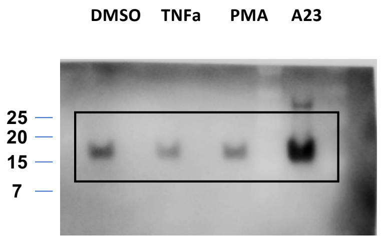

histone H3

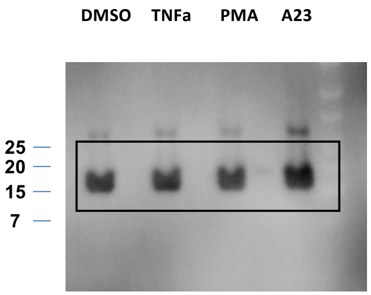

Extended Data Figure 1c

MDA231LM3, PC9 (PC-14), H322M, RT112, SW780

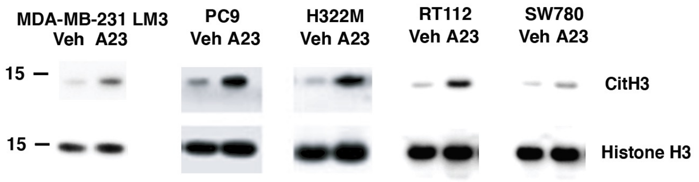

MDA-MB-231-LM3

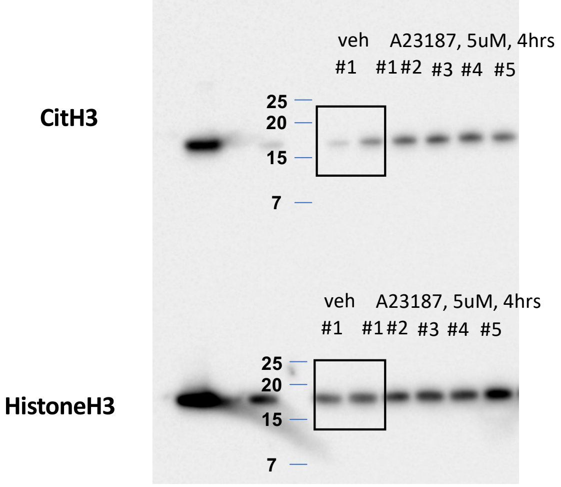

RT112 **SW780**

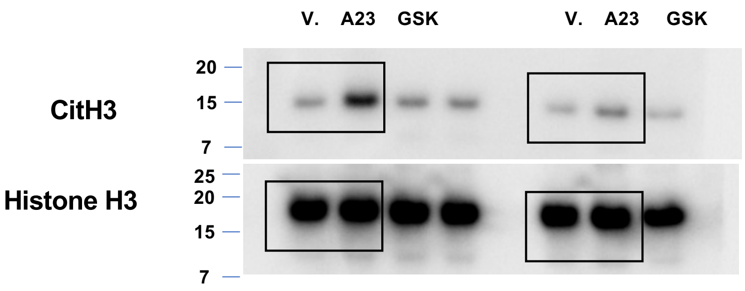

PC9

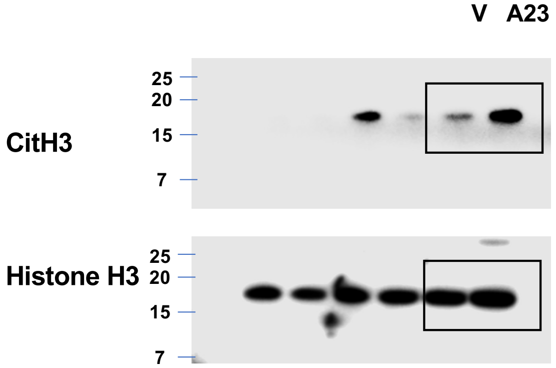

H322M

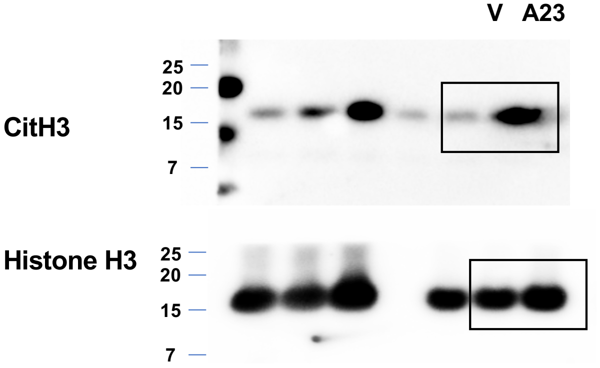

Extended Data Figure 1d 4T1

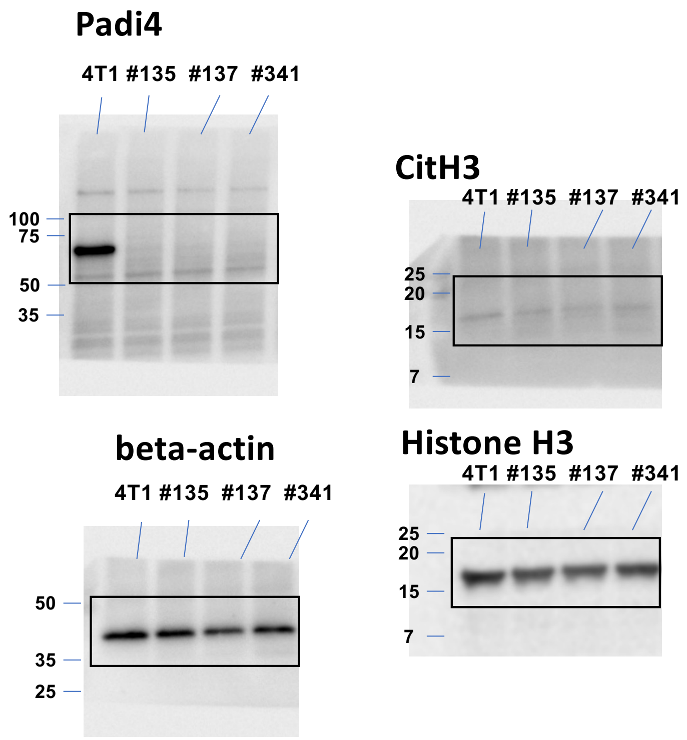

Extended Data Figure 1e 4T1

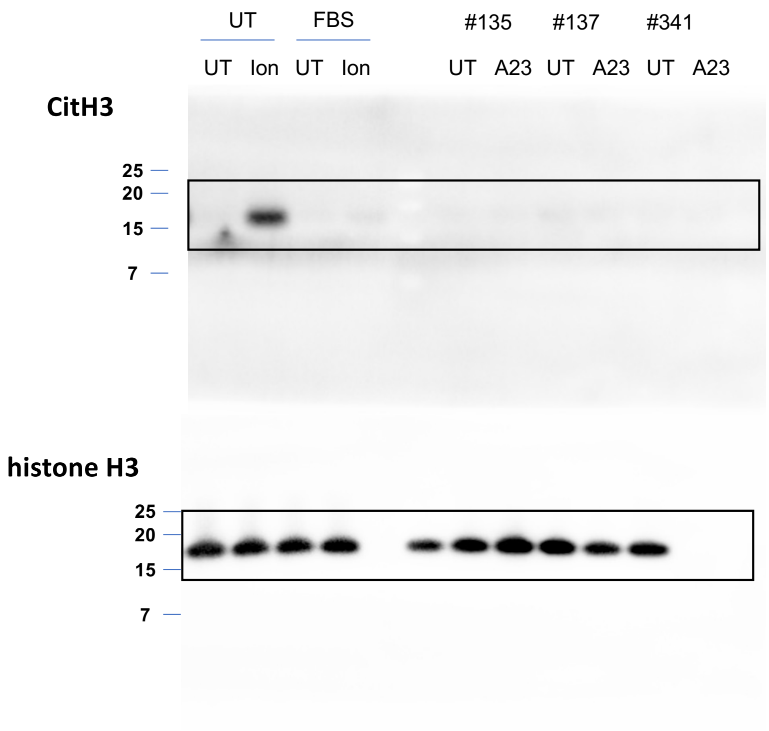

Extended Data Figure 1f

4T1 derived NEPs or ApoDBs

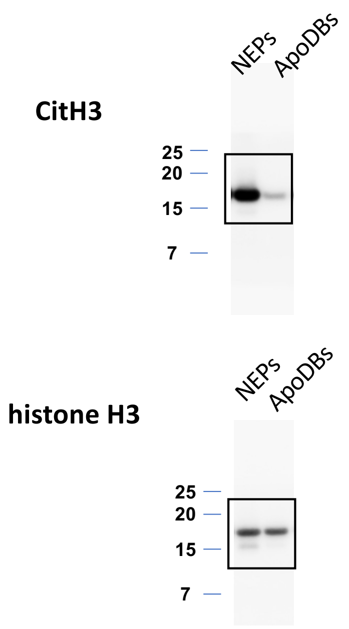

Supplement: Source Data Extended Data Fig. 1 — Unprocessed western blots and/or gels. [file 43018_2023_524_MOESM29_ESM.pdf]
