# Supplementary material for: Apoptosis-induced nuclear expulsion in tumor cells drives S100a4-mediated metastatic outgrowth through the RAGE pathway
Source: Nat Cancer. 2023 Mar 27;4(3):419–35. doi: 10.1038/s43018-023-00524-z (PMC10042736; doi:10.1038/s43018-023-00524-z)

Extended Data Figure 5d   EO771 LMB

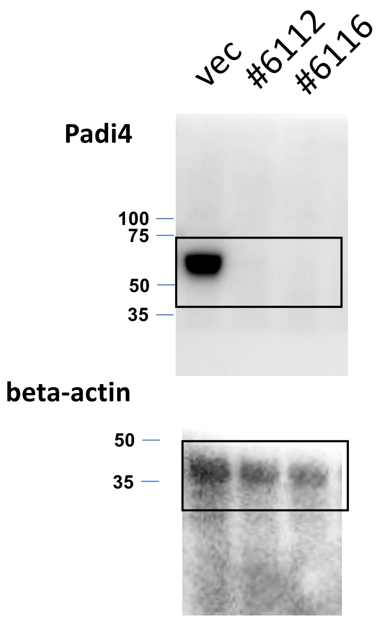

Extended Data Figure 5f   EO771 LMB

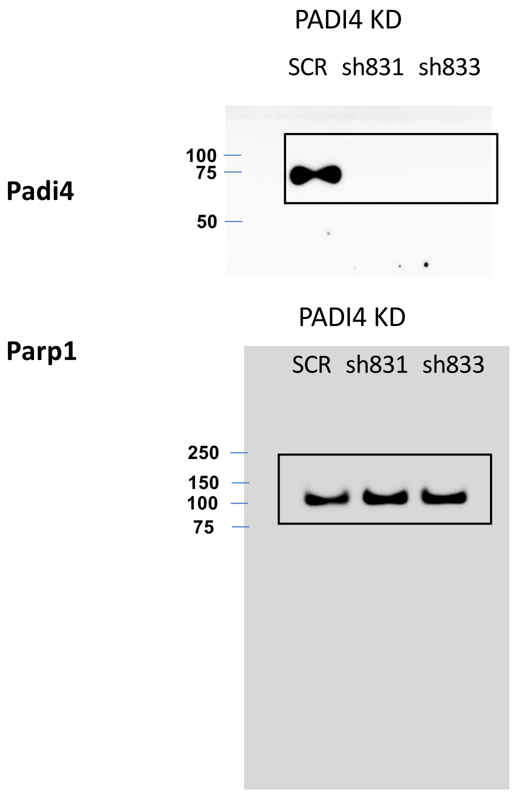

Extended Data Figure 5g

EO771 LMB

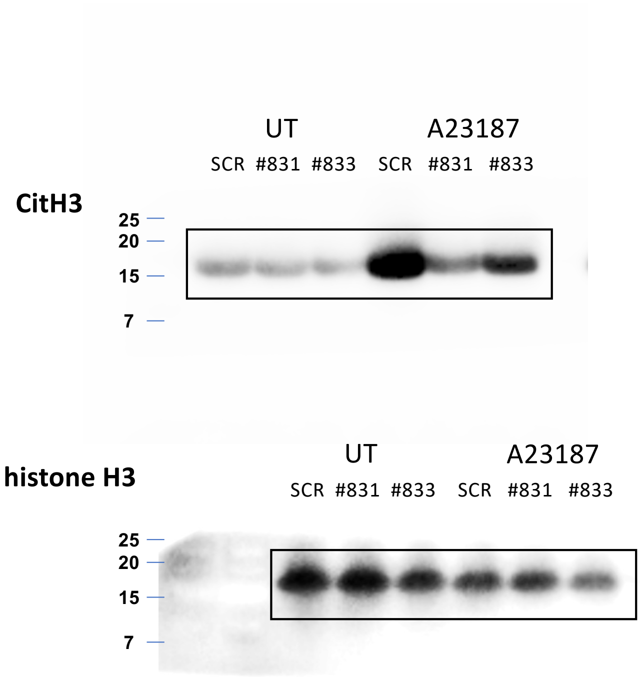

Extended Data Figure 5h

myeloid cells (LY6G+ neutrophils)

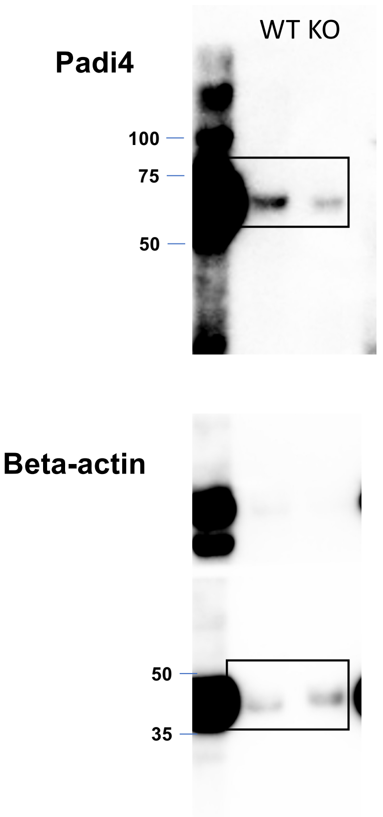

Extended Data Figure 5i

myeloid cells (LY6G+ neutrophils from PB)

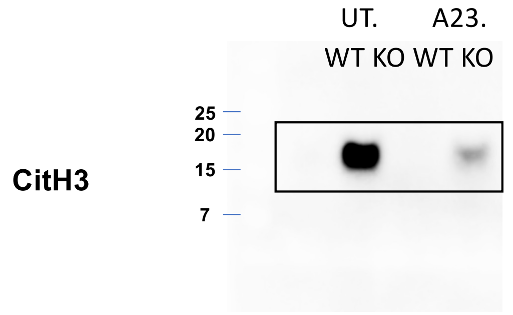

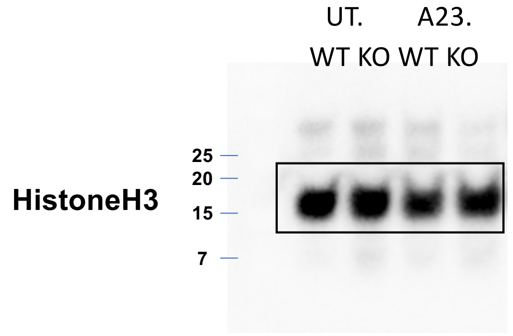

Supplement: Source Data Extended Data Fig. 5 — Unprocessed western blots and/or gels. [file 43018_2023_524_MOESM34_ESM.pdf]
