# Supplementary material for: Apoptosis-induced nuclear expulsion in tumor cells drives S100a4-mediated metastatic outgrowth through the RAGE pathway
Source: Nat Cancer. 2023 Mar 27;4(3):419–35. doi: 10.1038/s43018-023-00524-z (PMC10042736; doi:10.1038/s43018-023-00524-z)

Extended Data Figure 7b

chromatin from 4T1 plus rcS100a4

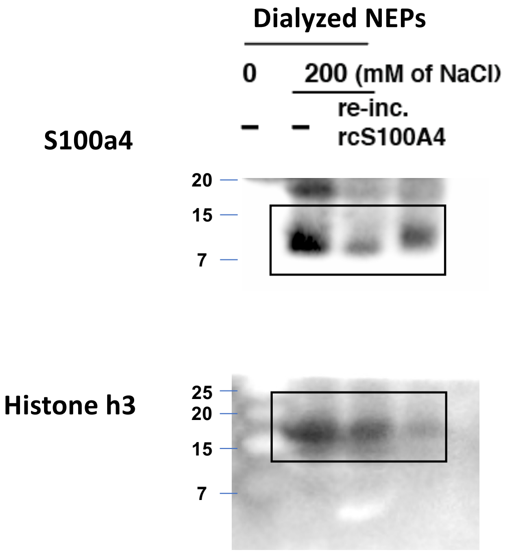

Extended Data Figure 7f

4T1 **1.5 ug/lane**

N: NEPs
A: Apoptotic debris

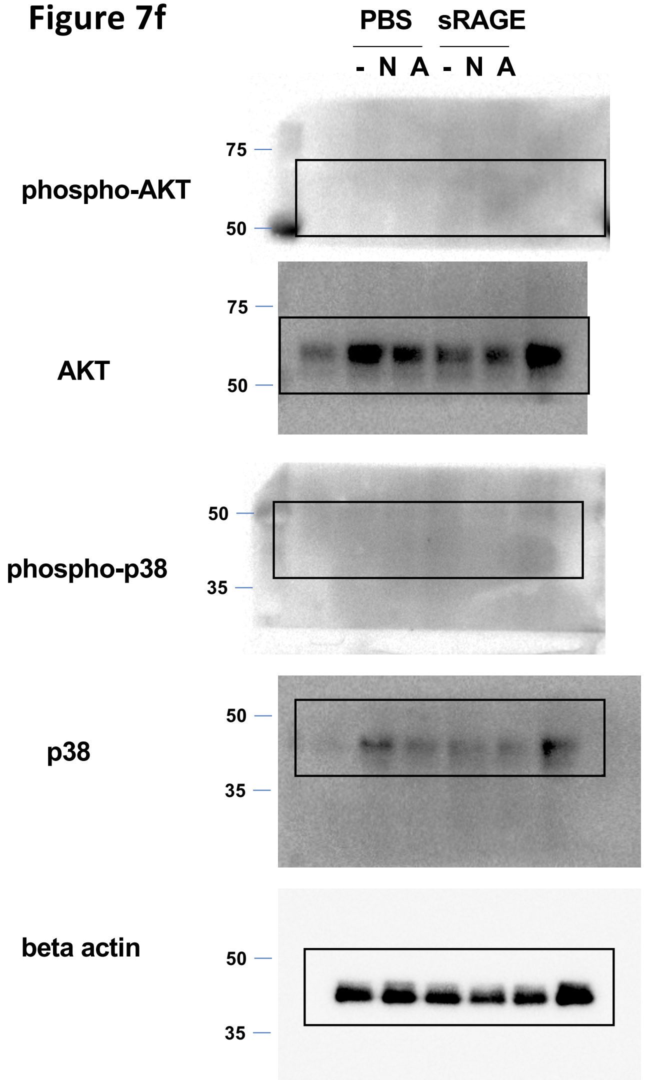

Additional info

4T1, starved

5 ug/lane, long exposure, but no total

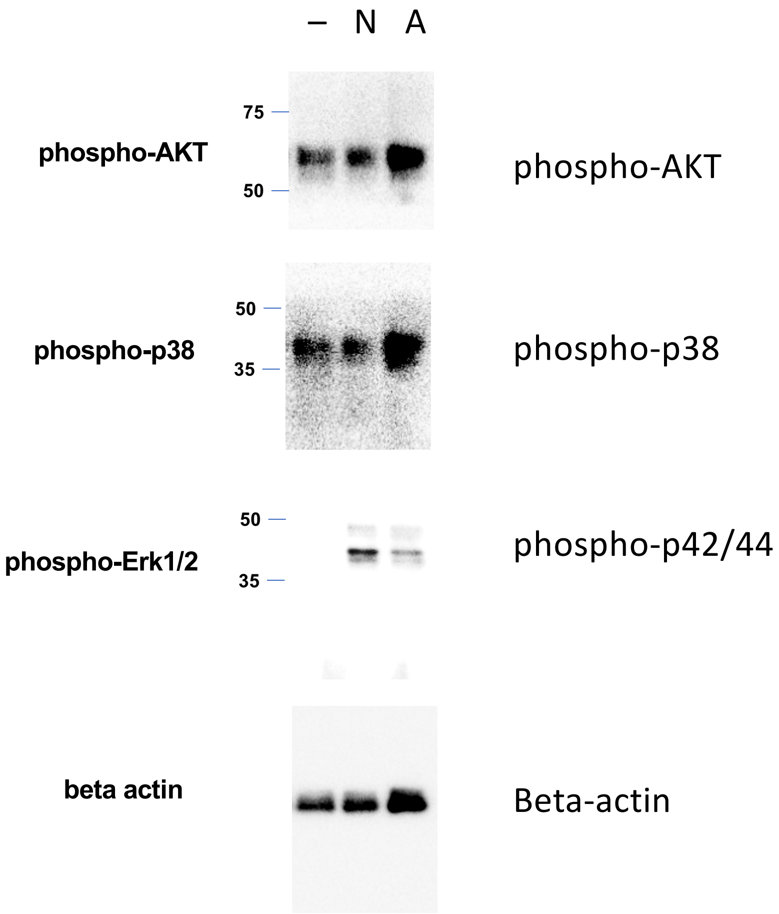

Supplement: Source Data Extended Data Fig. 7 — Unprocessed western blots and/or gels. [file 43018_2023_524_MOESM37_ESM.pdf]
