# Supplementary material for: Apoptosis-induced nuclear expulsion in tumor cells drives S100a4-mediated metastatic outgrowth through the RAGE pathway
Source: Nat Cancer. 2023 Mar 27;4(3):419–35. doi: 10.1038/s43018-023-00524-z (PMC10042736; doi:10.1038/s43018-023-00524-z)

Extended Data Figure 8b

MDA-MB-231

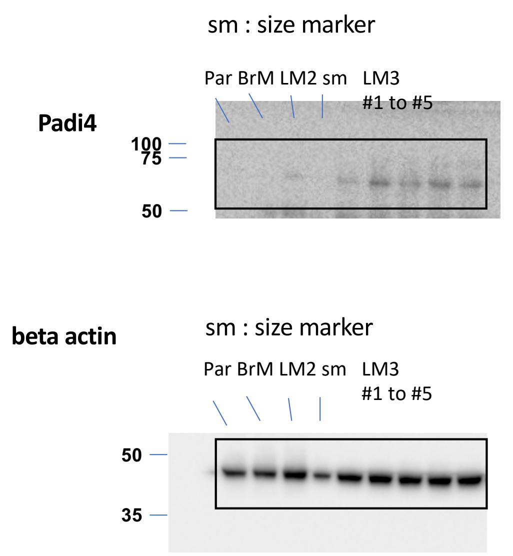

Extended Data Figure 8c

Murine cell lines

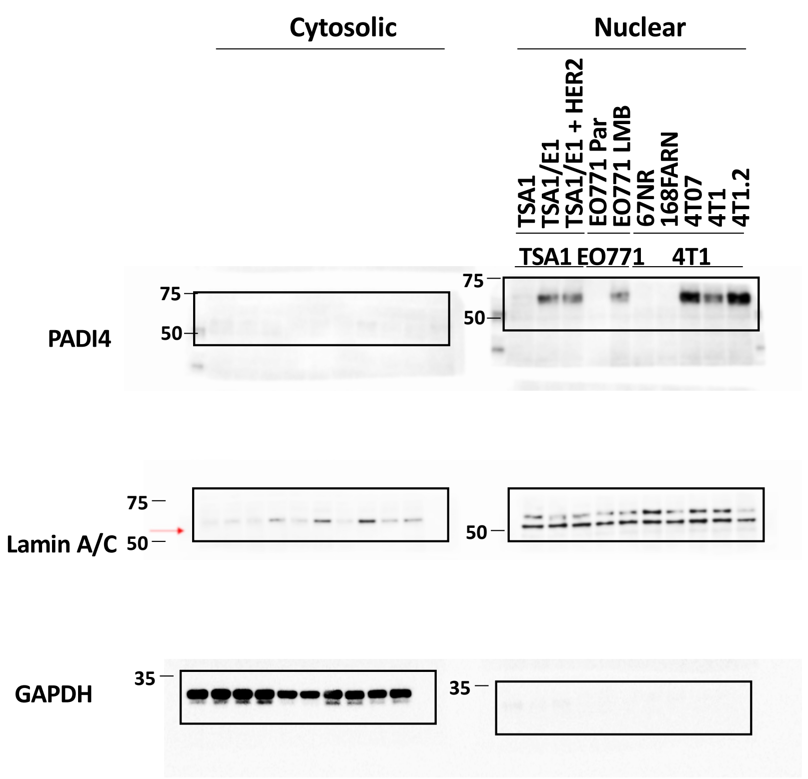

Extended Data Figure 8g **4T1**

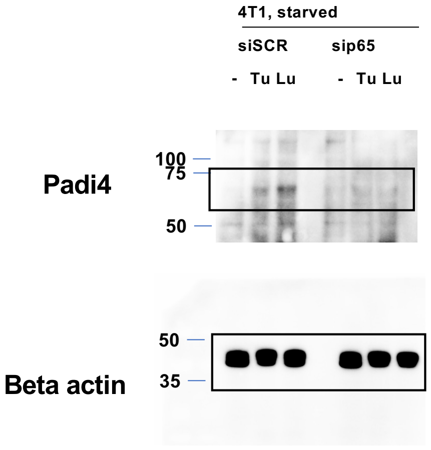

Extended Data Figure 8h **4T1**

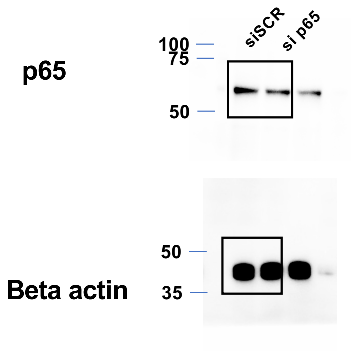

Supplement: Source Data Extended Data Fig. 8 — Unprocessed western blots and/or gels. [file 43018_2023_524_MOESM39_ESM.pdf]
